# Colorless Polyimides Derived from Novel Role-Dividing *Spiro*-Type Monomers: Strategies to Overcome the Trade-Off Between Low Linear Coefficients of Thermal Expansion and Low Thickness-Direction Birefringence Without Fillers

**DOI:** 10.3390/polym18091108

**Published:** 2026-04-30

**Authors:** Masatoshi Hasegawa, Yoshihiko Terada, Ko Nagahaba, Soichi Tsukuda, Toya Ikuma, Hikaru Sugihara, Ryosuke Masaka, Shinya Takahashi, Junichi Ishii, Takao Miwa

**Affiliations:** Department of Chemistry, Faculty of Science, Toho University, 2-2-1 Miyama, Funabashi 274-8510, Chiba, Japan

**Keywords:** colorless polyimides, role-dividing *spiro*-type monomers, optical transparency, heat resistance, linear coefficients of thermal expansion (CTE), thickness-direction birefringence (∆*n*_th_), solution processability, plastic substrates for flexible displays

## Abstract

This study presents unique polymeric materials applicable to plastic substrates for use in flexible-display devices that overcome the trade-off between low linear coefficients of thermal expansion (CTE) and low thickness-direction birefringence (Δ*n*_th_) while combining a very high *T*_g_, sufficiently high thermal stability, excellent optical transparency, good solubility, and minimum-required ductility. Polyimide (PI) films obtained from 1,2,3,4-cyclobutanetetracarboxylic dianhydride (CBDA) with 2,2′-bis(trifluoromethyl)benzidine (TFMB) under different conditions resulted in widely varying CTE values and provided a clear CTE–Δ*n*_th_ correlation, which can be regarded as a virtual lower boundary in the CTE–Δ*n*_th_ relationship for various PI systems. The pristine CBDA/TFMB and CpODA/TFMB (CpODA = norbornane-2-*spiro*-*α*-cyclopentanone-*α*′-*spiro*-2″-norbornane-5,5″,6,6″-tetracarboxylic dianhydride) systems were modified using numerous specifically designed monomers, i.e., a vertical-alignment-type liquid-crystalline diamine and *cardo*-type and *spiro*-type monomers. However, it was very challenging to overcome the trade-off between low CTE and low Δ*n*_th_, that is, to significantly exceed this lower boundary by modifying the pristine systems, while ensuring other target properties. One of the keys to achieving the present goal was compatibility with chemical imidization or one-pot polymerization processes (i.e., high solubility of the PIs), because these processes were more advantageous in reducing CTE and enhancing film transparency than the conventional two-step process. The modifications using phenyl-substituted xanthene-pendant 2,7-diaminofluorene and fluorene-pendant 2,3,6,7-xanthenetetracarboxylic dianhydride exhibited a prominent effect on overcoming the trade-off without the help of any fillers, while combining other excellent target properties. Polarized FT-IR difference spectra measured at varying incidence angles suggested that these side groups, which are connected perpendicularly to the PI main chains, align in the *Z*-direction, rationalizing the observed prominent effect. Thus, unique high-temperature transparent materials applicable to plastic substrates were successfully obtained in this study.

## 1. Introduction

In recent years, optically transparent (colorless and non-turbid) plastic substrates, alternatives to conventional heavy/fragile non-alkali glass substrates [1], have been widely investigated as a key material for flexible-display devices [2,3,4,5]. The present study focuses on the development of novel plastic substrates with unprecedented excellent combined properties for use in flexible displays, in particular, flexible liquid-crystal displays (LCDs).

Commercially available conventional colorless resins are not addressed directly to plastic substrates owing to their insufficient heat resistance (glass transition temperature, *T*_g_). For example, even poly(ether sulfone), which has the highest *T*_g_ (225 °C [6]) among current super-engineering plastics, is inadequate owing to its insufficient *T*_g_ for multiple high-temperature processes during device fabrication. Wholly aromatic polyimides (PIs) are the only polymeric materials suitable for the present purpose in terms of heat resistance. However, as typically demonstrated by commercially available PI films (KAPTON^®^ H [7] and UPILEX^®^-S films [8]), wholly aromatic PI films are intensely colored owing to charge-transfer (CT) interactions based on their electron donor–acceptor chain sequences [9,10,11,12], except for a fluorinated PI system derived from 4,4′-(hexafluoroisopropylidene)diphthalic anhydride (6FDA) with 2,2′-bis(trifluoromethyl)benzidine (TFMB) [9]. Therefore, conventional wholly aromatic PI films are also not applicable to plastic substrates.

The coloration of the PI films can be effectively suppressed by disrupting the CT interactions, specifically, by replacing one (or both) of the aromatic monomers (aromatic tetracarboxylic dianhydrides (TCDA) or aromatic diamines) with aliphatic ones [10]. Numerous optically transparent (colorless) semi- or wholly cycloaliphatic (alicyclic) PIs have been produced using this method [13,14,15,16,17,18,19,20,21,22,23,24,25,26,27,28,29,30,31,32,33,34,35,36,37,38,39,40,41,42,43,44,45,46,47,48]. However, critical challenges can occur during the production of colorless PIs; in the combinations of aromatic TCDAs with aliphatic diamines (called semi-cycloaliphatic PIs (A-type) in this paper) or aliphatic TCDAs with aliphatic diamines (wholly cycloaliphatic PIs), insoluble salts formed during the polymerization of the PI precursors (poly(amic acid)s, PAAs) hinder the smooth progress of the polymerization owing to their precipitation [32]. In contrast, the combinations of aliphatic (usually cycloaliphatic to assure heat resistance) TCDAs with aromatic diamines (called semi-cycloaliphatic PIs (B-type) in this paper) ensure smooth polymerization [47,48]. From this overwhelming manufacturing advantage, semi-cycloaliphatic colorless PIs (B-type) were the focus of this study.

**Figure 1:**
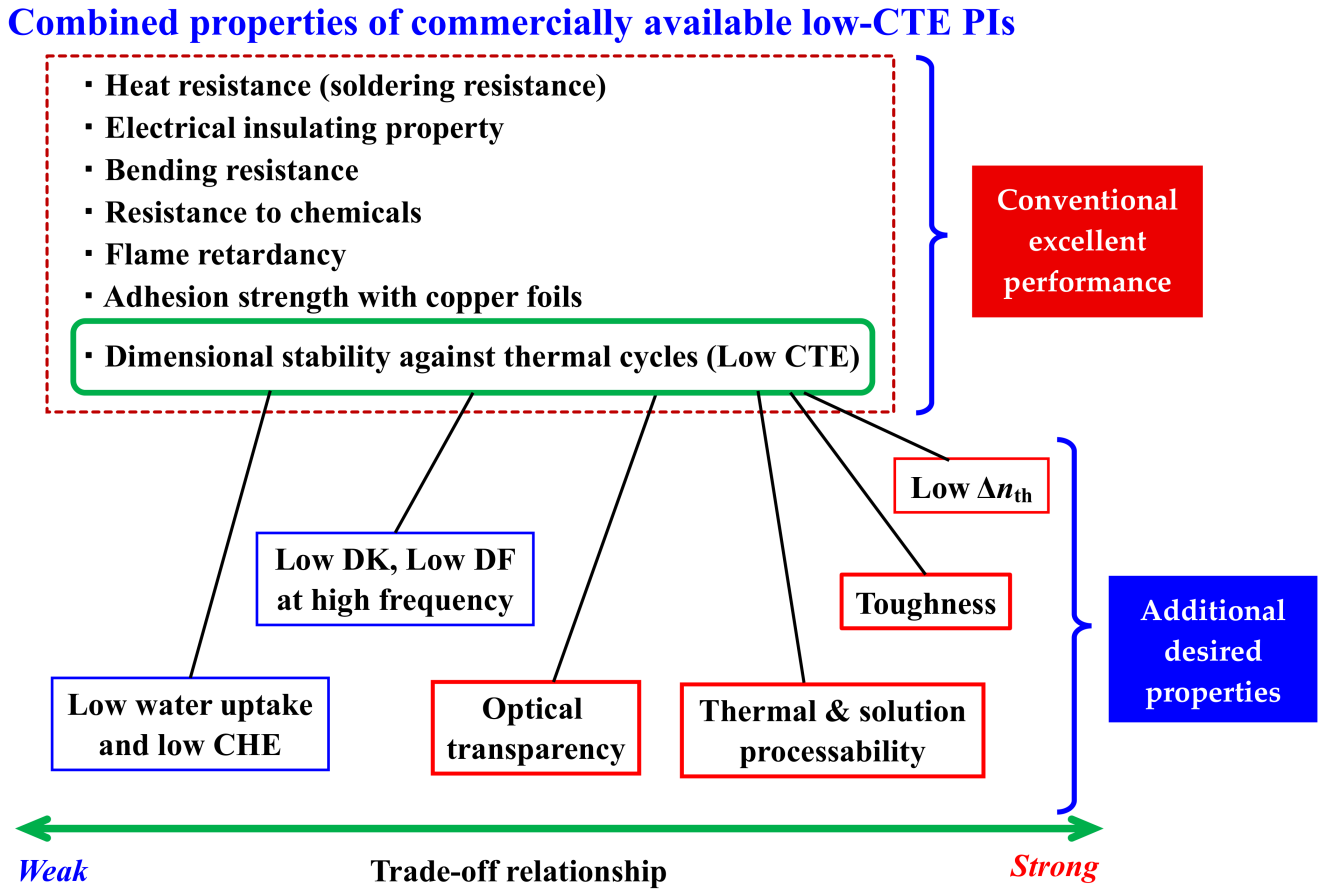
The conventional excellent performance of commercially available low-CTE polyimide films and the additional properties addressed to functionalization. The positions of these additional properties represent the difficulty in simultaneously achieving low CTE and them.

The main required properties of our target materials are summarized in Figure 1. In addition to excellent short-term heat resistance (extremely high *T*_g_), thermal dimensional stability is essential. This can be ensured by reducing the linear coefficients of thermal expansion (CTE) in the film plane (*XY*-direction) in the glassy temperature region (*T* < *T*_g_), (typically CTE < 20 ppm/K). The poor thermal dimensional stability of plastic substrates can lead to serious problems, such as delamination, circuit misalignment, and transparent electrode breakdown, over multiple thermal cycles in device fabrication processes. Linear and rigid main-chain structures are essential to obtain PI films that exhibit significantly reduced CTEs [49,50,51].

In addition, the plastic substrates used in flexible LCDs also require suppressing the thickness-direction birefringence (∆*n*_th_), which significantly deteriorates the image contrast at high viewing angles. However, the simultaneous achievement of low CTE and low ∆*n*_th_ is extremely challenging because there is a trade-off relationship between them; the significant *XY*-direction main-chain alignment (in-plane orientation), which is essential for achieving a low CTE, unavoidably causes an increase in the ∆*n*_th_. Furthermore, as shown in Figure 1, the molecular design strategies targeting low CTE also usually lead to the deterioration of film toughness and solution processability (solubility). Therefore, it is difficult to simultaneously achieve the selected targets to the right in this figure and a low CTE [47,48]. Conventional non-alkali glass substrates do not suffer from this trade-off because they inherently have virtually zero birefringence and a very low CTE, although they are fragile and heavy.

To overcome the above-mentioned trade-off, inorganic fillers (preferably nano-fillers) with an originally low CTE are often dispersed into colorless matrix resins at high contents to reduce the CTE of the resulting resin/filler composites [52,53,54,55]. However, this approach has limitations because excessive filler loading can lead to significant increases in turbidity due to filler aggregation, embrittlement of the resulting films, and filler dropout. Currently, there is no accepted method for simultaneously significantly reducing the CTE and ∆*n*_th_ of neat resins without the help of fillers or mechanical stretching. A copolymerization approach using multiple monomers is often effective in improving the properties of PI films, as illustrated in KAPTON^®^ EN (quaternary copolymer, Toray Kapton Co., Tokyo, Japan) [56]. However, it is extremely difficult to overcome the trade-off between low CTE and low ∆*n*_th_ even by simple copolymerization using multiple existing monomers. Therefore, there is an urgent need for new functional monomers that are effective in simultaneously reducing the CTE and ∆*n*_th_.

**Figure 2:**
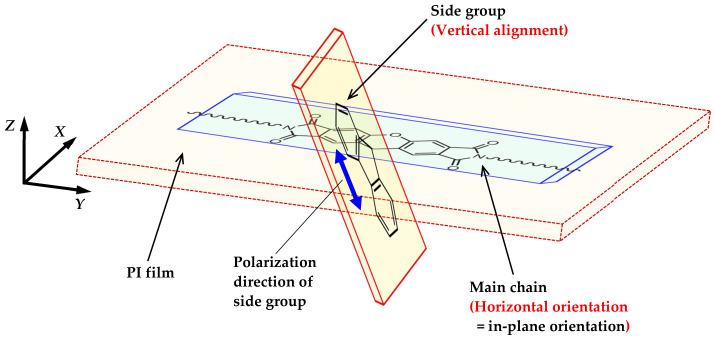
A schematic diagram representing our strategic concept for overcoming the trade-off between low CTE and low Δ*n*_th_ by dividing the roles of the main chain and side group.

In this study, we aimed to overcome the trade-off between low CTE and low ∆*n*_th_ through a combination of self-orientation behavior of specific PIs during solution casting, molecular designs of specially shaped monomers, and optimally selected polymerization processes. Our strategy for the present purpose is illustrated in Figure 2. We relied on the concept of “role-dividing.” That is, the semi-aromatic PI main chains are responsible for ensuring low CTE by their own in-plane orientation (chain alignment in the *XY*-direction), while the highly polarized aromatic side groups play a role in negating the *XY*-direction polarization arising from the main-chain in-plane orientation through the vertical (*Z*-direction) alignment of the side-group long axis.

## 2. Experimental Section

### 2.1. Materials

#### 2.1.1. Monomer Synthesis

A series of modifier monomers used in this study were synthesized using the raw materials listed in Table S1, as described below.

(a) Vertical-alignment-type diamines

**Figure 3:**
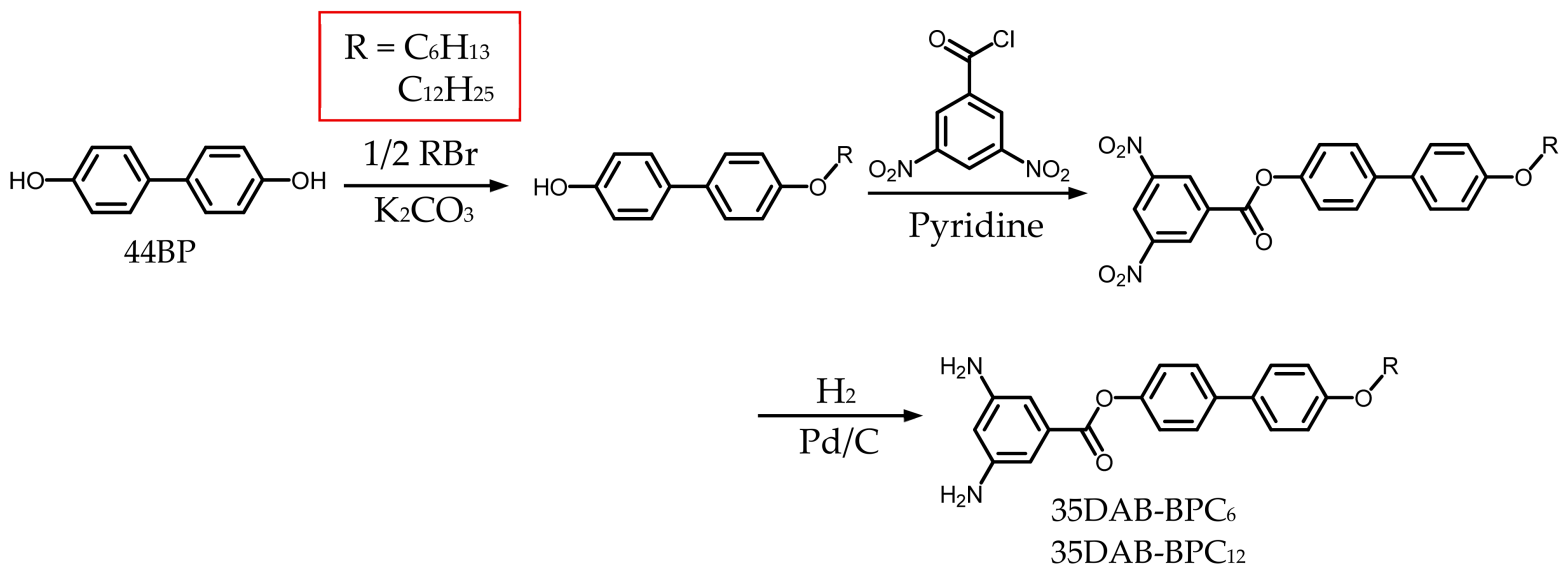
Reaction schemes for the synthesis of vertical-alignment-type diamines (35DAB-BPC_6_ and 35DAB-BPC_12_).

This type of diamine containing an *n*-hexyl group was synthesized according to the reaction scheme (Figure 3) through the following intermediates.

C_6_-BPOH. In a three-neck flask, a large excess of 4,4′-biphenol (44BP, 75 mmol, 13.97 g) was dissolved in anhydrous *N*,*N*-dimethylformamide (DMF, 34.5 mL) in the presence of K_2_CO_3_ (75 mmol, 10.40 g). In another flask, 1-bromohexane (BrC_6_, 15 mmol, 2.48 g) was dissolved in DMF (24.6 mL). To the 44BP solution kept at 100 °C, the BrC_6_ solution was gradually added over 30 min using a dropping funnel, followed by refluxing at 140 °C for 3 h in an N_2_ atmosphere. The reaction mixture was gradually poured into a large quantity of water. The precipitate yielded was collected by filtration, repeatedly washed with water, and dried at 100 °C for 12 h under vacuum. The crude product was dissolved in toluene to remove an undissolved portion (44BP) by filtration. The crude product obtained via solvent removal was recrystallized from a mixed solvent of toluene/cyclohexane (1/1, *v*/*v*), followed by additional recrystallization from *γ*-butyrolactone (GBL)/water (1/1, *v*/*v*), which was effective at purifying. The white plate crystals obtained were dried at 100 °C for 12 h under vacuum (yield: 47%).

**Scheme 1:**
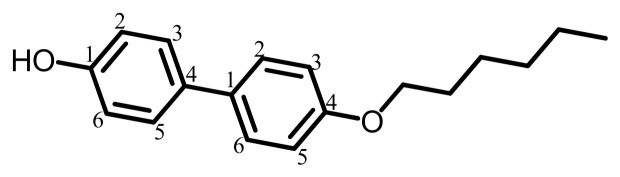
Molecular structure of C_6_-BPOH with numbering.

The analytical data of the product are as follows. Melting point (*T*_m_) determined from a sharp endothermic peak temperature by differential scanning calorimetry (DSC) at a heating rate of 5 °C/min: 159 °C. FT-IR (KBr plate method, cm^−1^): 3384 (O–H, stretching vibration), 2954/2933/2866 (C_aliph_–H), 1609 (biphenylene group), 1503 (1.4-phenylene group), 1250 (C_Ar_–O–C_aliph_). ^1^H-NMR [400 MHz, dimethyl sulfoxide (DMSO)-*d*_6_, *δ*, ppm]: 9.43 [s, 1H (relative integrated intensity: 1.00H), OH], 7.47 [d, 2H (2.06H), *J* = 8.6 Hz, 2,6-protons of the 4-hexanoxyphenyl unit (Ph-OC_6_)], 7.41 [d, 2H (2.11H), *J* = 8.5 Hz, 3,5-protons of the terminal phenol unit (PhOH)], 6.95 [d, 2H (2.04H), *J* = 8.7 Hz, 3,5-protons of Ph–OC_6_], 6.81 [d, 2H (2.01H), *J* = 8.7 Hz, 2,6-protons of PhOH], 3.97 [t, 2H (2.15H), *J* = 6.5 Hz, PhO–CH_2_–CH_2_–], 1.71 [quin, 2H (2.12H), *J* = 7.0 Hz, PhO–CH_2_–CH_2_–CH_2_–], 1.42 [quin (not well-resolved), 2H (2.05H), PhO–(CH_2_)_2_–CH_2_–CH_2_–], 1.33–1.30 [m, 4H (4.03H), PhO–(CH_2_)_3_–CH_2_–CH_2_–CH_3_], 0.88 [t, 3H (3.01H), *J* = 7.0 Hz, PhO–(CH_2_)_5_–CH_3_]. The results confirm that the product is the desired compound (C_6_-BPOH) shown in Scheme 1.

**Scheme 2:**
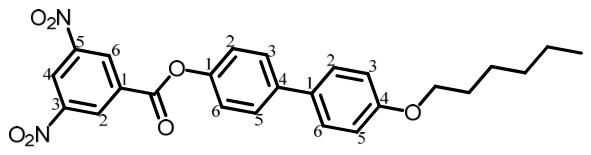
Molecular structure of 35DNB-BPC_6_ with numbering.

35DNB-BPC_6_. In a septum cap-sealed flask, C_6_-BPOH (6.63 mmol, 1.792 g) was dissolved in anhydrous tetrahydrofuran (THF, 3.0 mL) in the presence of anhydrous pyridine (9.95 mmol, 0.80 mL) as an HCl acceptor. In another septum cap-sealed flask, 3,5-dinitrobenzoyl chloride (35DNBC, 6.63 mmol, 1.528 g) was dissolved in THF (4.0 mL) and cooled at 0 °C. To this solution, the C_6_-BPOH was gradually added with a syringe while magnetically stirring. The reaction mixture was diluted with THF (3 mL) and stirred at room temperature for 12 h. The pale-yellowish precipitate yielded was collected by filtration, repeatedly washed with water until the by-produced pyridine–HCl salt was completely removed, and dried at 100 °C for 12 h under vacuum. The analytical data of this product are as follows. *T*_m_ = 137 °C (DSC). FT-IR (KBr plate method, cm^−1^): 2966 (C_aliph_–H), 1738 (ester group, C=O), 1550/1345 (NO_2_), 1498 (1.4-phenylene group), 1207 (C_Ar_–O–C_aliph_). ^1^H-NMR (400 MHz, DMSO-*d*_6_, *δ*, ppm): 9.14–9.10 [m, 3H (3.03H), 2,6- + 4-protons of the 3,5-dinitrobenzoate unit (35DNB)], 7.75 [d, 2H (2.04H), *J* = 8.6 Hz, 3,5-protons of the central COO-Ph unit], 7.64 [d, 2H (1.85H), *J* = 8.5 Hz, 2,6-protons of the PhOC_6_ unit], 7.45 [d, 2H (1.83H), *J* = 8.7 Hz, 2,6-protons of COO-Ph], 7.04 [d, 2H (1.95H), *J* = 8.8 Hz, 3,5-protons of PhOC_6_], 4.02 [t, 2H (2.00H), *J* = 6.5 Hz, PhO–CH_2_–CH_2_–], 1.74 [quin, 2H (1.95H), *J* = 7.0 Hz, PhO–CH_2_–CH_2_–CH_2_–], 1.44–1.32 [m, 6H (5.96H), PhO–(CH_2_)_2_–(CH_2_)_3_–CH_3_], 0.89 [t, 3H (3.04H), *J* = 6.9 Hz, PhO–(CH_2_)_5_–CH_3_]. The results confirm that the product is the desired dinitro compound (35DNB-BPC_6_) shown in Scheme 2.

35DAB-BPC_6_. In a three-neck flask, 35DNB-BPC_6_ (2.24 mmol, 1.041 g) was dissolved in anhydrous DMF (20 mL) in the presence of Pd/C (0.105 g). The reaction mixture was refluxed at 50 °C for 7 h in an H_2_ atmosphere, while monitoring the progress of the catalytic reduction by thin-layer chromatography (TLC). The residual catalyst was removed by hot filtration. The filtrate was gradually poured into a large quantity of water. The precipitate formed was collected by filtration (yield: 83%) and recrystallized from ethanol, followed by vacuum-drying at 100 °C for 12 h.

**Scheme 3:**
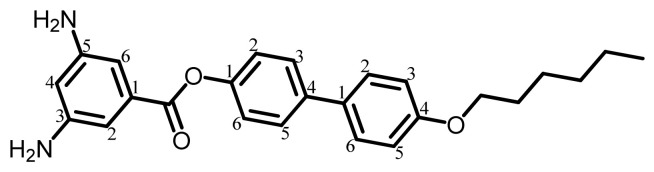
Molecular structure of 35DAB-BPC_6_ with numbering.

The analytical data of the purified product are as follows. *T*_m_ = 175 °C (DSC). FT-IR (KBr plate method, cm^−1^): 3461/3360/3241 (NH_2_, N–H stretching vibration), 2933/2866 (C_aliph_–H), 1724 (ester, C=O), 1607 (biphenylene group), 1498 (1,4-phenylene group), 1203 (C_Ar_–O–C_aliph_). ^1^H-NMR (400 MHz, DMSO-*d*_6_, *δ*, ppm): 7.66 [d, 2H (2.00H), *J* = 8.6 Hz, 3,5-protons of COO-Ph], 7.60 [d, 2H (1.96H), *J* = 8.8 Hz, 2,6-protons of PhOC_6_], 7.24 [d, 2H (1.94H), *J* = 8.6 Hz, 2,6-protons of COO-Ph], 7.02 [d, 2H (2.00H), *J* = 8.8 Hz, 3,5-protons of PhOC_6_], 6.60 [d, 2H (2.00H), *J* = 2.0 Hz, 2,6-protons of the 3,5-diaminobenzoate unit (35DAB)], 6.11 [t, 1H (1.01H), *J* = 2.0 Hz, 4-proton of 35DAB], 5.11 [s, 4H (3.85H), NH_2_], 4.01 [t, 2H (2.04H), *J* = 6.4 Hz, PhO–CH_2_–CH_2_–], 1.73 [quin, 2H (2.02H), *J* = 7.0 Hz, PhO–CH_2_–CH_2_–CH_2_–], 1.43 [quin (not well-resolved), 2H, PhO–(CH_2_)_2_–CH_a2_–CH_2_–], 1.33–1.31 [m, 4H, PhO–(CH_2_)_3_–CH_b2_–CH_c2_–CH_3_], H_a_ (2H) + H_b_ (2H) + H_c_ (2H) = 6H (5.93H), 0.89 [t, 3H (3.07H), *J* = 7.0 Hz, PhO–(CH_2_)_5_–CH_3_]. Elemental analysis: Calcd. (%) for C_25_H_28_O_3_N_2_ (404.51 g/mol): C, 74.23; H, 6.98; N, 6.93. Found: C, 74.21; H, 7.00; N, 6.66. The results confirm that the product is the desired diamine (35DAB-BPC_6_) shown in Scheme 3.

35DAB-BPC_12_. An analogue of 35DAB-BPC_6_, 35DAB-BPC_12_ with a dodecyl group, was synthesized using C_12_H_25_Br in a similar manner. The crude product was recrystallized from a mixed solvent (1,4-dioxane/cyclohexane, 1/2, *v*/*v*).

**Scheme 4:**
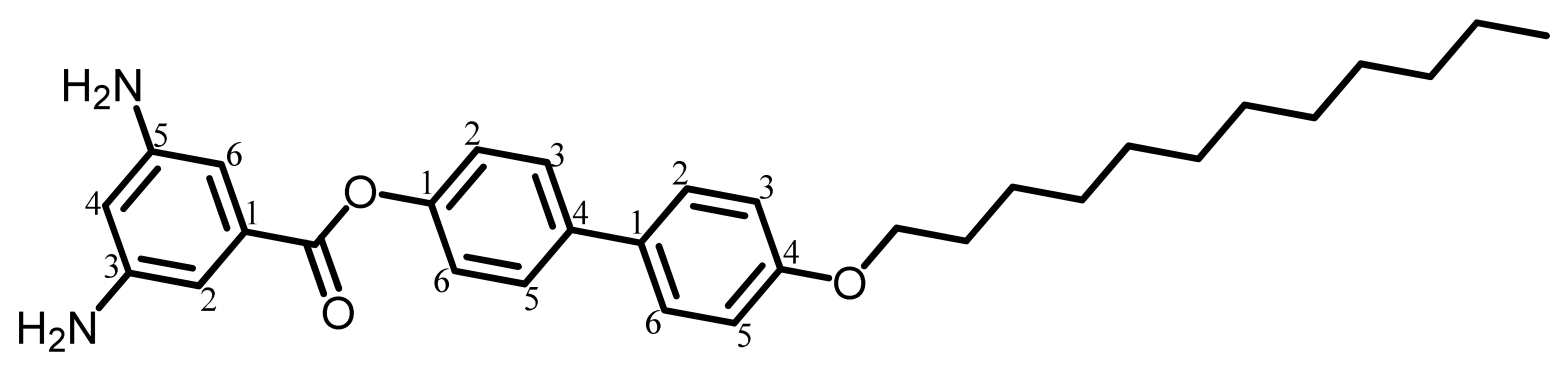
Molecular structure of 35DAB-BPC_12_ with numbering.

The analytical data of the purified product are as follows. *T*_m_ = 170/177 °C (DSC, double endothermic peak). FT-IR (KBr plate method, cm^−1^): 3456/3356/3221 (NH_2_, N–H stretching vibration), 3038 (C_arom_–H). 2923/2852 (C_aliph_–H), 1721 (ester, C=O), 1607 (biphenylene group), 1497 (1,4-phenylene group), 1232 (C_Ar_–O–C_aliph_). ^1^H-NMR (400 MHz, DMSO-*d*_6_, *δ*, ppm): 7.66 [d, 2H (1.98H), *J* = 8.7 Hz, 3,5-protons of the central COO-Ph unit], 7.60 [d, 2H (2.02H), *J* = 8.8 Hz, 2,6-protons of PhOC_6_], 7.24 [d, 2H (1.99H), *J* = 8.7 Hz, 2,6-protons of COO-Ph], 7.01 [d, 2H (2.19H), *J* = 8.8 Hz, 3,5-protons of PhOC_12_], 6.60 [d, 2H (2.04H), *J* = 2.1 Hz, 2,6-protons of 35DAB], 6.11 [t, 1H (1.02H), *J* = 2.0 Hz, 4-proton of 35DAB], 5.09 [s, 4H (3.94H), NH_2_], 4.00 [t, 2H (2.04H), *J* = 6.4 Hz, PhO–CH_2_–CH_2_–], 1.73 [quin (not well-resolved), 2H (2.02H), PhO–CH_2_–CH_2_–CH_2_–], 1.42–1.25 [m, 18H (18.46H), PhO–(CH_2_)_2_–(CH_2_)_9_–CH_3_], 0.85 [t, 3H (3.04H), *J* = 6.8 Hz, PhO–(CH_2_)_11_–CH_3_]. Elemental analysis: Calcd. (%) for C_31_H_40_O_3_N_2_ (488.67 g/mol): C, 76.19; H, 8.25; N, 5.73. Found: C, 76.01; H, 8.46; N, 6.13. The results confirm that the product is 35DAB-BPC_12_, shown in Scheme 4.

(b) *Cardo*-type TCDAs and diamines

*Cardo*-type ester-linked FL-pendant TCDA (*cardo*-TA-BPFL) and diamine (*cardo*-AB-BPFL), and ether-linked diamine (*cardo*-AP-BPFL) were synthesized according to the reaction scheme (Figure S1) and characterized, as described in Documents S1–S3.

(c) *Spiro*-type ester-linked FL-pendant TCDAs and diamines

**Figure 4:**
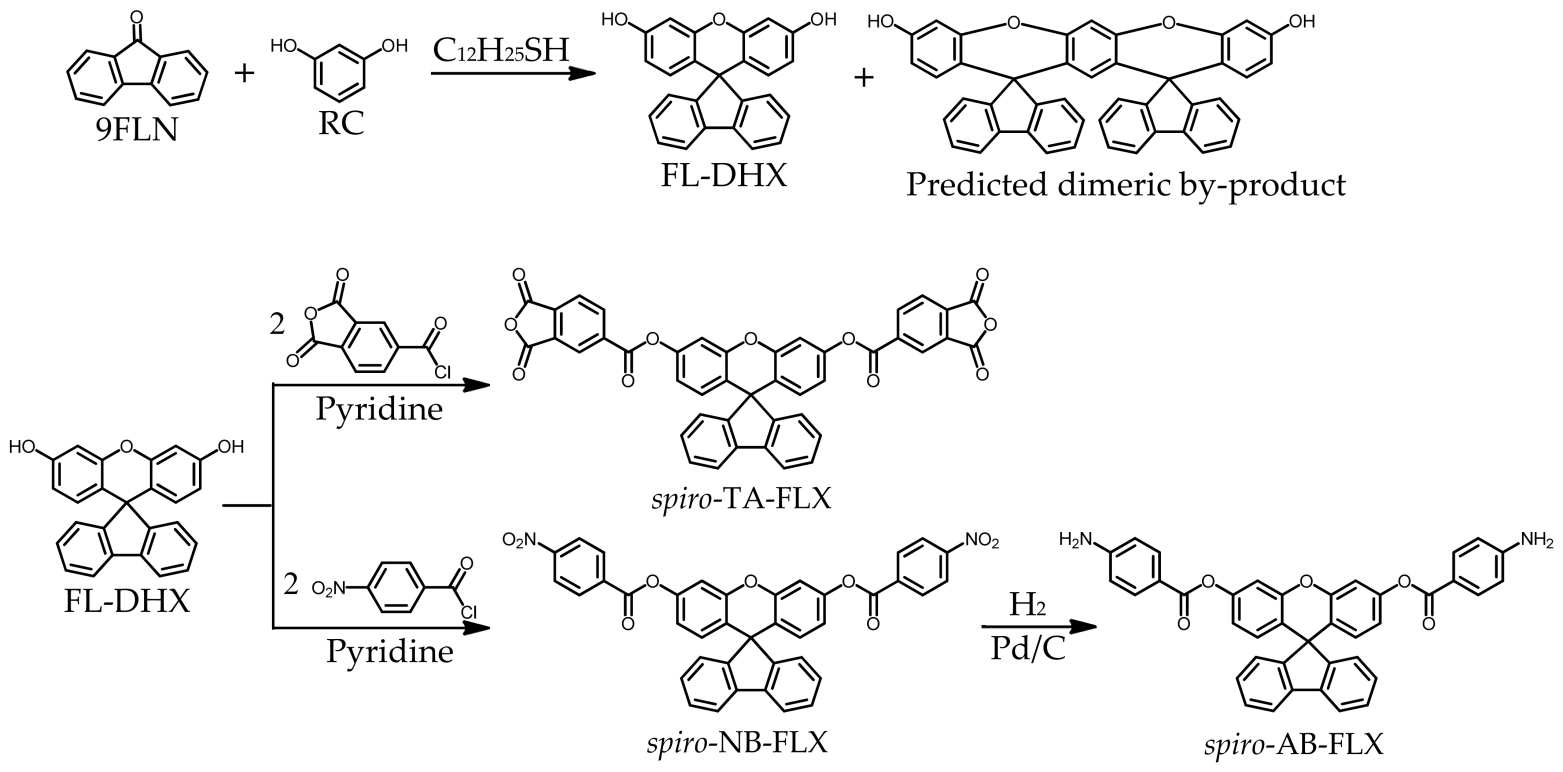
Reaction schemes for the synthesis of *spiro*-type ester-linked TCDA and diamine.

These monomers (*spiro*-TA-FLX and *spiro*-AB-FLX) [38] were synthesized according to the reaction schemes (Figure 4) through the following intermediates.

FL-DHX. Fluorene (FL)-pendant 3,6-dihydroxy xanthene (FL-DHX), was synthesized as follows. In a three-neck 300 mL flask, 9-fluorenone (9FLN, 39.12 mmol), resorcinol (RC, 313.79 mmol), and 1-dodecanethiol (1.56 mmol) were suspended in water (5.75 mL) and maintained at 55–60 °C. To the reaction mixture, conc. HCl (8.47 mL) was added gradually, and the reaction mixture was refluxed at 55–60 °C for 8 h with continuous stirring. After the addition of water (43.1 mL), the reaction mixture was neutralized with an NaOH aqueous solution in an ice bath. The pale-pink precipitate yielded was collected by filtration, washed with water, and dried at 120 °C for 12 h under vacuum. The precipitate was dissolved in 2-propanol, and an undissolved portion, probably including an orange-colored dimeric by-product [38] (Figure 4), was removed by filtration. The crude product obtained by solvent removal of the filtrate was recrystallized from a mixed solvent (2-propanol/water, 4/1, *v*/*v*). The crystals formed were collected by filtration and dried at 100 °C for 12 h under vacuum (total yield: 74%).

**Scheme 5:**
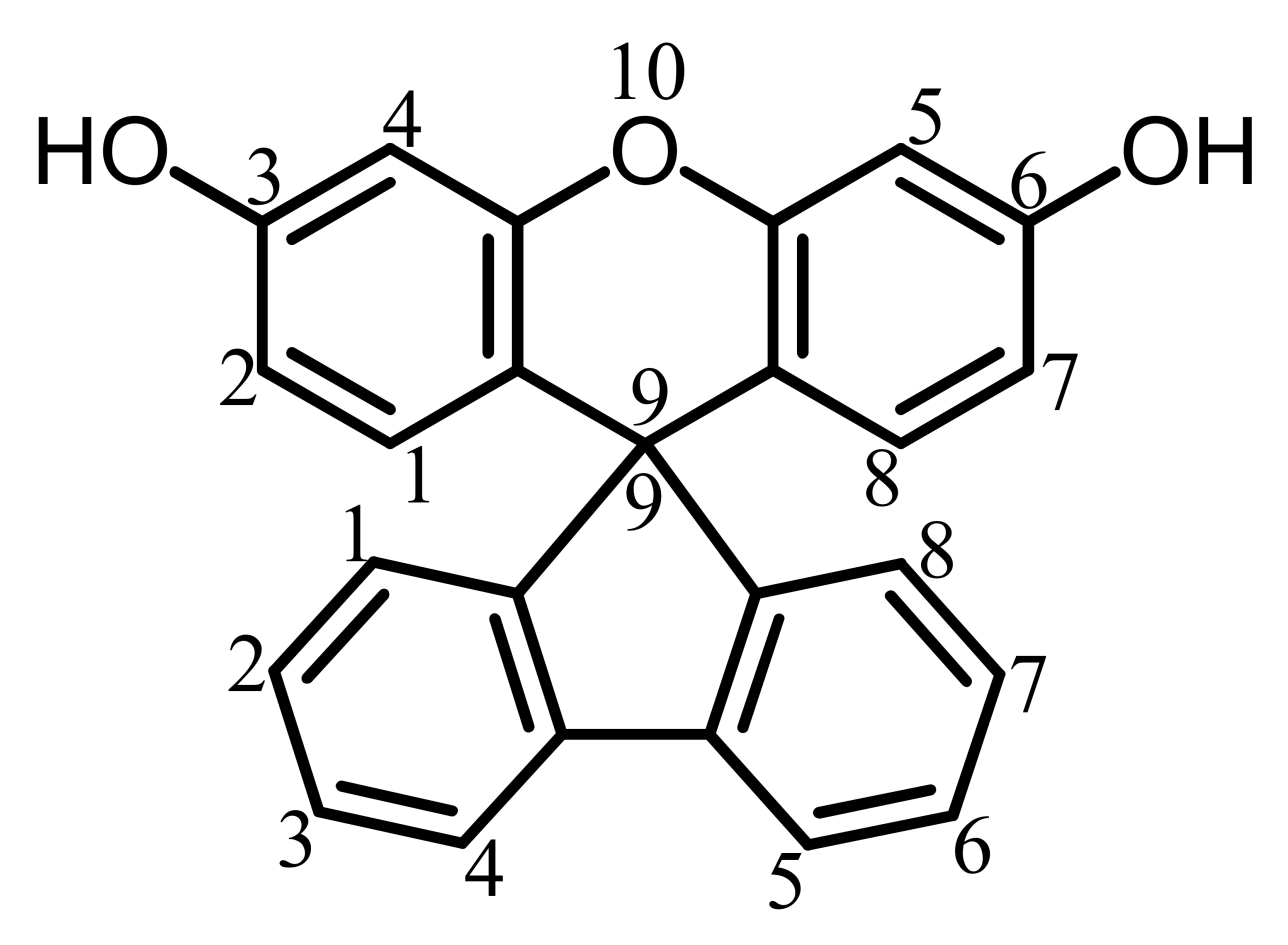
Molecular structure of FL-DHX with numbering.

The analytical data of the purified product are as follows. *T*_m_ = 265 °C (DSC). FT-IR (KBr plate method, cm^−1^): 3505/3405 (O–H), 3061/3036 (C_arom_–H), 1262/1171 (C_arom_–O–C_arom_). ^1^H-NMR [400 MHz, DMSO-*d*_6_, *δ*, ppm]: 9.61 [s, 2H (2.00H), OH], 7.92 [d, 2H (2.04H), *J* = 7.6 Hz, 4,5-protons of FL], 7.37 [t, 2H (2.07H), *J* = 7.4 Hz, 3,6-protons of FL], 7.22 [t, 2H (2.07H), *J* = 7.5 Hz, 2,7-protons of FL], 7.02 [d, 2H (2.03H), *J* = 7.6 Hz, 1,8-protons of FL], 6.58 [d, 2H (2.04H), *J* = 2.4 Hz, 4,5-protons of the xanthene (XAN) unit], 6.26 [dd, 2H (2.01H), *J* = 8.6, 2.4 Hz, 2,7-protons of XAN], 6.03 [d, 2H (2.00H), *J* = 8.6 Hz, 1,8-protons of XAN]. Elemental analysis: Calcd. (%) for C_25_H_16_O_3_ (364.40 g/mol): C, 82.40; H, 4.43. Found: C, 82.47; H, 4.52. These results confirm that the product is the desired bisphenol (FL-DHX) shown in Scheme 5.

*spiro*-TA-FLX. A *spiro*-type ester-linked TCDA was synthesized as follows. In a septum cap-sealed flask, FL-DHX (10.01 mmol) was dissolved in anhydrous THF (47 mL) in the presence of pyridine (4.85 mL, 60 mmol). In another septum cap-sealed flask, trimellitic anhydride chloride (TMAC, 30.09 mmol) was dissolved in anhydrous THF (16.6 mL). To the TMAC solution cooled at 0 °C, the FL-DHX solution was gradually added using a syringe while magnetically stirring, followed by additional stirring at room temperature for 12 h. The precipitate formed was collected by filtration and washed with THF and water. The white precipitate was dried at 160 °C for 12 h under vacuum to ensure ring closure dehydration of a hydrolyzed portion (yield: 67%).

**Scheme 6:**
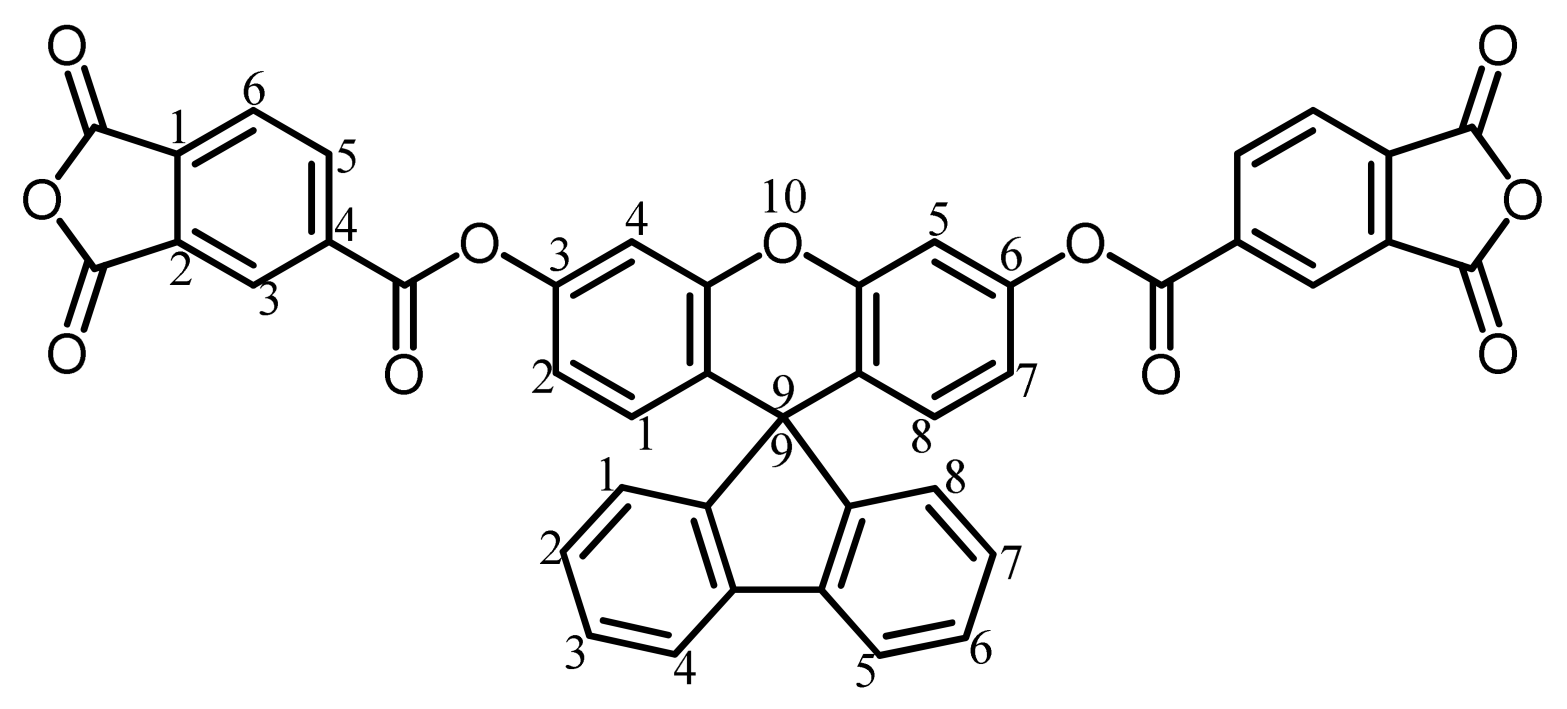
Molecular structure of *spiro*-TA-FLX with numbering.

The analytical data of the product are as follows. *T*_m_ = 331 °C (DSC). FT-IR (KBr plate method, cm^−1^): 3066 (C_arom_–H), 1856/1782 (acid anhydride, C=O), 1739 (ester, C=O), 1493 (phenyl group), 1224/1168 (C_arom_–O–C_arom_), and the absence of the absorption bands at 3400–3500 cm^−1^ (O–H) from the unreacted FL-DHX and ~2600 cm^−1^ (hydrogen-bonded carboxylic acid, O–H) from the possible by-product due to the hydrolyzed TMAC. ^1^H-NMR [400 MHz, DMSO-*d*_6_, *δ*, ppm]: 8.62–8.58 [m, 4H (4.01H), 3,3′- + 5,5′-protons of the phthalic anhydride (PAn) unit], 8.27 [d, 2H (2.02H), *J* = 7.8 Hz, 6,6′-protons of PAn], 8.04 [d, 2H (2.00H), *J* = 7.8 Hz, 4,5-protons of the fluorene (FL) unit], 7.49–7.46 [m, 4H (4.01H), 3,6-protons of FL + 4,5-protons of XAN], 7.32 [t, 2H (2.02H), *J* = 7.6 Hz, 2,7-protons of FL], 7.21 [d, 2H (2.02H), *J* = 7.6 Hz, 1,8-protons of FL], 6.94 [d, 2H (2.00H), *J* = 8.6 Hz, 2,7-protons of XAN], 6.40 [d, 2H (2.03H), *J* = 8.6 Hz, 1,8-protons of XAN]. The observed high-magnetic field shift of the 1,8-protons of the FL-pendant XAN (cf. *δ* = 7.25 ppm for the 1,8-protons of non-substituted xanthene in DMSO-*d*_6_ at 400 MHz [57]) is likely attributed to a shielding effect based on the FL group, which is arranged through the *sp*^3^ carbon atom perpendicularly to the XAN molecular plane. A similar effect was observed in the analogues synthesized in this study, as shown later. Elemental analysis: Calcd. (%) for C_43_H_20_O_11_ (712.63 g/mol): C, 72.47; H, 2.83. Found: C, 72.61; H, 2.96. These results confirm that the product is the desired TCDA (*spiro*-TA-FLX) shown in Scheme 6.

*spiro*-NB-FLX. In a septum cap-sealed flask, 4-nitrobenzoyl chloride (4NBC, 30.0 mmol) was dissolved in anhydrous THF (9.4 mL). In another septum cap-sealed flask, FL-DHX (10.02 mmol) was dissolved in THF (47.1 mL) in the presence of pyridine (60 mmol). To the 4NBC solution cooled at 0 °C, the FL-DHX solution was gradually added using a syringe while magnetically stirring, followed by additional stirring at room temperature for 12 h. The pale-yellowish precipitate formed was collected by filtration, washed with a small quantity of THF, a large quantity of water, and methanol, and dried at 120 °C for 12 h under vacuum (yield: 68%).

**Scheme 7:**
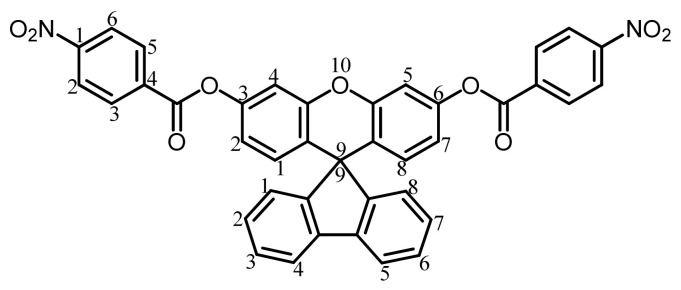
Molecular structure of *spiro*-NB-FLX with numbering.

The analytical data of the product are as follows. *T*_m_ = 292 °C (DSC). FT-IR (KBr plate method, cm^−1^): 3075 (C_arom_–H), 1742 (ester, C=O), 1522/1347 (NO_2_), 1486 (1,4-phenylene group), 1239/1152 (C_arom_–O–C_arom_), and the absence of the absorption bands at 3400–3500 (O–H) from the unreacted FL-DHX. ^1^H-NMR (400 MHz, DMSO-*d*_6_, *δ*, ppm): 8.41 [d, 4H (4.04H), *J* = 9.0 Hz, 2,2′,6,6′-protons of the nitrobenzene (NB) group], 8.34 [d, 4H (4.03H), *J* = 9.0 Hz, 3,3′,5,5′-protons of NB], 8.04 [d, 2H (2.01H), *J* = 7.6 Hz, 4,5-protons of FL], 7.47 [t, 2H (2.04H), *J* = 7.5 Hz, 3,6-protons of FL], 7.42 [d, 2H (2.03H), *J* = 2.4 Hz, 4,5-protons of XAN], 7.31 [t, 2H (2.04H), *J* = 7.5 Hz, 2,7-protons of FL], 7.20 [d, 2H (2.00H), *J* = 7.5 Hz, 1,8-protons of FL], 6.89 [dd, 2H (2.05H), *J* = 8.6, 2.4 Hz, 2,7-protons of XAN], 6.39 [d, 2H (2.00H), *J* = 8.6 Hz, 1,8-protons of XAN]. These results confirm that the product is the desired dinitro compound (*spiro*-NB-FLX) shown in Scheme 7.

*spiro*-AB-FLX. In a three-neck flask, *spiro*-NB-FLX (5.80 mmol) was dissolved in anhydrous DMF (100 mL) in the presence of Pd/C (0.424 g). The reaction mixture was refluxed at 100 °C for 4 h in a hydrogen atmosphere and cooled to room temperature. After the catalyst residue was filtered out, the filtrate was concentrated using an evaporator, and subsequently, water (200 mL) was added to the flask. The white precipitate formed was collected by filtration, washed with water and methanol, and dried at 120 °C for 12 h under vacuum (yield: 81%).

**Scheme 8:**
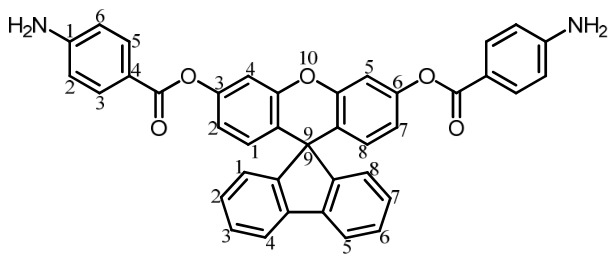
Molecular structure of *spiro*-AB-FLX with numbering.

The analytical data of the product are as follows. *T*_m_ = 274 °C (DSC). FT-IR (KBr plate method, cm^−1^): 3480/3375/3218 (NH_2_, N–H stretching), 3066 (C_arom_–H), 1708 (ester, C=O), 1628 (NH_2_, deformation + biphenyl group in FL), 1517/1489 (phenyl group), 1237/1172 (C_arom_–O–C_arom_). ^1^H-NMR (400 MHz, DMSO-*d*_6_, *δ*, ppm): 8.01 [d, 2H (2.01H), *J* = 7.5 Hz, 4,5-protons of FL], 7.77 [d, 4H (4.05H), *J* = 8.7 Hz, 3,3′,5,5′-protons of the aniline (AN) group], 7.46 [t, 2H (2.03H), *J* = 7.4 Hz, 3,6-protons of FL], 7.31 [t, 2H (2.04H), *J* = 7.5 Hz, 2,7-protons of FL], 7.20 [d, 2H (2.02H), *J* = 2.3 Hz, 4,5-protons of XAN], 7.17 [d, 2H (2.02H), *J* = 7.6 Hz, 1,8-protons of FL], 6.74 [dd, 2H (2.02H), *J* = 8.5, 2.3 Hz, 2,7-protons of XAN], 6.62 [d, 4H (4.04H), *J* = 8.7 Hz, 2,2′,6,6′-protons of AN], 6.32 [d, 2H (2.03H), *J* = 8.6 Hz, 1,8-protons of XAN], 6.19 [s, 4H (4.00H), NH_2_]. Elemental analysis: Calcd. (%) for C_39_H_26_O_5_N_2_ (602.65 g/mol): C, 77.73; H, 4.35; N, 4.65. Found: C, 77.52; H, 4.50; N, 4.67. These results confirm that the product is the desired diamine (*spiro*-AB-FLX) shown in Scheme 8.

(d) *Spiro*-type ether-linked FL-pendant diamines

**Figure 5:**
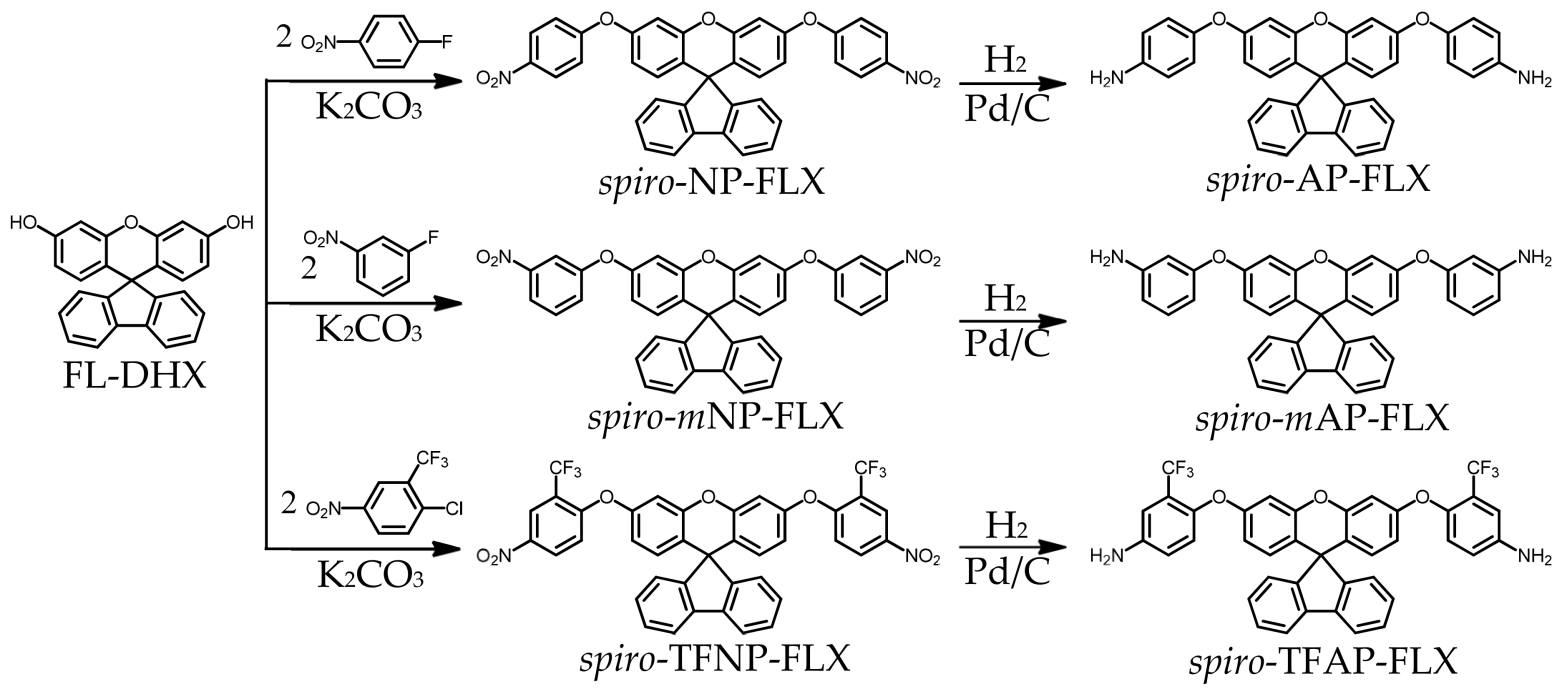
Reaction schemes for the synthesis of *spiro*-type ether-linked diamines.

This type of diamine was synthesized according to the reaction schemes (Figure 5) through the following intermediates.

*spiro*-NP-FLX. In a three-neck flask, FL-DHX (12.66 mmol) and 4-fluoronitrobenzene (4FNB, 33.92 mmol) were dissolved in anhydrous *N*,*N*-dimethylacetamide (DMAc, 30 mL) in the presence of K_2_CO_3_ (29.60 mmol), and the reaction mixture was refluxed at 160 °C for 4 h in a nitrogen atmosphere. The reaction mixture was gradually poured into a large quantity of water. The pale-yellowish precipitate formed was collected by filtration, washed with water, and dried at 120 °C for 12 h under vacuum (yield: 98%).

**Scheme 9:**
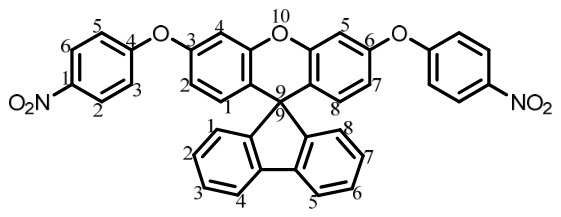
Molecular structure of *spiro*-NP-FLX with numbering.

The analytical data of the product are as follows. *T*_m_ = 190 °C (DSC). FT-IR (KBr plate method, cm^−1^): 3070 (C_arom_–H), 1522/1343 (NO_2_), 1483 (1,4-phenylene group), 1230/1150 (C_arom_–O–C_arom_), and the absence of the absorption bands at 3400–3500 (O–H) from the unreacted FL-DHX. ^1^H-NMR (400 MHz, DMSO-*d*_6_, *δ*, ppm): 8.26 [d, 4H (4.06H), *J* = 9.3 Hz, 2,2′,6,6′-protons of NB], 8.02 [d, 2H (2.00H), *J* = 7.6 Hz, 4,5-protons of FL], 7.46 [t, 2H (1.99H), *J* = 7.5 Hz, 3,6-protons of FL], 7.32 [t, 2H (1.90H), *J* = 7.6 Hz, 2,7-protons of FL], 7.21–7.17 [m, 6H (6.05H), 3,3′,5,5′-protons of NB + 1,8-protons of FL], 7.13 [d, 2H (2.05H), *J* = 2.5 Hz, 4,5-protons of XAN], 6.72 [dd, 2H (2.05H), *J* = 8.6, 2.5 Hz, 2,7-protons of XAN], 6.37 [d, 2H (2.04H), *J* = 8.6 Hz, 1,8-protons of XAN]. These results confirm that the product is the desired dinitro compound (*spiro*-NP-FLX) shown in Scheme 9.

*spiro*-AP-FLX. In a three-neck flask, *spiro*-NP-FLX (12.4 mmol) was dissolved in 1,4-dioxane (75 mL) in the presence of Pd/C (0.746 g), and the reaction mixture was refluxed at 80 °C for 8 h in a hydrogen atmosphere. After the catalyst residue was filtered out, the filtrate was gradually poured into a large quantity of water. The white precipitate formed was collected by filtration, washed with water and methanol, and recrystallized from toluene (yield: 42%).

**Scheme 10:**
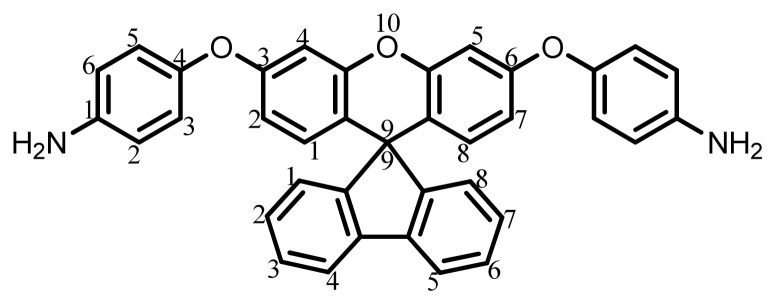
Molecular structure of *spiro*-AP-FLX with numbering.

The analytical data of the purified product are as follows. *T*_m_ = 233 °C (DSC). FT-IR (KBr plate method, cm^−1^): 3431/3353/3216 (NH_2_, N–H stretching), 3044/3011 (C_arom_–H), 1609 (NH_2_, deformation + biphenyl group in FL), 1509/1489 (1,4-phenylene group), 1210/1168 (C_arom_–O–C_arom_). ^1^H-NMR (400 MHz, DMSO-*d*_6_, *δ*, ppm): 7.95 [d, 2H (2.00H), *J* = 7.5 Hz, 4,5-protons of FL], 7.40 [t, 2H (2.04H), *J* = 7.4 Hz, 3,6-protons of FL], 7.24 [t, 2H (1.99H), *J* = 7.5 Hz, 2,7-protons of FL], 7.07 [d, 2H (1.91H), *J* = 7.5 Hz, 1,8-protons of FL], 6.77 [d, 4H (3.94H), *J* = 6.7 Hz, 3,3′,5,5′-protons of AN], 6.61–6.56 [m, 6H (5.87H), 4,5-protons of XAN + 2,2′,6,6′-protons of AN], 6.38 [dd, 2H (1.96H), *J* = 8.6, 2.5 Hz, 2,7-protons of XAN], 6.15 [d, 2H (1.99H), *J* = 8.7 Hz, 1,8-protons of XAN], 5.02 [s, 4H (3.75H), NH_2_]. Elemental analysis: Calcd. (%) for C_37_H_26_O_3_N_2_ (546.63 g/mol): C, 81.30; H, 4.79; N, 5.12. Found: C, 81.67; H, 4.92; N, 5.15. These results confirm that the product is the desired diamine (*spiro*-AP-FLX) shown in Scheme 10.

An isomer of *spiro*-AP-FLX, *spiro*-*m*AP-FLX, was synthesized using 3-fluoronitrobenzene (3FNB) instead of 4FNB in a similar manner (yield: 88%) through the following intermediate.

**Scheme 11:**
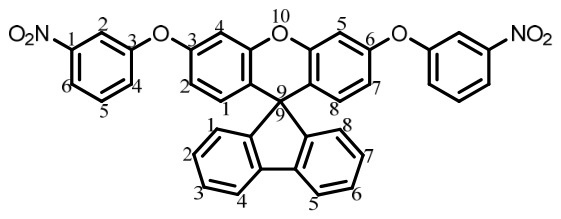
Molecular structure of *spiro*-*m*NP-FLX with numbering.

*spiro*-*m*NP-FLX. The analytical data of the product are as follows. *T*_m_ = 214 °C (DSC). FT-IR (KBr plate method, cm^−1^): 3070 (C_arom_–H), 1525/1349 (NO_2_), 1492 (phenyl group), 1225/1161 (C_arom_–O–C_arom_), and the absence of the absorption bands at 3400–3500 (O–H) from the unreacted FL-DHX. ^1^H-NMR (400 MHz, DMSO-*d*_6_, *δ*, ppm): 8.03–7.99 [m, 4H (4.18H), 6,6′-protons of NB + 4,5-protons of FL], 7.79 [t, 2H (2.00H), *J* = 2.2 Hz, 2,2′-protons of NB], 7.68 [t, 2H (1.99H), *J* = 8.2 Hz, 5,5′-protons of NB], 7.52 [dd, 2H (1.92H), *J* = 8.2, 2.4 Hz, 4,4′-protons of NB], 7.44 [t, 2H (1.97H), *J* = 7.4 Hz, 3,6-protons of FL], 7.30 [t, 2H (2.07H), *J* = 7.5 Hz, 2,7-protons of FL], 7.16 [d, 2H (2.04H), *J* = 7.6 Hz, 1,8-protons of FL], 7.00 [d, 2H (2.00H), *J* = 2.5 Hz, 4,5-protons of XAN], 6.64 [dd, 2H (2.10H), *J* = 8.7, 2.5 Hz, 2,7-protons of XAN], 6.33 [d, 2H (2.08H), *J* = 8.7 Hz, 1,8-protons of XAN]. These results confirm that the product is *spiro*-*m*NP-FLX, shown in Scheme 11.

**Scheme 12:**
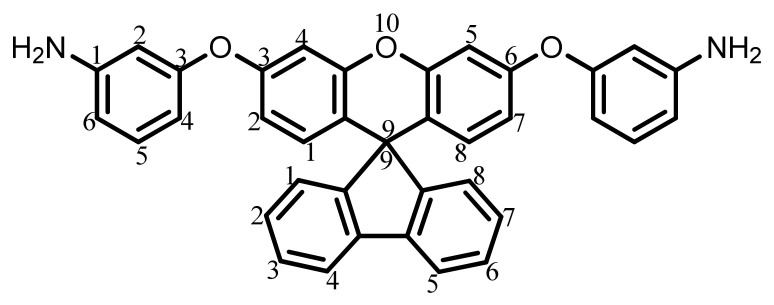
Molecular structure of *spiro*-*m*AP-FLX with numbering.

*spiro*-*m*AP-FLX. The catalytic reduction of the nitro groups in *spiro*-*m*NP-FLX was conducted in a similar manner to that described in the reduction of *spiro*-NP-FLX (yield: 91%). The analytical data of the product are as follows. *T*_m_ = 232 °C (DSC, broad). FT-IR (KBr plate method, cm^−1^): 3466/3380/3217 (NH_2_, N–H stretching), 3061 (C_arom_–H), 1604 (NH_2_, deformation + biphenyl group in FL), 1486 (phenyl group), 1252/1177 (C_arom_–O–C_arom_). ^1^H-NMR (400 MHz, DMSO-*d*_6_, *δ*, ppm): 7.97 [d, 2H (2.00H), *J* = 7.6 Hz, 4,5-protons of FL], 7.42 [t, 2H (2.07H), *J* = 7.5 Hz, 3,6-protons of FL], 7.28 [t, 2H (2.06H), *J* = 7.5 Hz, 2,7-protons of FL], 7.11 [d, 2H (1.98H), *J* = 7.5 Hz, 1,8-protons of FL], 7.00 [t, 2H (1.94H), *J* = 8.0 Hz, 5,5′-protons of AN], 6.78 [d, 2H (1.97H), *J* = 2.4 Hz, 4,5-protons of XAN], 6.50 [dd, 2H (1.99H), *J* = 8.7, 2.5 Hz, 2,7-protons of XAN], 6.35 [dd, 2H (1.92H), *J* = 8.0, 1.3 Hz, 4,4′-protons of AN], 6.23–6.21 [m, 4H (4.01H), 1,8-protons of XAN + 2,2′-protons of AN], 6.15 [dd, 2H (2.08H), *J* = 7.9, 1.7 Hz, 6,6′-protons of AN], 5.26 [s, 4H (3.92H), NH_2_]. These results confirm that the product is *spiro*-*m*AP-FLX, shown in Scheme 12.

CF_3_-substituted *spiro*-AP-FLX (*spiro*-TFAP-FLX) was synthesized using 1-chloro-4-nitro-2-(trifluoromethyl)benzene (1C4NTFB) instead of 4FNB in a similar manner through the following intermediate (yield: 92%).

**Scheme 13:**
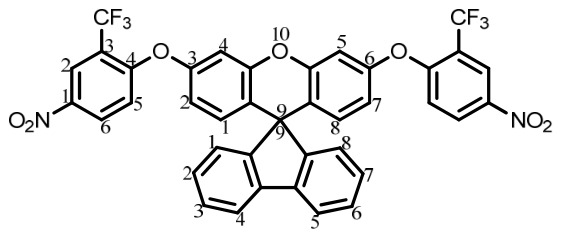
Molecular structure of *spiro*-TFNP-FLX with numbering.

*spiro*-TFNP-FLX. The analytical data of the product are as follows. *T*_m_: not observed on DSC. FT-IR (KBr plate method, cm^−1^): 3089 (C_arom_–H), 1593 (biphenyl group in FL), 1534/1353 (NO_2_), 1481 (1,4-phenylene group), 1268 (C_arom_–O–C_arom_), 1147 (CF_3_, C–F), and the absence of the absorption bands at 3400–3500 (O–H) from the unreacted FL-DHX. ^1^H-NMR (400 MHz, DMSO-*d*_6_, *δ*, ppm): 8.51 [d, 2H, *J* = 2.7 Hz, 2,2′-protons of NB (H_a_)], 8.48 [dd, 2H, *J* = 9.1, 2.9 Hz, 6,6′-protons of NB (H_b_)], H_a_ (2H) + H_b_ (2H) = 4H (4.02H), 8.03 [d, 2H (2.00H), *J* = 7.6 Hz, 4,5-protons of FL], 7.47 [t, 2H (1.97H), *J* = 7.5 Hz, 3,6-protons of FL], 7.33 [t, 2H (2.05H), *J* = 7.1 Hz, 2,7-protons of FL], 7.25 [d, 2H, *J* = 9.2 Hz, 5,5′-protons of NB (H_c_)], 7.22–7.20 [m, 4H, 1,8-protons of FL (H_d_) + 4,5-protons of XAN (H_e_)], H_c_ (2H) + H_d_ (2H) + H_e_ (2H) = 6H (6.07H), 6.77 [dd, 2H (2.05H), *J* = 8.6, 2.5 Hz, 2,7-protons of XAN], 6.41 [d, 2H (2.10H), *J* = 8.6 Hz, 1,8-protons of XAN]. These results confirm that the product is the desired dinitro compound (*spiro*-TFNP-FLX) shown in Scheme 13.

*spiro*-TFAP-FLX. The catalytic reduction of the nitro groups in *spiro*-TFNP-FLX was conducted in a similar manner to that described in the reduction of *spiro*-NP-FLX (yield: 77%). The crude product was purified by recrystallization from 2-propanol/*n*-hexane (1/4, *v*/*v*) (yield: 51%) and subsequent washing with methanol.

**Scheme 14:**
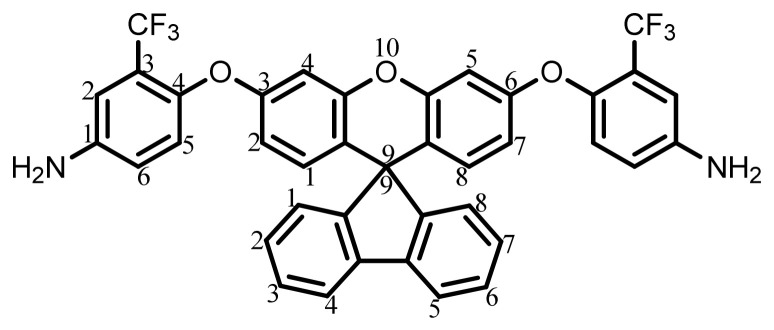
Molecular structure of *spiro*-TFAP-FLX with numbering.

The analytical data of the purified product are as follows. *T*_m_ = 222 °C (DSC). FT-IR (KBr plate method, cm^−1^): 3466/3387 (NH_2_, N–H stretching), 3063 (C_arom_–H), 1631 (NH_2_, deformation), 1606 (biphenyl group in FL), 1486 (1,4-phenyl group), 1338/1149 (CF_3_, C–F), 1221 (C_arom_–O–C_arom_). ^1^H-NMR (400 MHz, DMSO-*d*_6_, *δ*, ppm): 7.96 [d, 2H (2.00H), *J* = 7.5 Hz, 4,5-protons of FL], 7.41 [t, 2H (1.98H), *J* = 7.5 Hz, 3,6-protons of FL], 7.26 [t, 2H (1.98H), *J* = 7.5 Hz, 2,7-protons of FL], 7.09 [d, 2H (1.94H), *J* = 7.5 Hz, 1,8-protons of FL], 6.94–6.90 [m, 4H (4.02H), 2,2′- + 5,5′-protons of AN], 6.81 [dd (not well-resolved), 2H (1.94H), 6,6′-protons of AN], 6.65 [d, 2H (1.95H), *J* = 2.6 Hz, 4,5-protons of XAN], 6.42 [dd, 2H (1.93H), *J* = 8.7, 2.5 Hz, 2,7-protons of XAN], 6.20 [d, 2H (1.98H), *J* = 8.6 Hz, 1,8-protons of XAN], 5.50 [s, 4H (3.93H), NH_2_]. Elemental analysis: Calcd. (%) for C_39_H_24_O_3_N_2_F_6_ (682.62 g/mol): C, 68.62; H, 3.54; N, 4.10. Found: C, 68.93; H, 3.71; N, 4.10. These results confirm that the product is the desired diamine (*spiro*-TFAP-FLX) shown in Scheme 14.

(e) *Spiro*-type alkoxy-substituted XAN-pendant 2,7-diaminofluorenes

**Figure 6:**
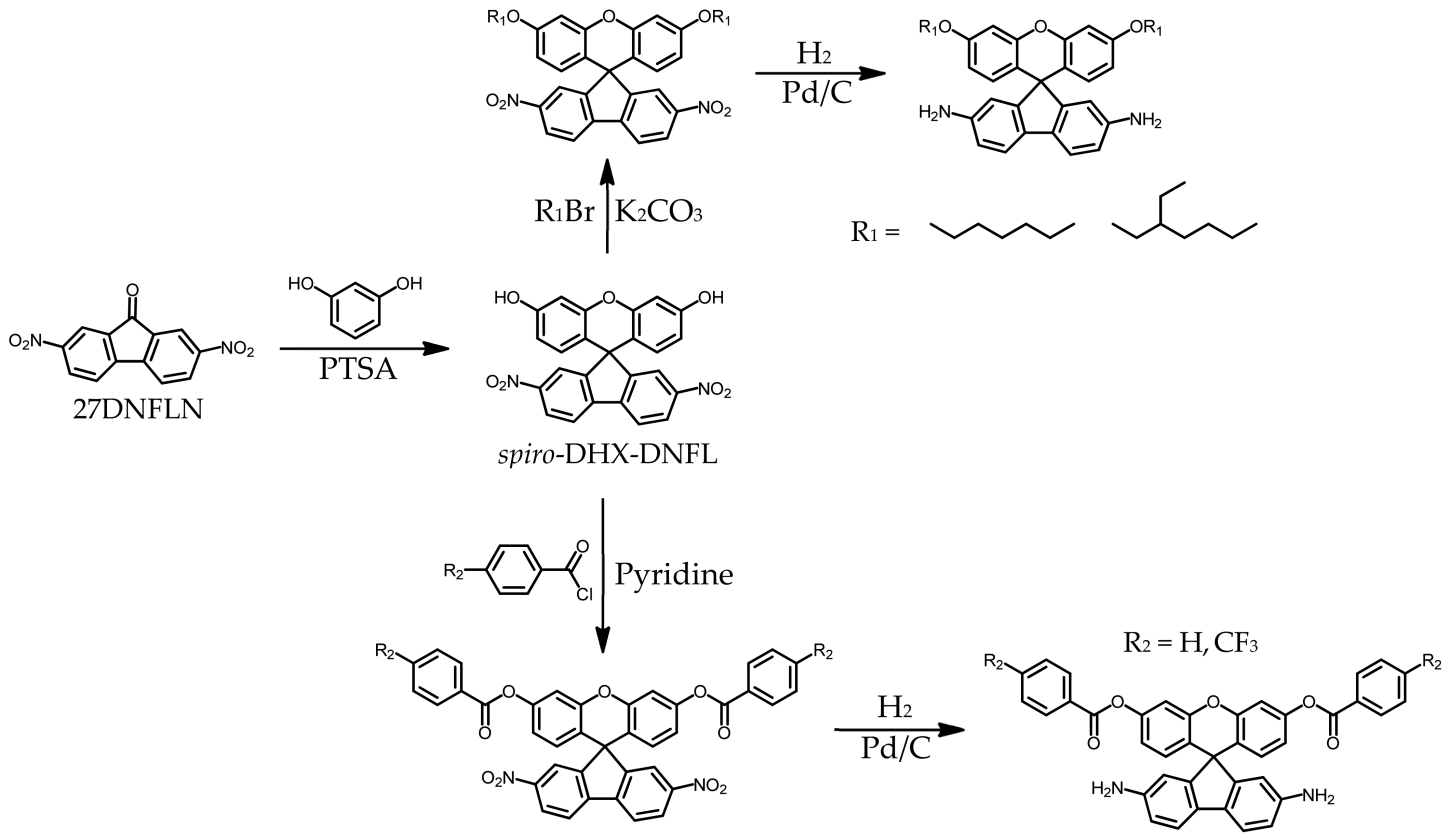
Reaction schemes for the synthesis of *spiro*-type XAN-pendant 2,7-diaminofluorenes.

This type of diamine containing different alkoxy groups was synthesized according to the reaction scheme (Figure 6) through the following intermediates.

**Figure 7:**
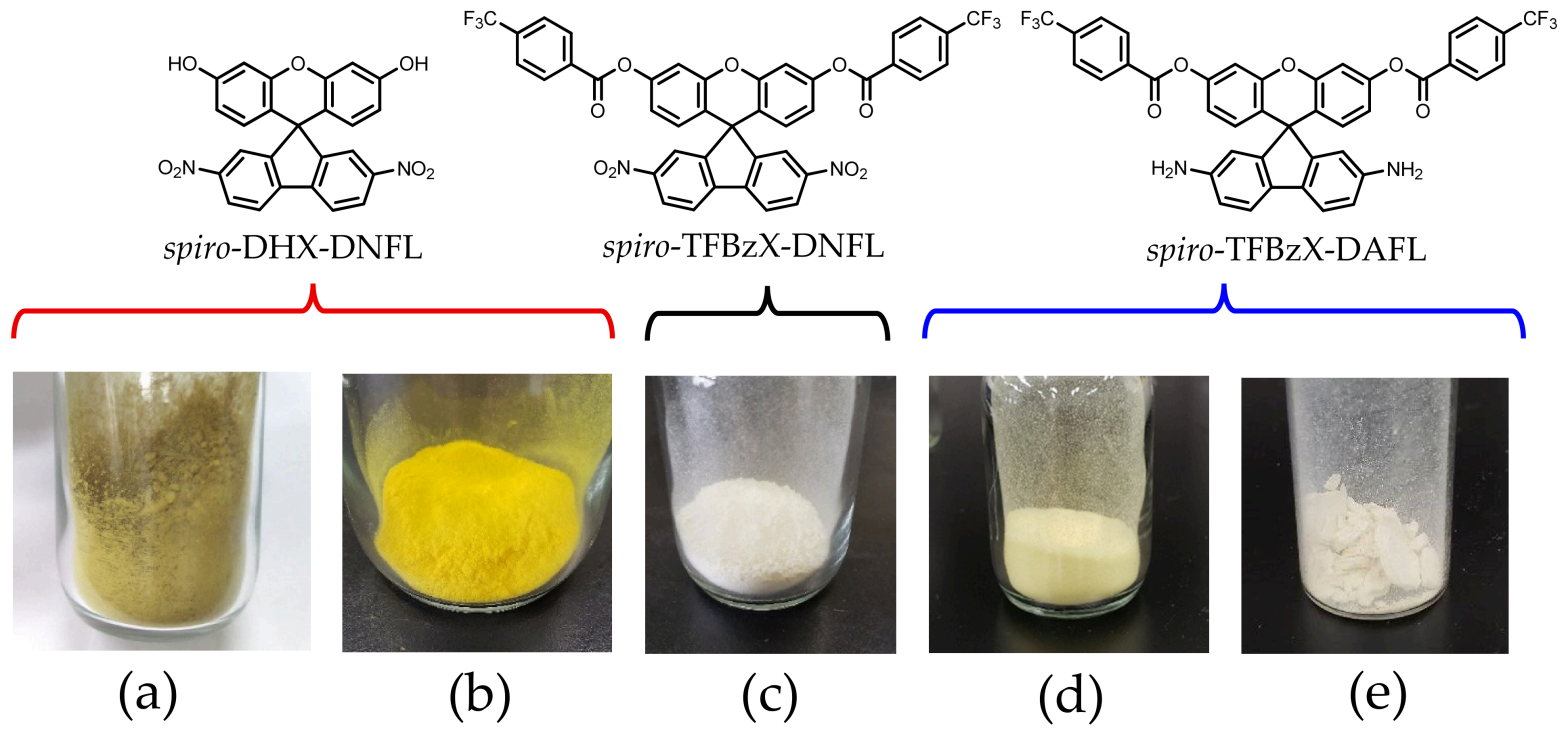
Appearance of the intermediates and purified/decolorized product of *spiro*-TFBzX-DAFL: (**a**) crude *spiro*-DHX-DNFL (dinitro bisphenol), (**b**) *spiro*-DHX-DNFL purified by dry-charge column chromatography, (**c**) *spiro*-TFBzX-DNFL (dinitro compound), (**d**) crude *spiro*-TFBzX-DAFL (diamine), and (**e**) decolorized *spiro*-TFBzX-DAFL after recrystallization.

*spiro*-DHX-DNFL. 2,7-Dinitro-9-fluorenenone (27DNFLN, 20.03 mmol), resorcinol (RC, 120.17 mmol), *p*-toluenesulfonic acid monohydrate (PTSA, 2.06 mmol), and toluene (150 mL) were charged in a three-neck 300 mL flask, and the suspension was refluxed at 110 °C for 6.5 h in an N_2_ atmosphere, as reported in the literature [58]. The reaction mixture was gradually poured into a large quantity of water with vigorous stirring. The ochre-colored or yellowish-brown precipitate yielded, which probably contains an intensely colored dimeric product [58] similar to that depicted at the top of Figure 4, was repeatedly washed with water and dried at 120 °C for 12 h under vacuum (yield: 85%, Figure 7a). The crude product was purified by dry-charge column chromatography using a silica gel (Fujifilm Wako Pure Chemical Corp., Osaka, Japan, Wakogel C-300) and an eluent (ethyl acetate/*n*-hexane, 1/1, *v*/*v*). The stationary phase (sample-adsorbed silica gel) was prepared by stirring the crude product (5.03 g) and Wakogel C-300 (15 g) in acetone (63 mL) and subsequently removing the solvent using an evaporator. The stationary phase was charged at the top of the silica gel column. The main fraction was selectively collected, and the solvent was removed using an evaporator. The resulting yellow precipitate was washed with methanol and dried at 100 °C for 12 h under vacuum (purification yield: 45%, Figure 7b).

**Scheme 15:**
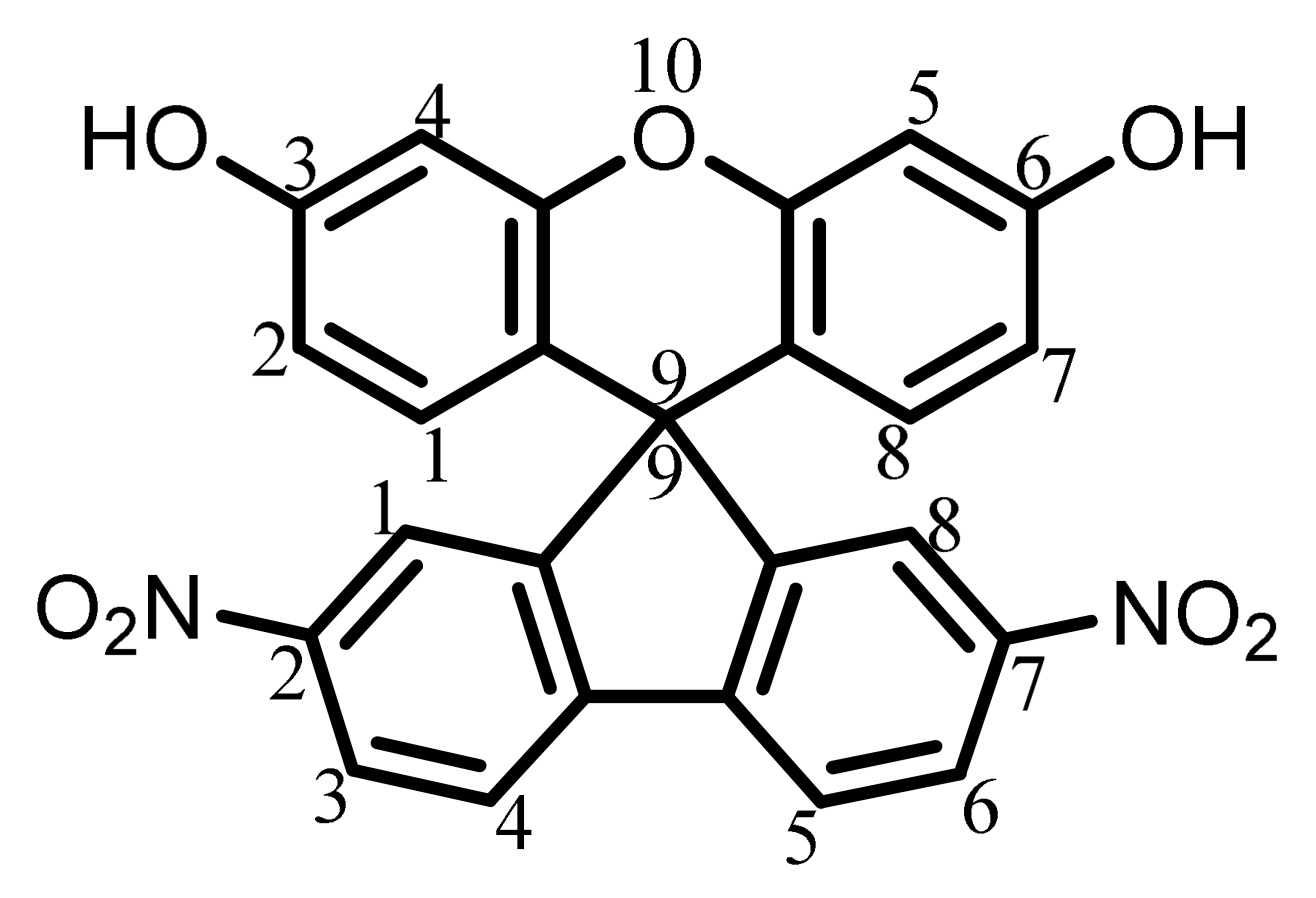
Molecular structure of *spiro*-DHX-DNFL with numbering.

The analytical data of the purified product are as follows. *T*_m_ = 157 °C (DSC). FT-IR (KBr plate method, cm^−1^): 3466 (O–H), 3064 (C_arom_–H), 1615 (biphenyl group in FL), 1521/1342 (NO_2_), 1502 (1,4-phenylene group), 1212 (C_arom_–O–C_arom_). ^1^H-NMR (400 MHz, DMSO-*d*_6_, *δ*, ppm): 9.82 [s, 2H (2.00H), OH], 8.45 [d, 2H (2.08H), *J* = 8.4 Hz, 4,5-protons of FL], 8.37 [dd, 2H (2.05H), *J* = 8.5, 2.1 Hz, 3,6-protons of FL], 7.81 [d, 2H (2.09H), *J* = 2.0 Hz, 1,8-protons of FL], 6.67 [d, 2H (2.08H), *J* = 2.4 Hz, 4,5-protons of XAN], 6.31 [dd, 2H (2.03H), *J* = 8.6, 2.5 Hz, 2,7-protons of XAN], 6.12 [d, 2H (2.06H), *J* = 8.6 Hz, 1,8-protons of XAN]. Elemental analysis: Calcd. (%) for C_25_H_14_O_7_N_2_ (454.40 g/mol): C, 66.08; H, 3.11; N, 6.17. Found: C, 65.89; H, 3.29; N, 6.20. These results confirm that the product is the desired dinitro bisphenol (*spiro*-DHX-DNFL) shown in Scheme 15.

*spiro*-C_6_X-DNFL. In a three-neck 200 mL flask, *spiro*-DHX-DNFL (3.01 mmol) and BrC_6_ (9.0 mmol) were dissolved in DMF (30 mL) in the presence of K_2_CO_3_ (7.02 mmol). The reaction mixture was refluxed at 100 °C for 5 h in an N_2_ atmosphere. The reaction mixture was gradually poured into a large quantity of water. The pale-yellowish precipitate formed was dried at 70 °C for 12 h (yield: 73%). The crude product was purified by dry-charge column chromatography (eluent: ethyl acetate/*n*-hexane, 1/1, *v*/*v*), as mentioned above (purification yield: 45%).

**Scheme 16:**
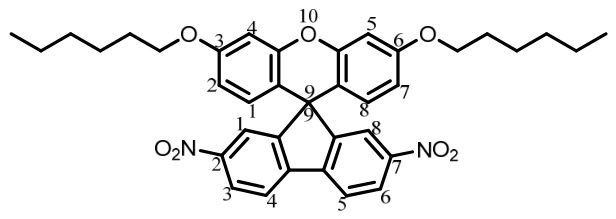
Molecular structure of *spiro*-C_6_X-DNFL with numbering.

The analytical data of the purified product are as follows. *T*_m_ = 135 °C (DSC). FT-IR (KBr plate method, cm^−1^): 3082 (C_arom_–H), 2953/2928/2870 (C_aliph_–H), 1613 (biphenyl group in FL), 1522/1340 (NO_2_), 1259/1186 (C_arom_–O–C_arom_), and the absence of the absorption bands at 3400–3500 (O–H) from the unreacted *spiro*-DHX-DNFL. ^1^H-NMR (400 MHz, DMSO-*d*_6_, *δ*, ppm): 8.47 [d, 2H (2.00H), *J* = 8.4 Hz, 4,5-protons of FL], 8.39 [dd, 2H (2.00H), *J* = 8.5, 2.1 Hz, 3,6-protons of FL], 7.83 [d, 2H (2.07H), *J* = 2.1 Hz, 1,8-protons of FL], 6.87 [d, 2H (2.08H), *J* = 2.5 Hz, 4,5-protons of XAN], 6.47 [dd, 2H (2.06H), *J* = 8.8, 2.5 Hz, 2,7-protons of XAN], 6.21 [d, 2H (2.02H), *J* = 8.7 Hz, 1,8-protons of XAN], 3.96 [t, 4H (4.02H), *J* = 6.4 Hz, O–CH_2_–(CH_2_)_4_–CH_3_], 1.68 [quin (not well-resolved), 4H (4.06H), O–CH_2_–CH_2_–(CH_2_)_3_–CH_3_], 1.38 [quin (not well-resolved), 4H, O–(CH_2_)_2_–CH_a2_–(CH_2_)_2_–CH_3_], 1.30–1.26 [m, 8H, O–(CH_2_)_3_–(CH_b2_)_2_–CH_3_], H_a_ (4H) + H_b_ (8H) = 12H (12.12H), 0.86 [t, 6H (6.01H), *J* = 7.0 Hz, the terminal CH_3_ group]. Elemental analysis: Calcd. (%) for C_37_H_38_O_7_N_2_ (622.72 g/mol): C, 71.37; H, 6.15; N, 4.50. Found: C, 71.18; H, 6.21; N, 4.45. These results confirm that the product is the desired dinitro compound (*spiro*-C_6_X-DNFL) shown in Scheme 16.

*spiro*-C_6_X-DAFL. The nitro groups in *spiro*-C_6_X-DNFL were reduced as follows. In a three-neck 300 mL flask, *spiro*-C_6_X-DNFL (1.740 mmol) was dissolved in ethanol (40 mL) in the presence of Pd/C (0.105 g), and the reaction mixture was refluxed at 80 °C for 4 h in a hydrogen atmosphere. The residual Pd/C was filtered out, and the filtrate was concentrated with an evaporator and poured gradually into a large quantity of water. The white precipitate formed was dried at 35 °C for 12 h under vacuum (yield: 68%). In contrast, when the reaction was conducted in DMF, a highly colored product was undesirably obtained after drying at 45 °C for 12 h.

**Scheme 17:**
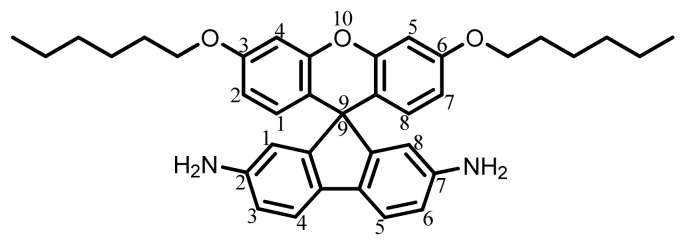
Molecular structure of *spiro*-C_6_X-DAFL with numbering.

The analytical data of the white product are as follows. *T*_m_ = 50 °C (DSC, broad). FT-IR (KBr plate method, cm^−1^): 3462/3374/3213 (N–H), 3032/3006 (C_arom_–H), 2953/2930/2859 (C_aliph_–H), 1614 (biphenyl group in FL), 1254/1178 (C_arom_–O–C_arom_). ^1^H-NMR (400 MHz, DMSO-*d*_6_, *δ*, ppm): 7.31 [d, 2H (2.00H), *J* = 8.1 Hz, 4,5-protons of FL], 6.71 [d, 2H (2.06H), *J* = 2.6 Hz, 1,8-protons of FL], 6.48–6.44 [m, 4H (4.29H), 3,6-protons of FL + 2,7-protons of XAN], 6.22 [d, 2H (2.06H), *J* = 8.7 Hz, 1,8-protons of XAN], 6.15 [d, 2H (2.09H), *J* = 2.0 Hz, 4,5-protons of XAN], 4.93 [s, 4H (4.02H), NH_2_], 3.93 [t, 4H (4.57H), *J* = 6.5 Hz, O–CH_2_–(CH_2_)_4_–CH_3_], 1.68 [quin, 4H (4.62H), *J* = 6.9 Hz, O–CH_2_–CH_2_–(CH_2_)_3_–CH_3_], 1.39 [quin, (not well-resolved), 4H, O–(CH_2_)_2_–CH_a2_–(CH_2_)_2_–CH_3_], 1.31–1.29 [m, 8H, O–(CH_2_)_3_–(CH_b2_)_2_–CH_3_], H_a_ (4H) + H_b_ (8H) = 12H (13.69H), 0.87 [t, 6H (6.70H), *J* = 7.0 Hz, the terminal CH_3_ group]. Elemental analysis: Calcd. (%) for C_37_H_42_O_3_N_2_ (562.75 g/mol): C, 78.97; H, 7.52; N, 4.98. Found: C, 78.74; H, 7.61; N, 4.95. These results confirm that the product is the desired diamine (*spiro*-C_6_X-DAFL) shown in Scheme 17.

**Scheme 18:**
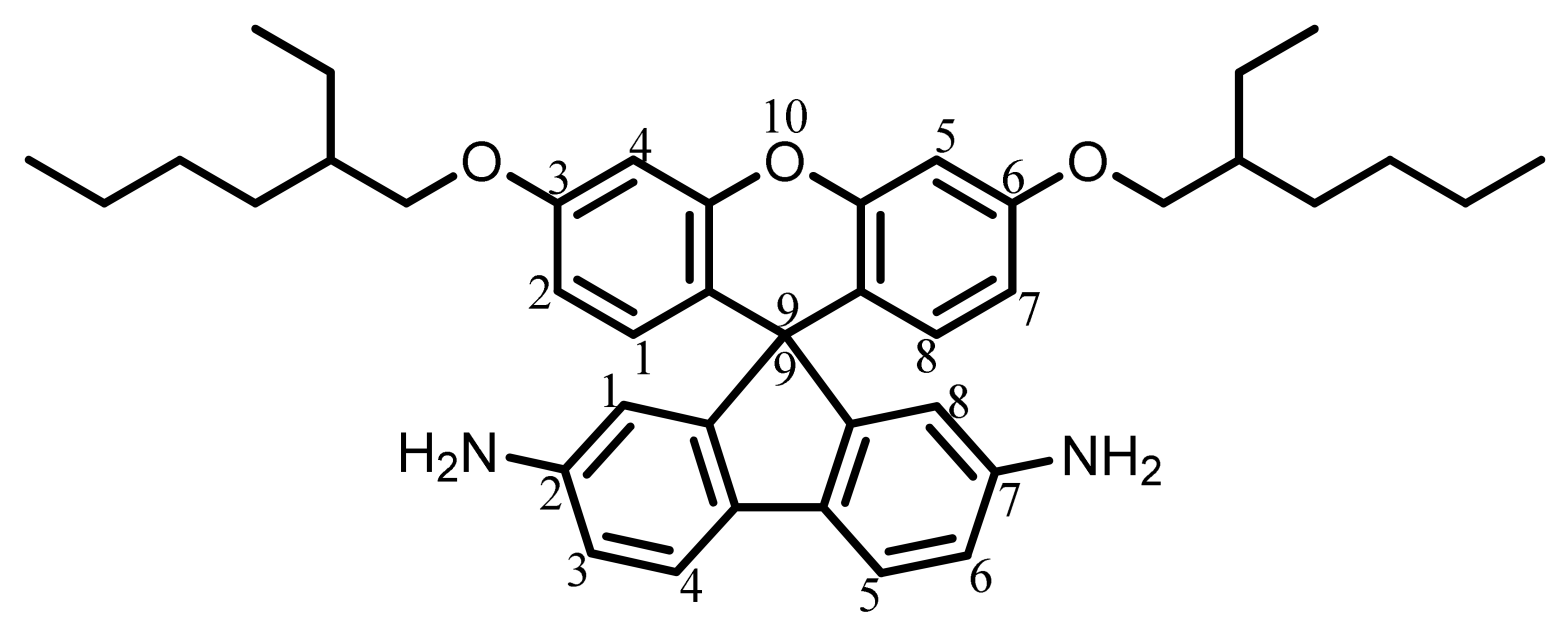
Molecular structure of *spiro*-C_8_X-DAFL with numbering.

*spiro*-C_12_X-DAFL. An analogue of *spiro*-C_6_X-DAFL, *spiro*-C_8_X-DAFL with a 2-ethylhexyl group, was synthesized using 1-bromo-2-ethylhexane (Br2EH) instead of BrC_6_ in a similar manner. The analytical data of the product are as follows. *T*_m_ = 42 °C (DSC, broad). FT-IR (KBr plate method, cm^−1^): 3464/3375/3213 (NH_2_, N–H stretching), 3029/3006 (C_arom_–H), 2958/2927/2872 (C_aliph_–H), 1614 (biphenyl group in FL), 1254/1180 (C_arom_–O–C_arom_). ^1^H-NMR (400 MHz, DMSO-*d*_6_, *δ*, ppm): 7.31 [d, 2H (2.06H), *J* = 8.0 Hz, 4,5-protons of FL], 6.72 [d, 2H (2.00H), *J* = 2.5 Hz, 1,8-protons of FL], 6.49–6.46 [m, 4H (4.24H), 3,6-protons of FL + 2,7-protons of XAN], 6.23 [d, 2H (2.09H), *J* = 8.7 Hz, 1,8-protons of XAN], 6.16 [d, 2H (2.14H), *J* = 2.0 Hz, 4,5-protons of XAN], 4.93 [s, 4H (4.27H), NH_2_], 3.84 [d, 4H (4.06H), *J* = 5.7 Hz, O–CH_2_–CH(CH_2_CH_3_)–(CH_2_)_3_–CH_3_], 1.65 [sextet (not well-resolved), 2H (2.12H), O–CH_2_–CH(CH_2_CH_3_)–(CH_2_)_3_–CH_3_], 1.45–1.28 [m, 16H (18.18H), O–CH_2_–CH(CH_2_CH_3_)–(CH_2_)_3_–CH_3_], 0.90–0.86 [m, 12H (14.02H), O–CH_2_–CH(CH_2_CH_3_)–(CH_2_)_3_–CH_3_]. Elemental analysis: Calcd. (%) for C_41_H_50_O_3_N_2_ (618.86 g/mol): C, 79.57; H, 8.14; N, 4.53. Found: C, 79.15; H, 8.06; N, 4.55. These results confirm that the product is the desired diamine (*spiro*-C_8_X-DAFL) shown in Scheme 18.

(f) *spiro*-type benzoyloxy-substituted ester-linked XAN-pendant 2,7-diaminofluorenes

This type of diamine was synthesized according to the reaction schemes (Figure 6) through the following intermediates.

*spiro*-TFBzX-DNFL. In a septum cap-sealed 100 mL flask, the afore-mentioned *spiro*-DHX-DNFL (5.38 mmol) was dissolved in anhydrous THF (20 mL) in the presence of pyridine (1.30 mL, 16.1 mmol). This solution was added gradually to 4-(trifluoromethyl)benzoyl chloride (4TFBC, 2.70 mL, 18.25 mmol) in another septum cap-sealed flask maintained at 0 °C with a syringe while magnetically stirring. The reaction mixture was stirred at 0 °C for several hours, and subsequently, for 12 h at room temperature. After the white precipitate (pyridine-HCl salt) was filtered out, the filtrate was gradually poured into *n*-hexane (800 mL). The white precipitate yielded was collected by filtration and washed with *n*-hexane, cold 2-propanol, and water, and dried at 100 °C for 12 h under vacuum (yield: 77%, Figure 7c).

**Scheme 19:**
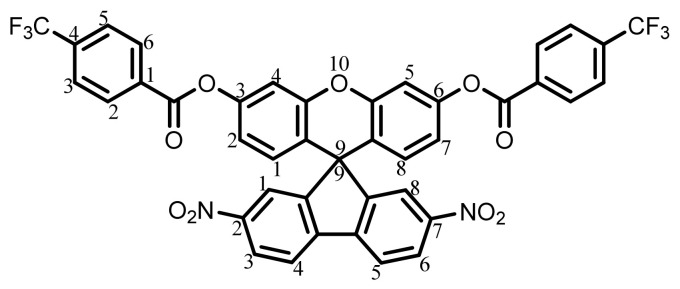
Molecular structure of *spiro*-TFBzX-DNFL with numbering.

The analytical data of the product are as follows. *T*_m_ = 283 °C (DSC). FT-IR (KBr plate method, cm^−1^): 3090 (C_arom_–H), 1748 (ester, C=O), 1608 (biphenyl group in FL), 1528/1325 (NO_2_), 1490 (1,4-phenylene group), 1341/1153 (C–F), 1263 (C_arom_–O–C_arom_). ^1^H-NMR (400 MHz, DMSO-*d*_6_, *δ*, ppm): 8.54 [d, 2H (2.00H), *J* = 8.5 Hz, 4,5-protons of FL], 8.46 [dd, 2H (1.98H), *J* = 8.5, 2.1 Hz, 3,6-protons of FL], 8.31 [d, 4H (4.06H), *J* = 8.2 Hz, 2,2′,6,6′-protons of the terminal benzoyl (Bz) group], 8.07 [d, 2H (1.94H), *J* = 2.1 Hz, 1,8-protons of FL], 7.99 [d, 4H (3.96H), *J* = 8.4 Hz, 3,3′,5,5′-protons of Bz], 7.50 [d, 2H (1.94H), *J* = 2.3 Hz, 4,5-protons of XAN], 6.93 [dd, 2H (2.03H), *J* = 8.6, 2.4 Hz, 2,7-protons of XAN], 6.50 [d, 2H (1.98H), *J* = 8.6 Hz, 1,8-protons of XAN]. Elemental analysis: Calcd. (%) for C_41_H_20_O_9_N_2_F_6_ (798.61 g/mol): C, 61.66; H, 2.52; N, 3.51. Found: C, 61.53; H, 2.68; N, 3.63. These results confirm that the product is the desired dinitro compound (*spiro*-TFBzX-DNFL) shown in Scheme 19.

*spiro*-TFBzX-DAFL. In a three-neck 100 mL flask, *spiro*-TFBzX-DNFL (4.643 mmol) was dissolved in ethyl acetate (36.4 mL) in the presence of Pd/C (0.371 g). The reaction mixture was refluxed at 50 °C for 4 h in a hydrogen atmosphere. After the Pd/C residue was filtered out, the filtrate was gradually poured into *n*-hexane (700 mL). The cream-colored precipitate formed was collected by filtration and dried at 80 °C for 12 h under vacuum (yield: 87%, Figure 7d). The crude product was recrystallized from ethyl acetate/*n*-hexane (5/22, *v*/*v*), and the cream-colored precipitate was dried at 80 °C for 12 h (recrystallization yield: 74%). The purified product was additionally decolorized, even though its chemical purity was sufficiently high. The adsorption method using activated carbon and activated clay in various polar solvents (methanol, acetone, acetic acid, DMSO, ethyl acetate) in the presence of water was less effective at decolorizing. In contrast, neutralization of an acetic acid solution of the product with a 1N-NaOH aqueous solution was effective. The white precipitate yielded was repeatedly washed with water and dried at 100 °C for 12 h (decolorization yield: 95%, Figure 7e).

**Figure 8:**
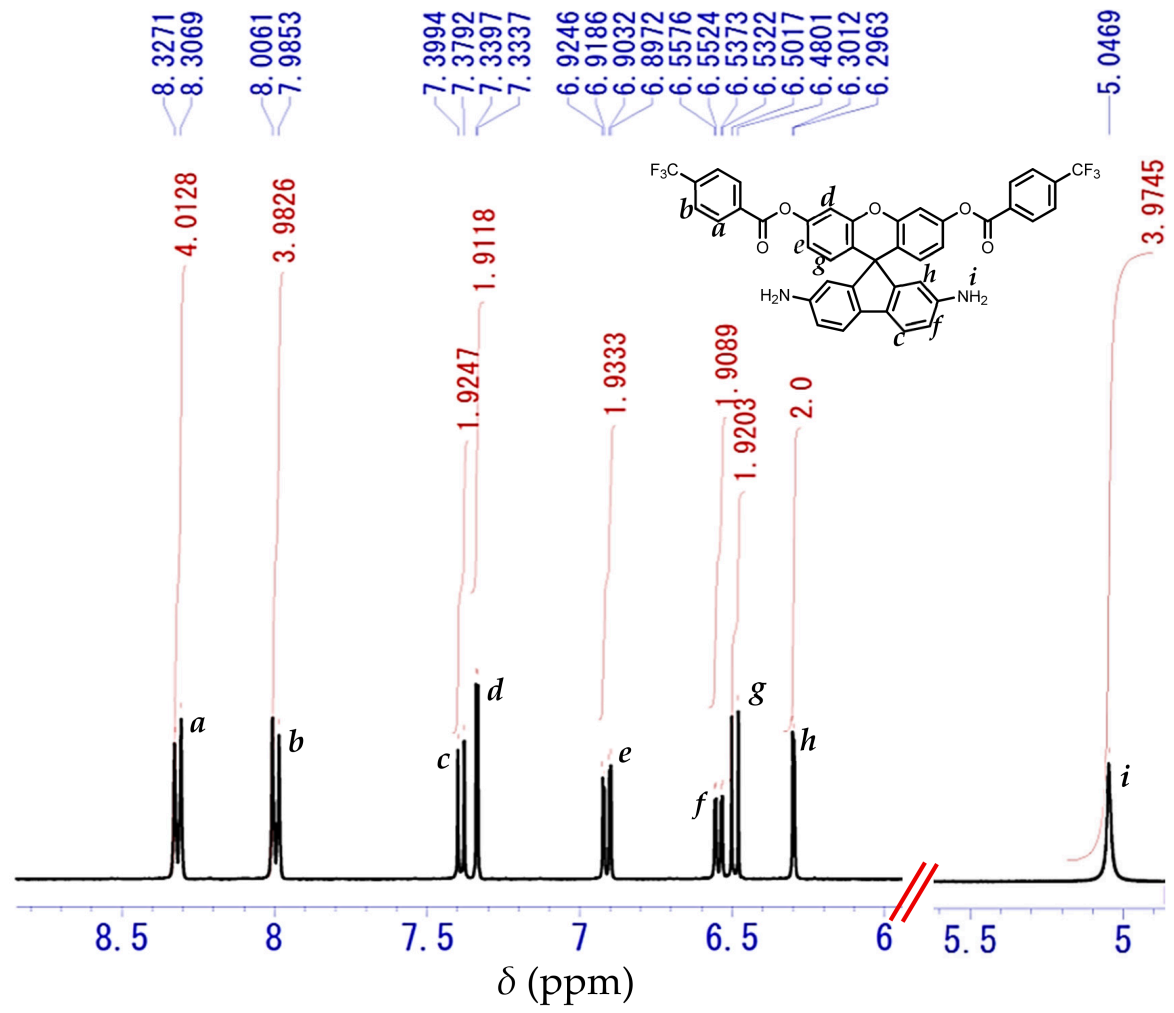
^1^H-NMR spectrum (400 MHz, DMSO-*d*_6_) of *spiro*-TFBzX-DAFL purified by recrystallization and subsequent decolorization.

**Scheme 20:**
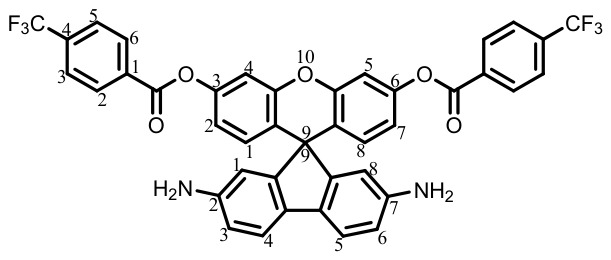
Molecular structure of *spiro*-TFBzX-DAFL with numbering.

The recrystallized and decolorized product maintained an extremely high chemical purity, as suggested by the following analytical data. FT-IR (KBr plate method, cm^−1^): 3464/3378/3221 (NH_2_, N–H stretching), 3070/3008 (C_arom_–H), 1742 (ester, C=O), 1618 (biphenyl group in FL), 1489 (1,4-phenylene group), 1326/1151 (C–F), 1262 (C_arom_–O–C_arom_). The ^1^H-NMR spectrum (Figure 8) and its detailed data (400 MHz, DMSO-*d*_6_, *δ*, ppm): 8.32 [d, 4H (4.01H), *J* = 8.1 Hz, 2,2′,6,6′-protons of Bz], 7.99 [d, 4H (3.98H), *J* = 8.3 Hz, 3,3′,5,5′-protons of Bz], 7.39 [d, 2H (1.93H), *J* = 8.1 Hz, 4,5-protons of FL], 7.34 [d, 2H (1.91H), *J* = 2.4 Hz, 4,5-protons of XAN], 6.91 [dd, 2H (1.93H), *J* = 8.6, 2.4 Hz, 2,7-protons of XAN], 6.54 [dd, 2H (1.91H), *J* = 8.1, 2.1 Hz, 3,6-protons of FL], 6.49 [d, 2H (1.92H), *J* = 8.6 Hz, 1,8-protons of XAN], 6.30 [d, 2H (2.00H), *J* = 2.0 Hz, 1,8-protons of FL], 5.05 [s, 4H (3.98H), NH_2_]. Elemental analysis: Calcd. (%) for C_41_H_24_O_5_N_2_F_6_ (738.64 g/mol): C, 66.67; H, 3.28; N, 3.79. Found: C, 66.45; H, 3.39; N, 3.77. These results confirm that the product is the desired diamine (*spiro*-TFBzX-DAFL) shown in Scheme 20.

**Scheme 21:**
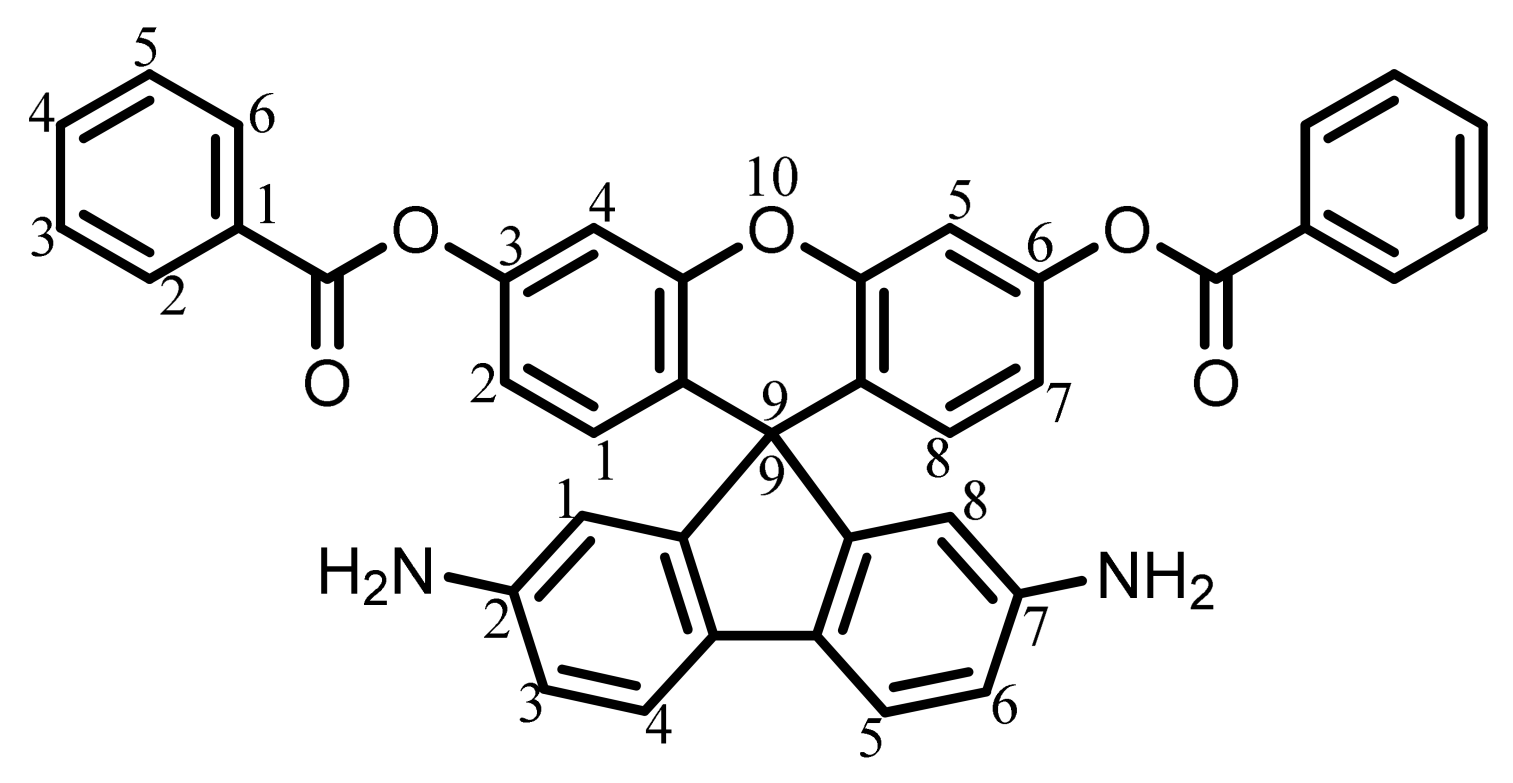
Molecular structure of *spiro*-BzX-DAFL with numbering.

*spiro*-BzX-DAFL. An analogue of *spiro*-TFBzX-DAFL, *spiro*-BzX-DAFL without the CF_3_ groups, was synthesized according to the reaction scheme (Figure 6) using benzoyl chloride (BzC) instead of 4TFBC in a similar manner. The analytical data of the product are as follows. FT-IR (KBr plate method, cm^−1^): 3445/3363/3218 (NH_2_, N–H stretching), 3060/3033/3008 (C_arom_–H), 1733 (ester, C=O), 1607 (biphenyl group in FL), 1487 (1,4-phenylene group), 1239 (C_arom_–O–C_arom_). ^1^H-NMR (400 MHz, DMSO-*d*_6_, *δ*, ppm): 8.12 [dd, 4H (4.00H), *J* = 7.4, 2.2 Hz, 2,2′,6,6′-protons of Bz], 7.76 [m (different from dd), 2H (1.95H), 4,4′-protons of Bz], 7.61 [t, 4H (3.92H), *J* = 7.7 Hz, 3,3′,5,5′-protons of Bz], 7.39 [d, 2H (1.93H), *J* = 8.0 Hz, 4,5-protons of FL], 7.28 [d, 2H (1.92H), *J* = 2.3 Hz, 4,5-protons of XAN], 6.87 [dd, 2H (1.92H), *J* = 8.6, 2.4 Hz, 2,7-protons of XAN], 6.54 [dd, 2H (1.95H), *J* = 8.1, 2.1 Hz, 3,6-protons of FL], 6.47 [d, 2H (1.94H), *J* = 8.6 Hz, 1,8-protons of XAN], 6.30 [d, 2H (1.92H), *J* = 2.0 Hz, 1,8-protons of FL], 5.04 [s, 4H (4.05H), NH_2_]. These results confirm that the product is the desired diamine (*spiro*-BzX-DAFL) shown in Scheme 21.

(g) FL-pendant 2,3,6,7-xanthenetetracarboxylic dianhydride

**Figure 9:**
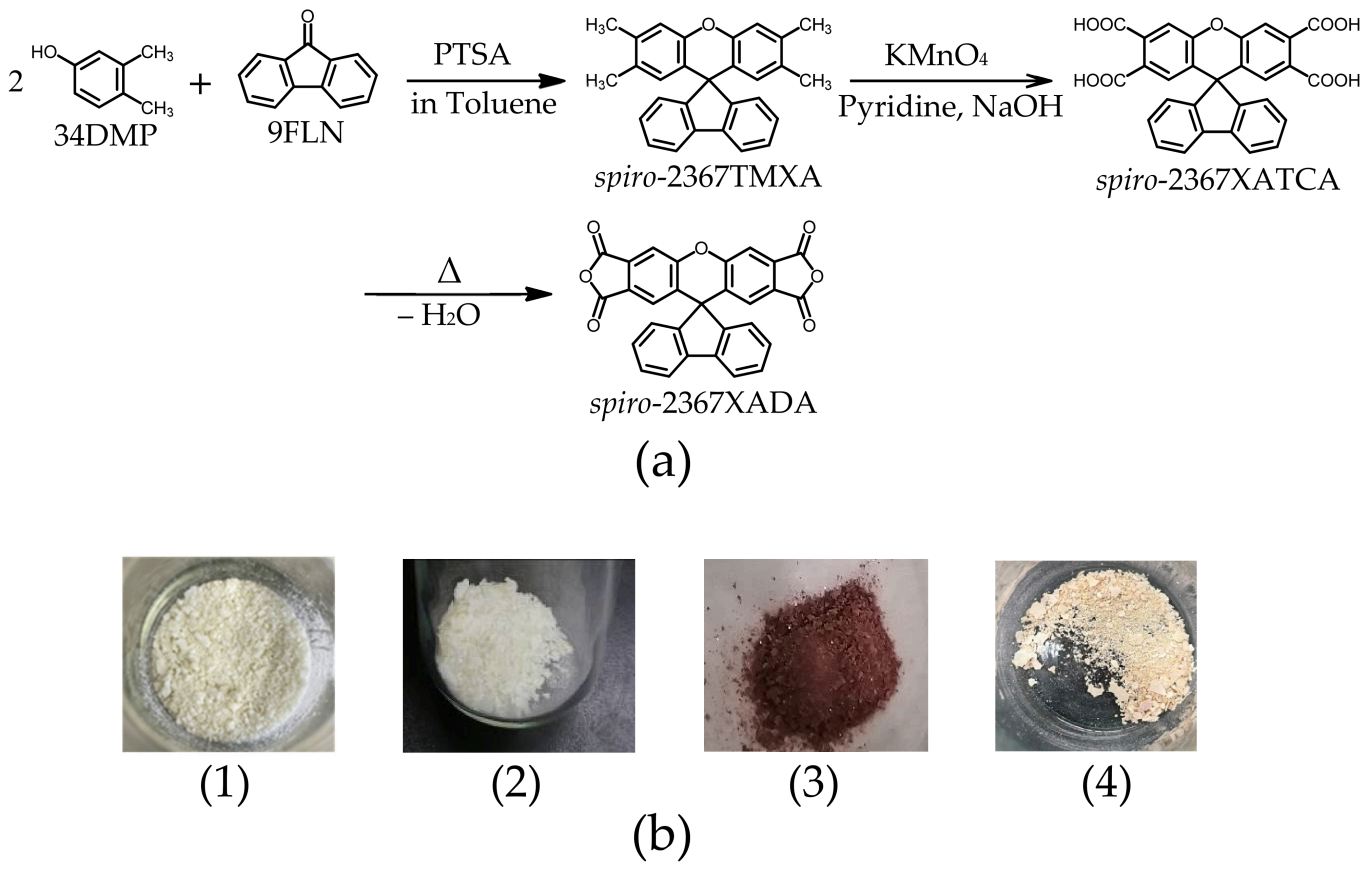
The manufacturing process of fluorene-pendant 2,3,6,7-xanthenetetracarboxylic dianhydride (*spiro*-2367XADA): (**a**) The synthetic scheme and (**b**) the appearance of the intermediates and purified product (1: *spiro*-2367TMXA; 2: *spiro*-2367XATCA; 3: crude *spiro*-2367XADA formed upon thermal cyclodehydration of *spiro*-2367XATCA; and 4: purified *spiro*-2367XADA by decolorization using activated carbon and subsequent recrystallization from acetic acid).

This TCDA was synthesized according to the reaction scheme (Figure 9a) through the following intermediates.

*spiro*-2367TMXA. In a three-neck 300 mL flask, 9FLN (19.45 mmol) and 3,4-dimethylphenol (34DMP, 94.81 mmol) were dissolved in toluene (10 mL) in the presence of PTSA (8.09 mmol), and the reaction mixture was refluxed at 120 °C for 6 h in an N_2_ atmosphere. After dilution with toluene (80 mL), the solution was washed with a 5N-NaOH aqueous solution (30 mL) in a separating funnel to remove an excess of 34DMP. The precipitate formed was removed by filtration. The filtrate was repeatedly washed with water in a separating funnel, and the organic phase was dried with Na_2_SO_4_. The white precipitate obtained by solvent removal was washed with *n*-hexane and dried at 100 °C for 12 h under vacuum [yield: 55%, Figure 9b(1)].

**Scheme 22:**
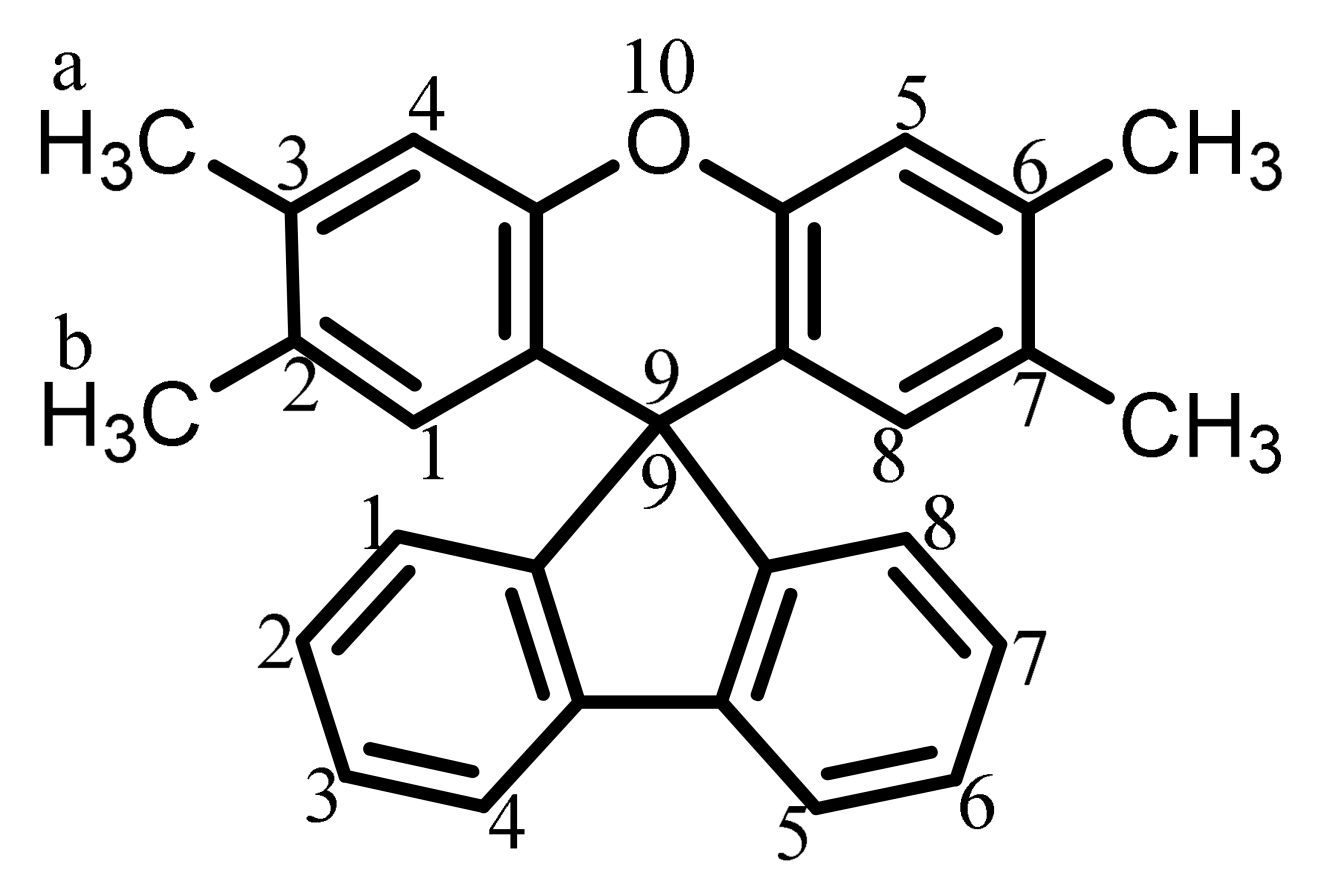
Molecular structure of *spiro*-2367TMXA with numbering.

The analytic data of the product are as follows. *T*_m_ = 225 °C (TG-DTA, broad). FT-IR (KBr plate method, cm^−1^): 3063/3023 (C_arom_–H), 2967/2941/2918/2862 (C_aliph_–H), 1494 (phenyl group), 1259/1125 (C_arom_–O–C_arom_). ^1^H-NMR (400 MHz, DMSO-*d*_6_, *δ*, ppm): 7.96 [d, 2H (2.01H), *J* = 7.6 Hz, 4,5-protons of FL], 7.39 [t, 2H (2.04H), *J* = 7.4 Hz, 3,6-protons of FL], 7.23 [t, 2H (1.99H), *J* = 7.4 Hz, 2,7-protons of FL], 7.04 [s, 2H (2.07H), 4,5-protons of XAN], 7.01 [d, 2H (2.04H), *J* = 7.6 Hz, 1,8-protons of FL], 5.95 [s, 2H (2.00H), 1,8-protons of XAN], 2.15 [s, 6H (6.01H), CH_a3_], 1.84 [s, 6H (5.96H), CH_b3_]. Elemental analysis: Calcd. (%) for C_29_H_24_O (388.51 g/mol): C, 89.66; H, 6.23. Found: C, 89.83; H, 6.23. These results confirm that the product is the desired compound (*spiro*-2367TMXA) shown in Scheme 22.

*spiro*-2367XATCA. In a three-neck 300 mL flask shaded with an aluminum foil, *spiro*-2367TMXA (4.96 mmol) was dissolved in a mixed solvent of pyridine (50 mL) and water (5 mL) at 120 °C. To this solution, KMnO_4_ (61.1 mmol) was gradually added in five portions at about 30 min intervals, and the reaction mixture was additionally refluxed at 120 °C for 2.5 h. After an NaOH aqueous solution (4 wt%, 100 mL) was added, KMnO_4_ (20.4 mmol) was additionally added to the resulting phase-separated reaction mixture in two portions at about 1 h intervals with vigorous magnetic stirring (here, insufficient stirring increased unintended by-products), and the reaction mixture was refluxed at 100 °C for 7.5 h; thereby, the solution changed from purple to yellow. The precipitate yielded (primarily MnO_2_) was removed by filtration. This procedure was repeated two more times to complete oxidation of the residual CH_3_ groups. This multi-step oxidation process using KMnO_4_ was the key to obtaining pure *spiro*-2367XATCA. To the yellow filtrate, conc. HCl (30 mL) was added. The white precipitate formed was collected by filtration and washed with a 2N-HCl aqueous solution (60 mL) to suppress the loss during washing due to its water solubility. The white precipitate was dissolved in anhydrous GBL, and the insoluble portion was filtered out. The white or pale-pink precipitate obtained by solvent removal of the filtrate was dried at 80 °C for 12 h under vacuum [Figure 9b(2)].

**Scheme 23:**
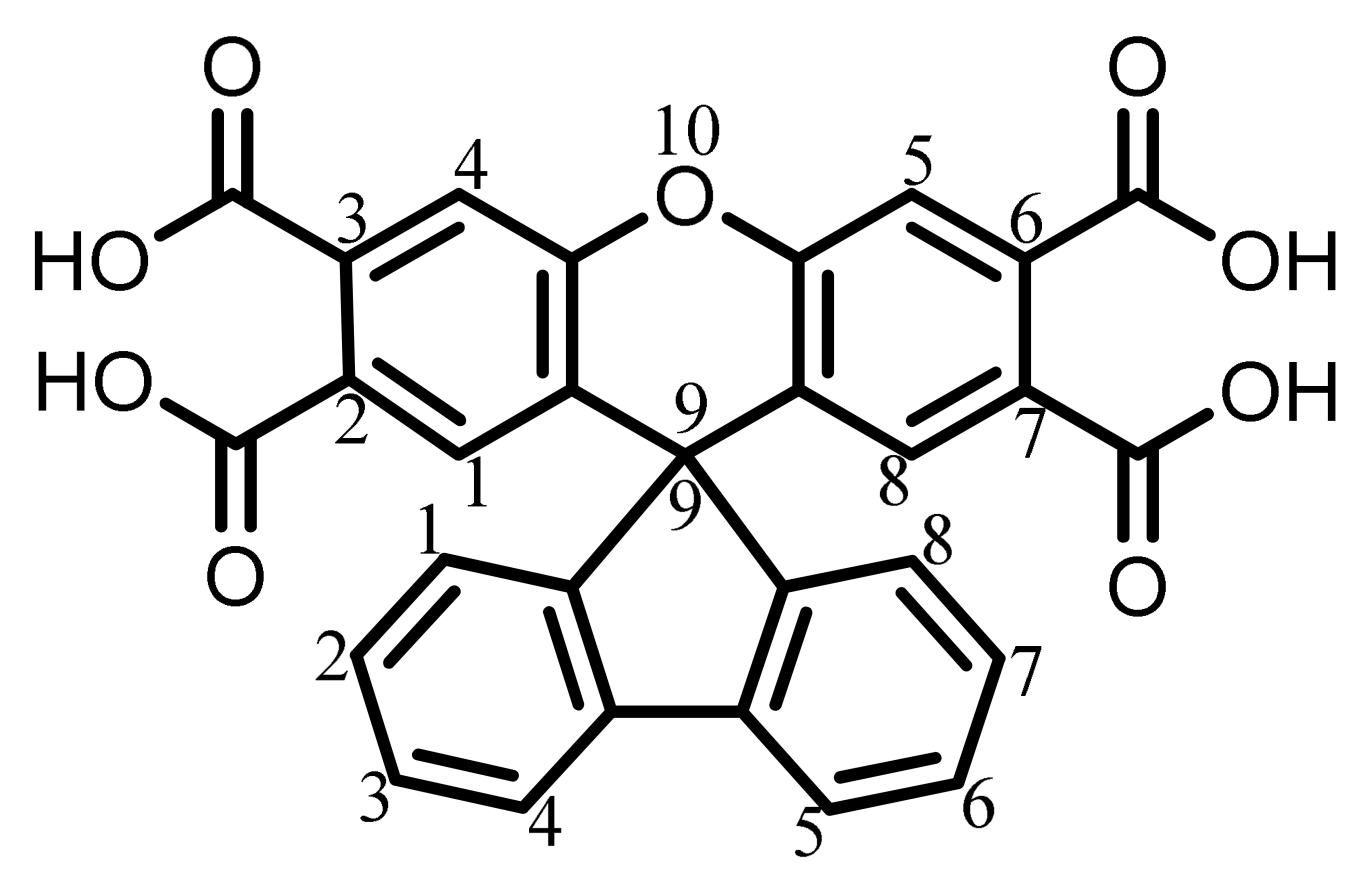
Molecular structure of *spiro*-2367XATCA with numbering.

The analytic data of the product are as follows. FT-IR (KBr plate method, cm^−1^): 3063 (C_arom_–H), 2551 (broad, hydrogen-bonded COOH, O–H stretching), 1706 (hydrogen-bonded COOH, C=O), 1494 (phenyl group), 1283 (C_arom_–O–C_arom_). ^1^H-NMR (400 MHz, DMSO-*d*_6_, *δ*, ppm): 13.5–13.0 [br, 4H, COOH], 8.07 [d, 2H (2.00H), *J* = 7.6 Hz, 4,5-protons of FL], 7.55 [s, 2H, 4,5-protons (H_a_) of XAN], 7.50 [t, 2H, *J* = 7.5 Hz, 3,6-protons (H_b_) of FL], H_a_ (2H) + H_b_ (2H) = 4H (4.21H), 7.31 [t, 2H (2.11H), *J* = 7.5 Hz, 2,7-protons of FL], 7.21 [d, 2H (1.99H), *J* = 7.5 Hz, 1,8-protons of FL], 6.63 [s, 2H (2.18H), 1,8-protons of XAN]. These results confirm that the product is the desired compound (*spiro*-2367XATCA) shown in Scheme 23.

*spiro*-2367XADA. This compound can be obtained simply via thermal cyclodehydration of *spiro*-2367XATCA. However, heating of *spiro*-2367XATCA at 200 °C under vacuum caused unexplained intense coloration [reddish-brown product, Figure 9b(3)]. The use of this intensely colored TCDA prevents the formation of colorless PI films. Therefore, the product was decolorized using activated carbon (powder form, Fujifilm Wako Pure Chemical, Osaka, Japan) as follows. After the colored *spiro*-2367XADA (2.23 mmol) was dissolved in acetic acid (111 mL), water (2 mL) and activated carbon 0.976 g) were added, and the suspension was placed at room temperature for 24 h. After the activated carbon was filtered out, the cream-yellow precipitate was obtained via azeotropic solvent evaporation with toluene [yield: 63%, Figure 9b(4)]. The decolorized product was further purified by recrystallization from anhydrous acetic acid.

**Figure 10:**
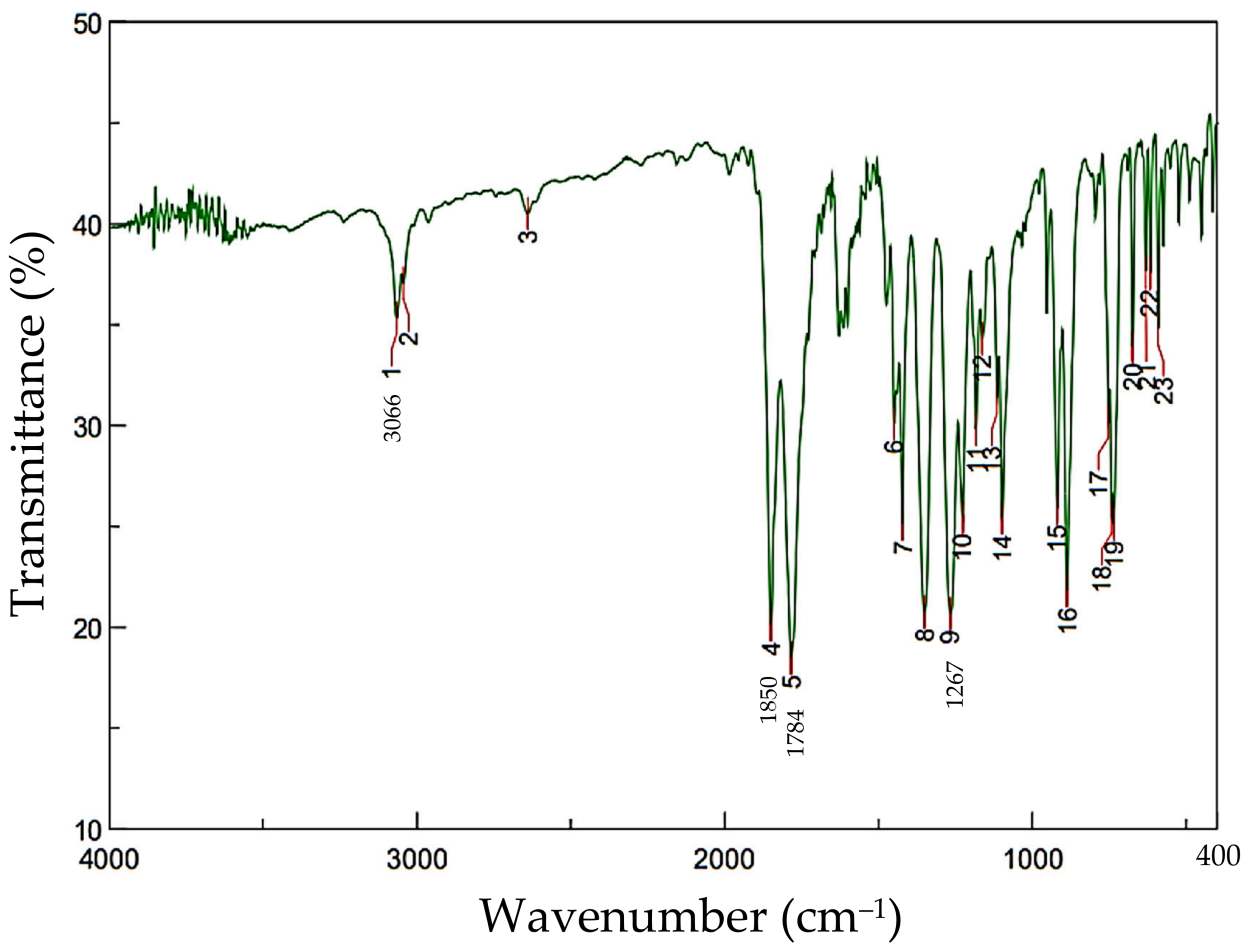
FT-IR spectrum (KBr, plate method) of purified *spiro*-2367XADA.

**Figure 11:**
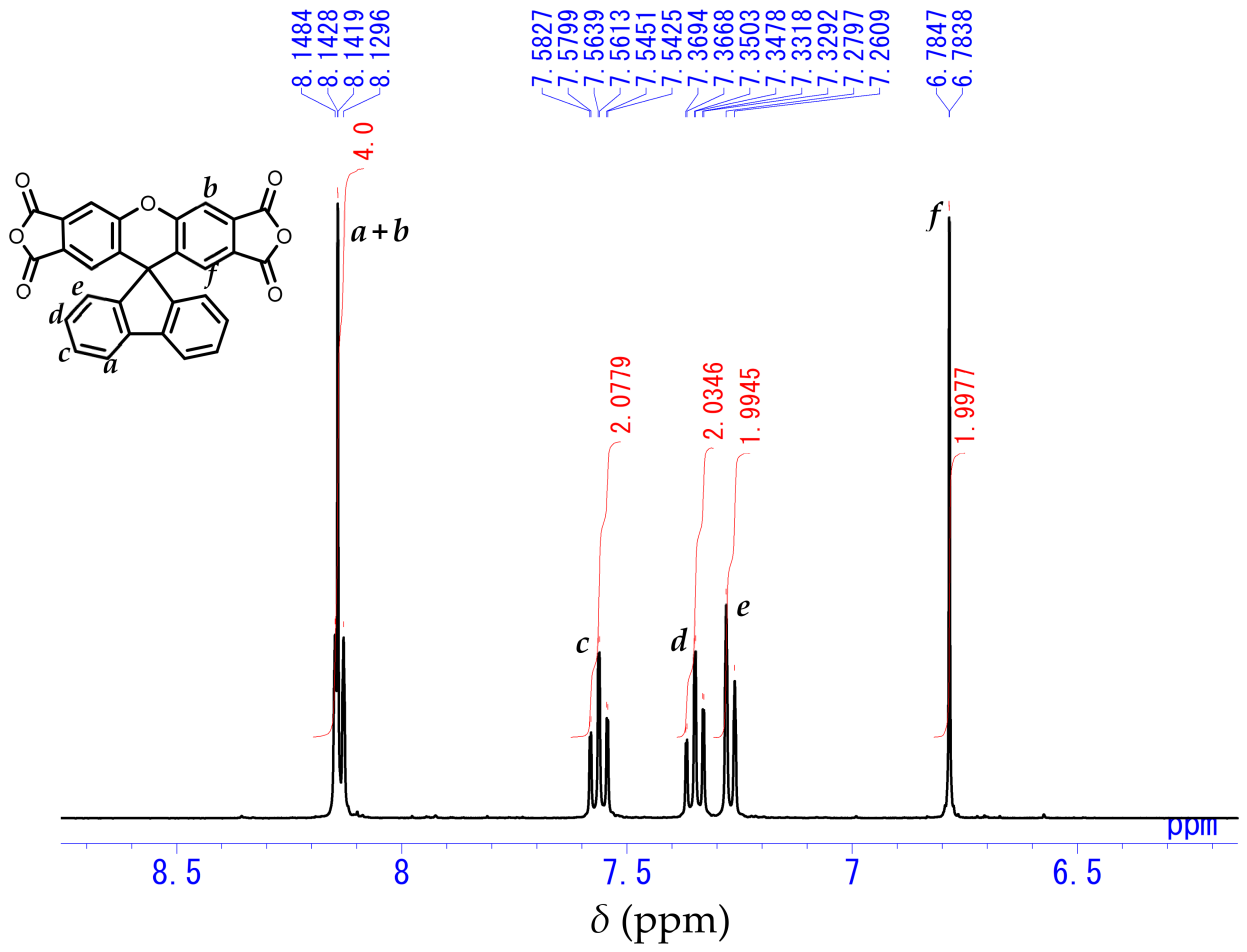
^1^H-NMR spectrum (400 MHz, DMSO-*d*_6_) of purified *spiro*-2367XADA.

**Figure 12:**
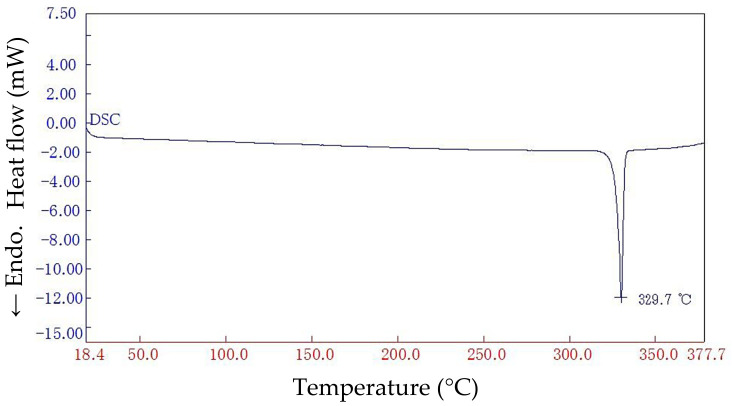
DSC thermogram of purified *spiro*-2367XADA.

**Scheme 24:**
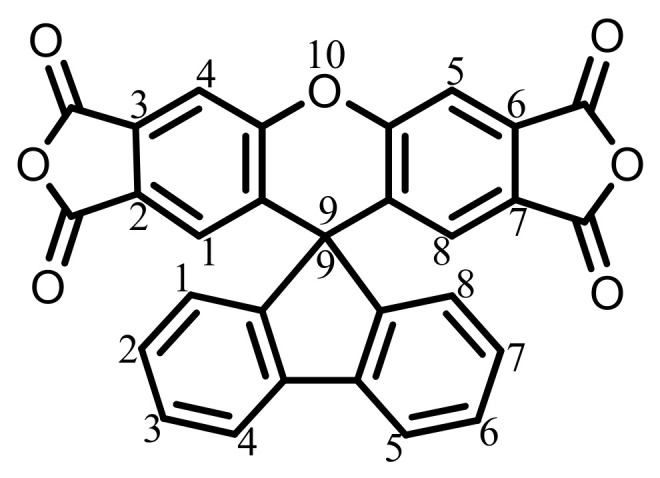
Molecular structure of *spiro*-2367XADA with numbering.

The analytical data of the purified product are as follows. The FT-IR spectrum (Figure 10) and its detailed data (KBr plate method, cm^−1^): 3066 (C_arom_–H), 1850/1784 (acid anhydride, C=O), 1267 (C_arom_–O–C_arom_). The ^1^H-NMR spectrum (Figure 11) and its detailed data (400 MHz, DMSO-*d*_6_, *δ*, ppm): 8.15–8.13 [m, 4H (3.97H), 4,5-protons of FL + 4,5-protons of XAN], 7.56 [t, 2H (2.01H), *J* = 7.5 Hz, 3,6-protons of FL], 7.35 [t, 2H (2.02H), *J* = 7.5 Hz, 2,7-protons of FL], 7.27 [d, 2H (1.98H), *J* = 7.6 Hz, 1,8-protons of FL], 6.78 [s, 2H (2.00H), 1,8-protons of XAN]. Elemental analysis: Calcd. (%) for C_29_H_12_O_7_ (472.41 g/mol): C, 73.73; H, 2.56. Found: C, 73.44; H, 2.72. The product exhibited a relatively sharp endothermic peak for melting at 330 °C on DSC (Figure 12). These results confirm that the product is the desired TCDA (*spiro*-2367XADA) shown in Scheme 24.

#### 2.1.2. Commercially Available Monomers

**Figure 13:**
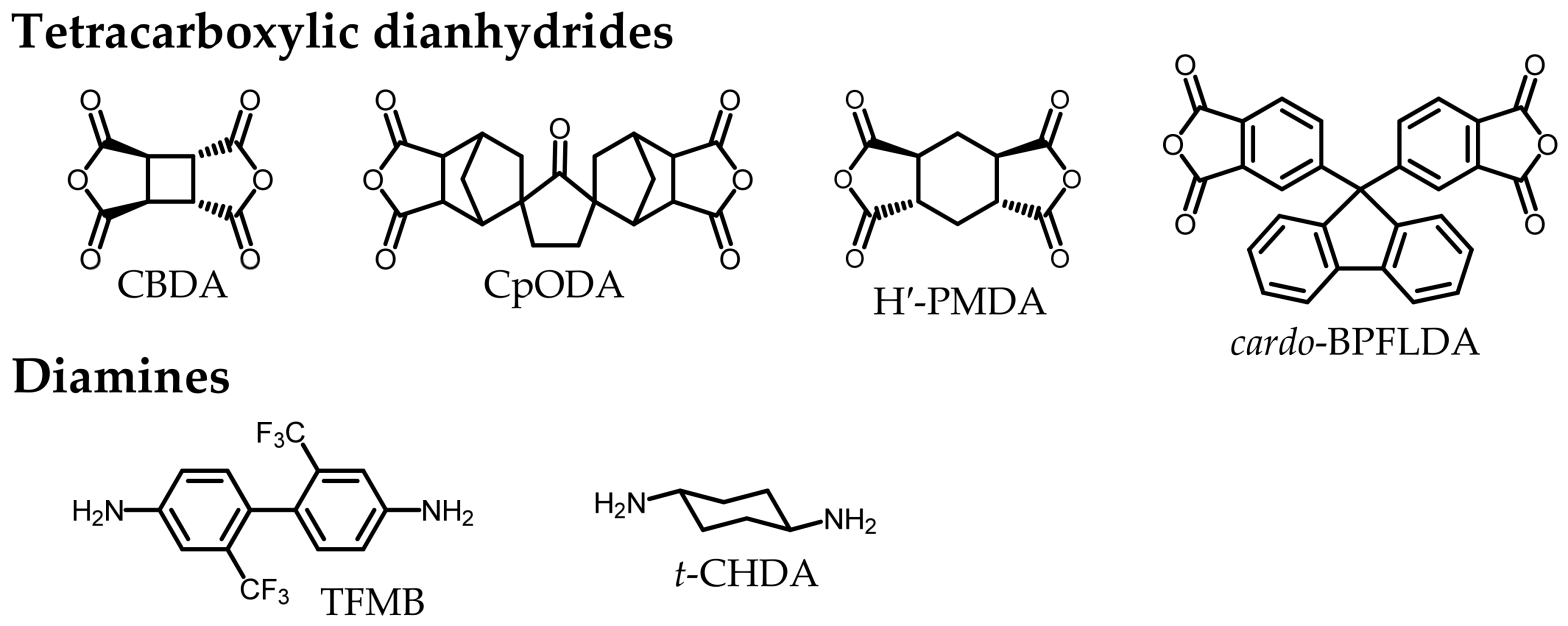
Molecular structures and abbreviations of the commercially available monomers used in this study.

The molecular structures of the commercially available monomers used in this study are shown in Figure 13. Their commercial sources, abbreviations, pre-drying conditions, and melting points are listed in Table 1.

#### 2.1.3. Polymerization and Film Preparation

**Figure 14:**
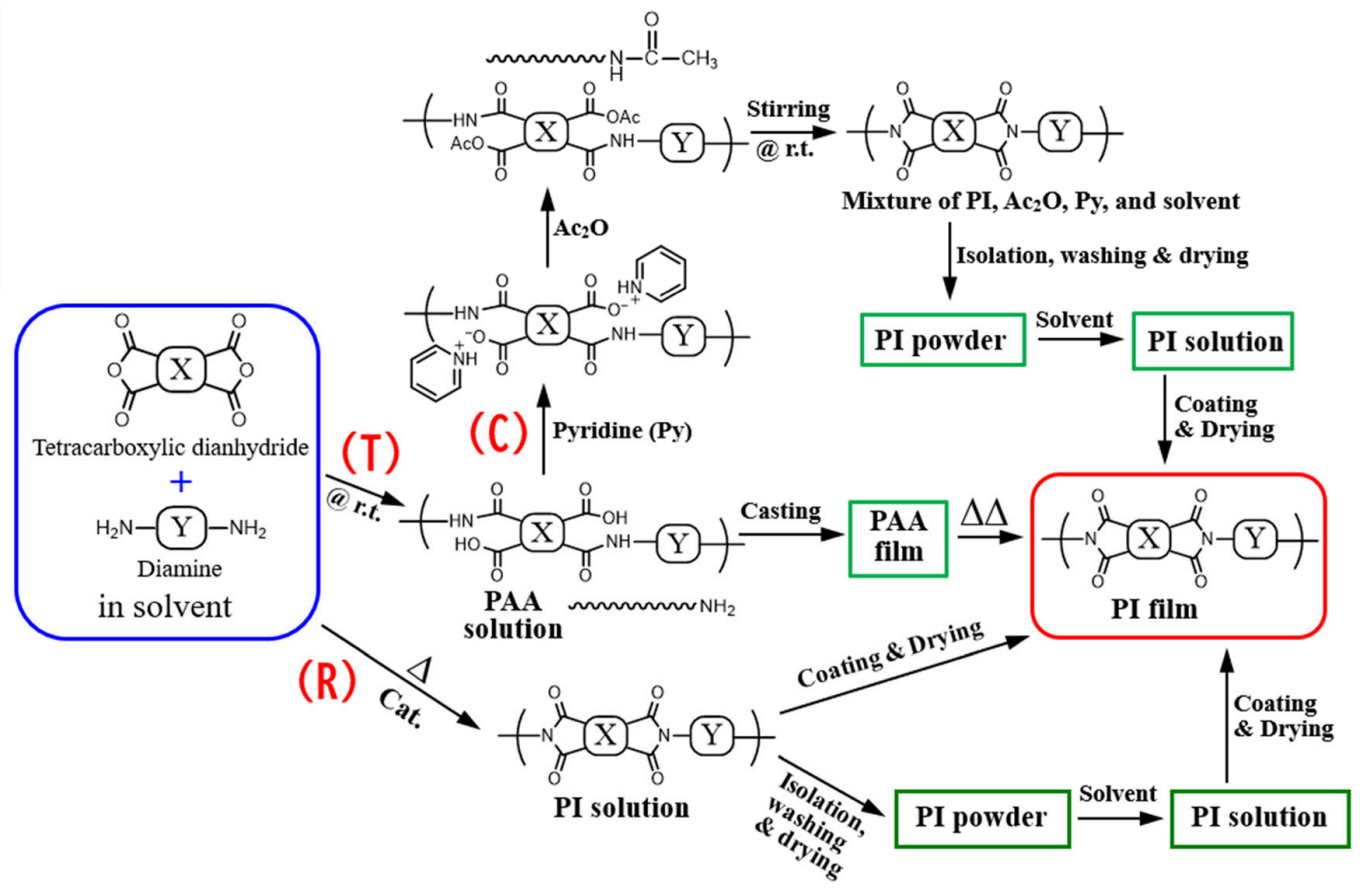
Schemes of polymerization, imidization, and film preparation via different pathways: (Route-T) Conventional two-step process, (Route-C) chemical imidization process, and (Route-R) modified one-pot process.

In this study, polymerization and film preparation were conducted through the following three processes (Figure 14). The features of these processes are summarized in Table 2. Route-T: Conventional two-step process consisting of low-temperature equimolar polyaddition, coating/drying of the resulting PAA solutions at 60 °C (for DMAc solution) or 80 °C (for *N*-methyl-2-pyrrolidone (NMP) solution) for 2 h in an air-convection oven, and thermal imidization of the PAA cast films on a glass substrate typically at 200 °C for 1 h + 250 °C for 1 h under vacuum and additional annealing at 300 °C for 1 h without the substrates under vacuum. Route-C: Chemical imidization by adding a cyclodehydration reagent [acetic anhydride (Ac_2_O)/pyridine (7/3, *v*/*v*)] with a fixed molar ratio of [Ac_2_O]/[COOH]_PAA_ = 5 to the PAA solutions (diluted as appropriate), isolation of PIs, redissolution of the isolated fibrous PI powder in a fresh solvent, and coating/drying of the resulting homogeneous PI solutions [37]. Route-R: Modified one-pot polymerization by refluxing monomer/solvent suspension in the presence of a combined catalyst, followed by isolation of PI powder, redissolution in a fresh solvent, and coating/drying of the resulting homogeneous PI solutions [39,41,47,48]. A typical procedure of the modified one-pot polymerization process is as follows. Diamine (5 mmol) and benzoic acid (BA, 10 mmol, 1 Eq.) as a cocatalyst were completely dissolved in a selected dehydrated solvent (GBL, DMAc, or NMP) in a reaction vessel at room temperature (or by mildly warming as appropriate) with continuous mechanical stirring. To this solution, 1-ethylpiperidine (1-EP, 10 mmol, 1 Eq.) as a catalyst and subsequently TCDA powder (5 mmol) were added in one portion. The reaction mixture in the sealed reaction vessel was then rapidly heated by immersing the vessel in an oil bath maintained at an established elevated temperature and refluxed typically at 200 °C or the boiling point of the solutions for 4 h in an N_2_ atmosphere with continuous mechanical stirring. The reaction was started at a total monomer content of 40 or 50 wt%, and the reaction mixture was gradually diluted as appropriate with a minimum amount of the same solvent to ensure effective mixing. In this process, we used a specially designed separable reaction vessel equipped with a dry nitrogen gas inlet and outlet connected to a silicone oil-sealed bubbler, condenser, Dean–Stark trap, and sealed mechanical stirrer with a non-contact magnetic coupling mechanism (Nakamura Scientific Instruments Industry, Tokyo, Japan, UZ-SM1). The reaction mechanism in this process is described in detail in our previous paper [48].

**Figure 15:**
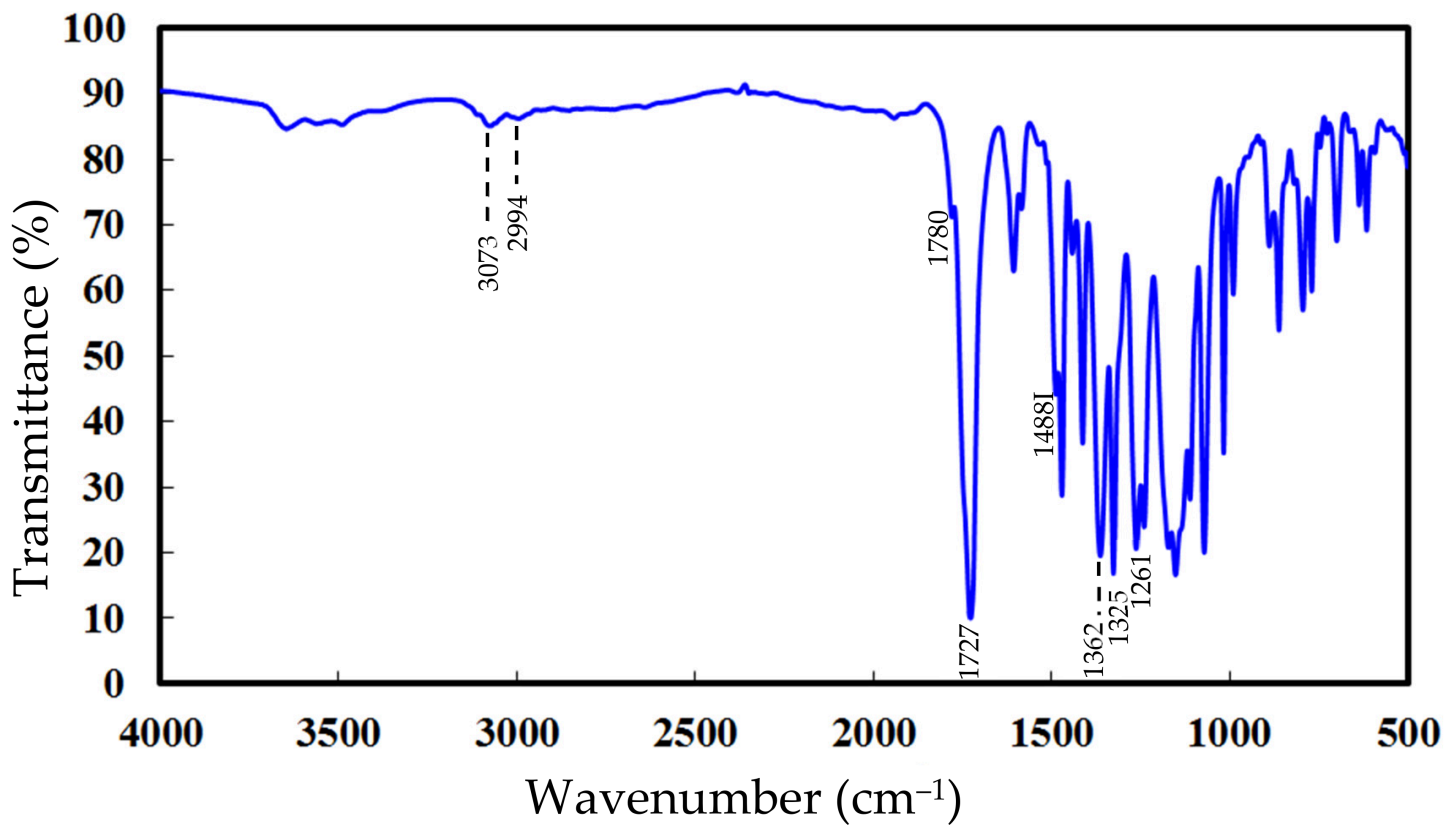
Transmission-mode FT-IR spectrum of a thin film for PI (CBDA/*spiro*-TFBzX-DAFL) system obtained via chemical imidization.

The progress of polymerization and completion of imidization were confirmed by FT-IR and/or ^1^H-NMR spectra. A typical transmission-mode FT-IR spectrum of a PI thin film prepared via Route-C is shown in Figure 15. The spectrum includes the specific bands (cm^−1^): 3073 (C_arom_–H), 2994 (C_aliph_–H), 1780 (imide, C=O), 1724 (imide, C=O + ester, C=O), 1488 (1,4-phenylene group), 1362 (imide, *N*–C_arom_), 1335 (C–F). In addition, the PAA specific bands at ~2600 cm^−1^ (hydrogen-bonded COOH, O–H stretching) and 1680/1530 cm^−1^ (amide, C=O) were not observed.

**Figure 16:**
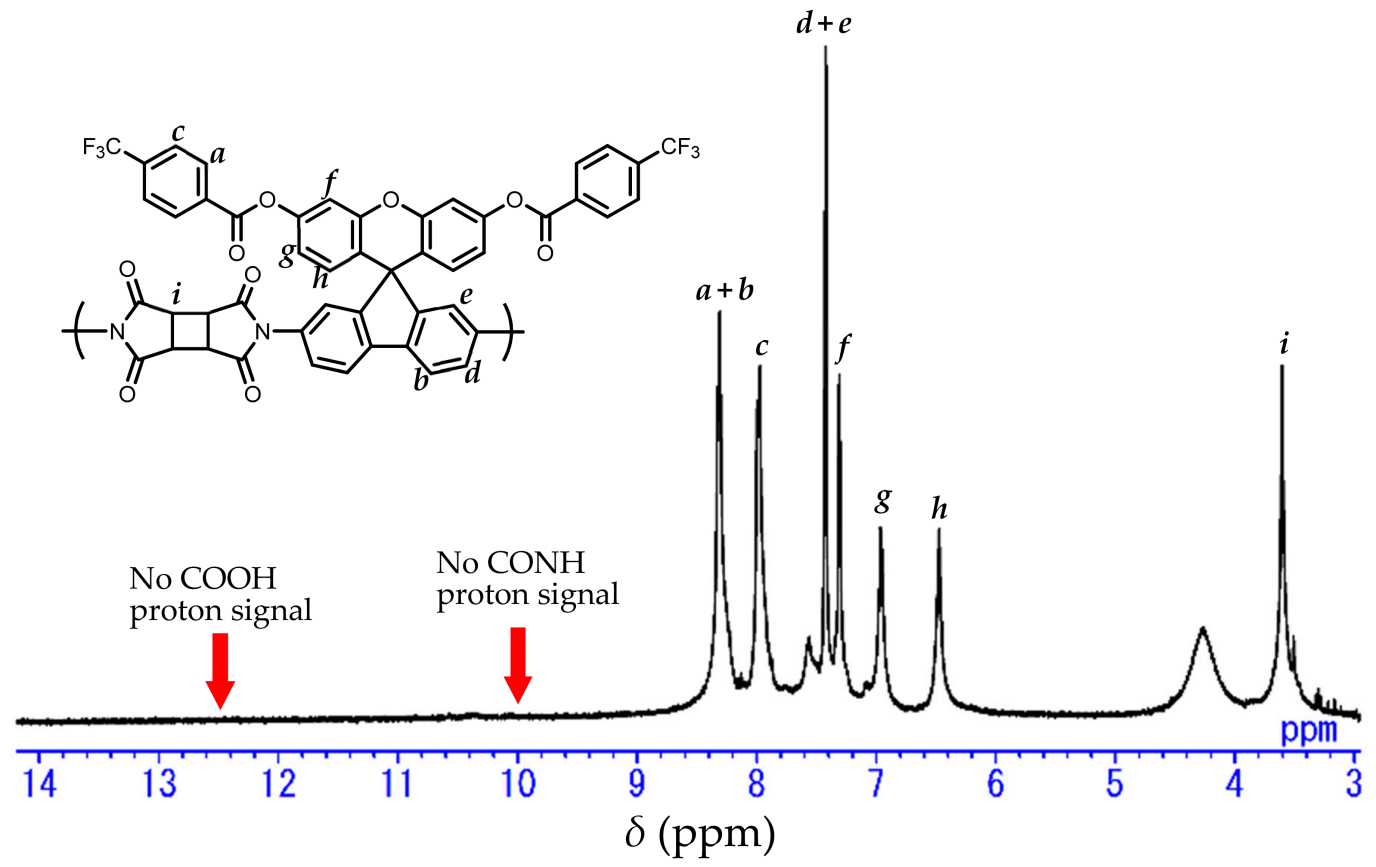
^1^H-NMR spectrum (400 MHz, DMSO-*d*_6_) of PI powder sample (CBDA/*spiro*-TFBzX-DAFL) obtained via chemical imidization.

A typical ^1^H-NMR spectrum (400 MHz, DMSO-*d*_6_) of the PI prepared via Route-C is shown in Figure 16. No NHCO and COOH proton signals due to the amide acid units were observed. These spectra confirmed the completion of chemical imidization. Similarly, complete imidization was confirmed for the PI powder samples prepared via Route-R.

The PI films (typical thickness: 20 μm) were prepared as follows. The above-mentioned isolated fibrous PI powder (Route-C and Route-R) was redissolved in a fresh solvent at a high solid content (typically, 15 wt%), and the resulting homogeneous PI solution was coated on a glass substrate and soft-dried at 60–65 °C (for DMAc solution) or 80 °C (for NMP solution) for 2 h in an air-convection oven. Then, the PI cast films were heated stepwise typically at 150 °C for 0.5 h + 200 °C for 0.5 h + 250 °C/1 h on the substrates under vacuum to completely remove the solvents. Additionally, the films were annealed at 300 °C for 1 h under vacuum without the substrates to remove residual strain in the films. These thermal conditions were adjusted as appropriate to obtain better-quality films.

In this study, the PAA and PI systems were represented using the abbreviations of the monomer symbols [tetracarboxylic dianhydrides (A) and diamines (B)] as A/B for homopolymers and A_1_;A_2_/B_1_;B_2_ for copolymers.

### 2.2. Measurements

#### 2.2.1. Structural Characterization

The molecular structures of the monomers and their intermediates synthesized in this study were characterized by FT-IR (KBr plate method, JASCO, Tokyo, Japan, FT/IR 4100 or 4600 infrared spectrometer) and ^1^H-NMR spectra in DMSO-*d*_6_ or CDCl_3_ (JEOL, Tokyo, Japan, JMN-ECP400), and elemental analysis (J-Science Lab, Kyoto, Japan, Micro Corder JM10). Complete imidization for the PIs obtained via Route-C and Route-R was confirmed by the ^1^H-NMR spectra in DMSO-*d*_6_ (only for soluble systems) and/or transmission-mode FT-IR spectra using their thin films (~5 μm thick) with an intentionally roughened surface to suppress interference fringes. The melting points of the monomers were determined by differential scanning calorimetry (DSC, Rigaku, Tokyo, Japan, DSC 8231) from the endothermic peak temperatures in the DSC thermograms measured at a heating rate of 5 °C/min in a nitrogen atmosphere.

#### 2.2.2. Inherent Viscosities and Molecular Weights

As an index of polymer molecular weights, the reduced viscosities (*η*_red_) of PAAs and/or PIs were measured at a polymer content of 0.5 wt% at 30 °C on an Ostwald viscometer because it was difficult to determine the inherent viscosities (*η*_inh_) by the extrapolation to zero PAA concentration owing to a polyelectrolyte effect of PAAs. The measurements were conducted promptly after dilution of the as-polymerized PAA solutions to 0.5 wt% because dilution of PAA solutions to a low concentration (<1 wt%) often accelerates their molecular weight decrease. The high *η*_red_ values (> ~1.0 dL/g) can be empirically regarded as being PAAs or PIs with sufficiently high molecular weights for ensuring film-forming ability.

The number-($\bar{M}$_n_) and weight-average molecular weights ($\bar{M}$_w_) for highly soluble PIs in THF were measured by gel permeation chromatography (GPC) in THF at room temperature using a 0.05 wt% dilute solution after filtration with a PTFE-membrane filter (pore size: 0.1 μm). GPC was conducted on an HPLC system (JASCO, Tokyo, Japan, LC-2000 Plus) equipped with a GPC column (Resonac, Tokyo, Japan, Shodex, KF-806L) and an ultraviolet–visible detector at a wavelength of 300 or 254 nm (JASCO, Tokyo, Japan, UV-2075) at a flow rate of 1 mL/min. The calibration was performed using standard polystyrenes (Resonac, Tokyo, Japan, Shodex, SM-105). A typical GPC curve is shown in Figure S2.

#### 2.2.3. Linear Coefficients of Thermal Expansion (CTE)

The *XY*-direction CTEs as an index of thermal dimensional stability of the PI films were measured by thermomechanical analysis (TMA) using PI specimens (length: 20 mm; width: 5 mm; typical thickness: 20 μm; chuck-to-chuck distance: 15 mm) on a thermomechanical analyzer (Netzsch Japan, Yokohama, Japan, TMA 4000 or Rigaku, Tokyo, Japan, TMA 8311). TMA was conducted at a heating rate of 5 °C/min with a fixed load (0.5 g per unit film thickness in μm, i.e., a 10 g load for 20 μm thick specimens) in a dry nitrogen atmosphere. The CTE values were calculated from the slope of the TMA straight line in the range of 100–200 °C (glassy temperature region, *T* < *T*_g_) during the 2nd heating run ranging from 30 to 450 °C, before which the preliminary 1st run was conducted in the range of 30–150 °C to remove adsorbed water on the mounted specimens and subsequent cooling to 30 °C with a continuous dry N_2_ flow in the sealed TMA chamber.

To determine the CTE values accurately, residual strain of the film specimens must be removed in advance because it often causes film shrinkage during the TMA heating run. As-delaminated PI films after thermal imidization on the substrates usually include residual strain. This can be removed by careful annealing without the substrates while avoiding significant orientational relaxation and undesirable film deformation. The adequate annealing conditions and how to check the removal of residual strain are described in our previous paper [48].

#### 2.2.4. Glass Transition Temperatures (*T*_g_)

The physical (short-term) heat resistance, *T*_g_s of the PI films were measured by dynamic mechanical–thermal analysis (DMA) on a dynamic viscoelastic analyzer (TA Instruments Japan, Tokyo, Japan, DMA-Q800) or a thermomechanical analyzer (Netzsch Japan, Yokohama, Japan, TMA 4000). The storage modulus (*E*’) and loss modulus (*E*”) as a function of temperature were measured in the range of 30–450 °C at a heating rate of 5 °C/min in an N_2_ atmosphere under a sinusoidal strain frequency of 0.1 Hz (amplitude: 0.1%). The *T*_g_s were determined from the peak temperatures in the *E*” curve, unless otherwise noted.

The *T*_g_s were also measured by TMA from an inflection point in the TMA curve, which is determined from an intersection of two tangential lines. The TMA-based *T*_g_s were essentially equivalent to the DMA-based values (specifically, the former is often 5–20 °C higher than the latter).

#### 2.2.5. Chemical (Long-Term) Heat Resistance

The 5% weight loss temperatures (*T*_d_^5^) as an index of thermal and thermo-oxidative stability of the PI films were measured by thermogravimetric analysis (TGA) at a heating rate of 10 °C/min in a dry N_2_ and/or air atmosphere on a thermo-balance (Netzsch Japan, Yokohama, Japan, TG-DTA2000S or Rigaku, Tokyo, Japan, TG8121). A small weight loss due to desorption of adsorbed water at 100 °C during the TGA heating runs was compensated by off-setting at 150 °C to 0% weight loss for the data analysis.

#### 2.2.6. Optical Transparency

The optical transparency of the PI films (typical thickness: 20 μm) was evaluated from the light transmittance at 400 nm (*T*_400_), cut-off wavelength (*λ*_0_), yellowness index (YI), total light transmittance (*T*_tot_), and haze. The optical transmission spectra were measured in the wavelength (*λ*) range from 200 to 800 nm to determine the *T*_400_, *λ*_0_, and YI on an ultraviolet–visible spectrophotometer (JASCO, Tokyo, Japan, V-530). The YI values were calculated under a standard illuminant of D65 and a standard observer function of 2° (ASTM E 313) using a color calculation software (JASCO, Tokyo, Japan) from the following relationship:

YI = 100 × (1.2985*x* − 1.1335*z*)/*y* (1)

where *x*, *y*, and *z* are the CIE tristimulus values. YI becomes zero for an ideal white/transparent sample. The *T*_tot_ (JIS K 7361-1) and diffuse transmittance (*T*_diff_, JIS K 7136) of the PI films were measured on a double-beam haze meter equipped with an integrating sphere (Nippon Denshoku Industries, Tokyo, Japan, NDH 4000). The haze of the PI films was calculated from the following relationship:

Haze = (*T*_diff_/*T*_tot_) × 100 (2)

#### 2.2.7. Birefringence

The thickness-direction birefringence (Δ*n*_th_) of the PI films was measured at 589.3 nm (Na-lamp, *D*-line) on an Abbe refractometer (ATAGO, Tokyo, Japan, 4T, *n*_D_ range: 1.47–1.87) equipped with a polarizer, Na-lamp, and white reflector using a test piece (*n*_D_ = 1.92) and contact liquid (methylene iodide, *n*_D_ = 1.74). This study does not address the wavelength dependence of refractive indices and birefringence. The Δ*n*_th_ values were calculated from the following relationship:

Δ*n*_th_ = *n*_xy_ − *n*_z_ (3)

where *n*_xy_ and *n*_z_ denote the *XY*-direction (in-plane) and *Z*-direction (out-of-plane) refractive indices of the PI films. In general, Δ*n*_th_ includes the orientation and stress birefringence. However, in this study, the contribution of the latter can be considered very small because the residual stress in the film specimens was thoroughly removed by annealing at adequate temperatures without the substrates. Therefore, Δ*n*_th_ is closely related to the extent of polymer chain alignment along the *XY*-direction (in-plane orientation) [59,60].

The optically estimated dielectric constants (*ε*_opt_) for the PI films were calculated from the following empirical relationship [21]:

*ε*_opt_ = 1.1 × *n*_av_^2^ (4)

here *n*_av_ denotes the average refractive indices [*n*_av_ = (2*n*_xy_ + *n*_z_)/3] of the PI films.

#### 2.2.8. Mechanical Properties

The mechanical properties [tensile modulus (*E*), tensile strength (*σ*_b_), and elongation at break (*ε*_b_)] of the PI films (specimen length: 30 mm; width: 3 mm; typical thickness: 20 μm; and number of valid specimens > 10) were evaluated at a cross-head speed of 8 mm/min at room temperature on a mechanical testing machine (A & D, Tokyo, Japan, Tensilon UTM-II). The raw data were analyzed using a data processing program (Softbrain, Tokyo, Japan, UtpsAcS Ver. 4.09).

#### 2.2.9. Water Uptake

The water uptake (*W*_A_) of the PI films was determined from the following relationship:

*W*_A_ = [(*W* − *W*_0_)/*W*_0_] × 100 (5)

where *W*_0_ is the weight of a vacuum-dried film sample at 50 °C for 24 h, and *W* is the weight of the film immersed in water at 23 °C for 24 h and carefully blotted dry with tissue paper. KAPTON^®^ H film (Toray Kapton Co., Tokyo, Japan, thickness: 50 μm) was used as a reference sample (*W*_A_ = 2.7% [7]) in each weighing. The static electricity of the specimens was discharged using a static elimination gun (Milty, Bishop’s Stortford, UK, Zerostat 3) before each weighing.

#### 2.2.10. Liquid Crystallinity

The liquid-crystal phase of the vertical-alignment-type diamines was observed on a polarizing optical microscope (POM, Olympus, Tokyo, Japan, BX51) equipped with a digital camera (Nikon, Tokyo, Japan, Coolpix 950) and a temperature-controllable hot stage (Mettler Toledo, Tokyo, Japan, FP82HT hot stage and FP 90 central processor).

## 3. Results and Discussion

### 3.1. Fundamental Demands and Selection of Pristine PI Structure to Be Modified

The fundamental demands in this study are as follows:

(i) Smooth polymerization without salt formation;
(ii) Sufficiently high polymerization reactivity to obtain polymers with sufficiently high *M*_w_;
(iii) Extremely high heat resistance;
(iv) Non-coloration;
(v) Film-forming ability and toughness;
(vi) Low CTE;
(vii) Low *Z*-direction birefringence (Δ*n*_th_);
(viii) Excellent solubility for solution casting from stable homogeneous PI solutions.

**Scheme 25:**
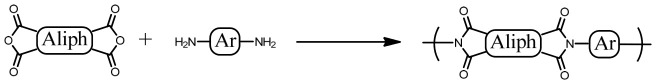
Semi-cycloaliphatic PI systems (B-type) derived from cycloaliphatic TCDAs with aromatic diamines.

As mentioned in Section 1, Introduction, based on demands (i), (iii), and (iv), our target materials were narrowed down to semi-cycloaliphatic PIs (B-type), as shown in Scheme 25.

**Figure 17:**
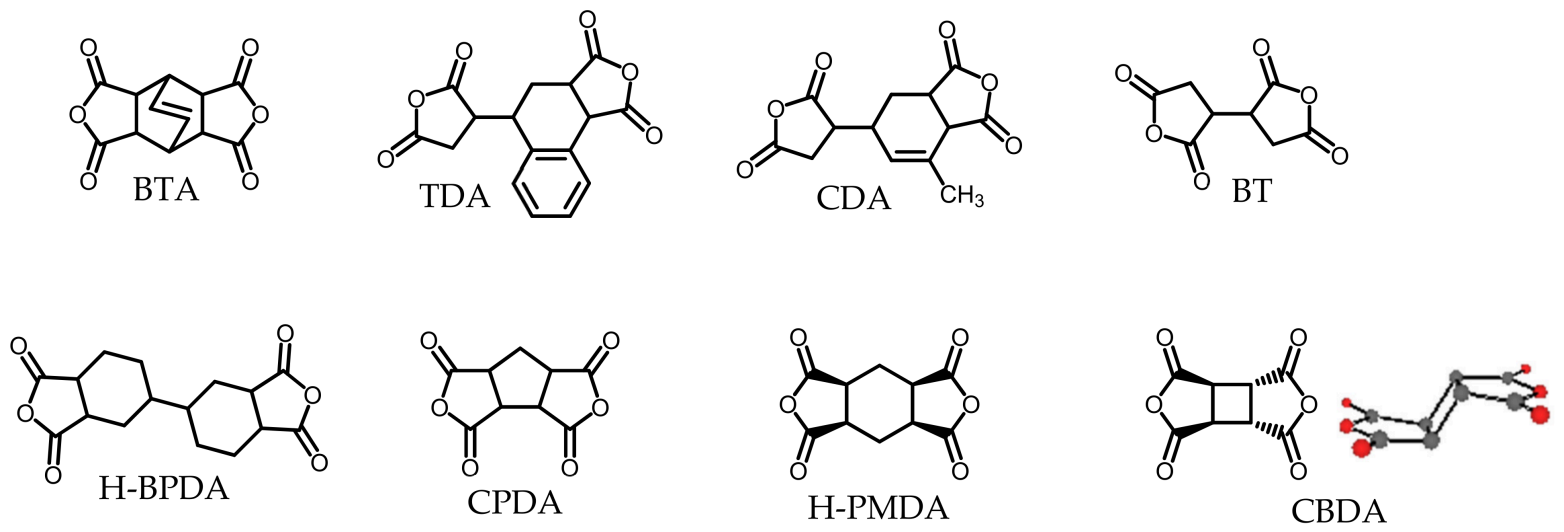
Molecular structures and abbreviations of common cycloaliphatic tetracarboxylic dianhydrides and the steric structure of CBDA.

However, commercially available cycloaliphatic TCDAs (Figure 17) are not so abundant, suggesting their limited modification flexibility. Furthermore, many of these cycloaliphatic TCDAs do not always have sufficiently high polyaddition reactivity with aromatic diamines for obtaining PAAs and PIs with sufficiently high molecular weights ((demand (ii)), and consequently, sufficient film-forming ability and toughness (demand (v)). Highly reactive cycloaliphatic TCDAs satisfying demand (v) are virtually limited to 1,2,3,4-cyclobutanetetracarboxylic dianhydride (CBDA) [32,37,39]. Moreover, low-CTE characteristics (demand (vi)) originate from a great extent of main-chain in-plane orientation induced during thermal imidization [61,62,63,64], which has a strong trend to increase as their structural linearity/rigidity increases [49,50,51]. In semi-cycloaliphatic PIs (B-type), the steric structures of cycloaliphatic TCDAs are a crucial factor in determining whether a low CTE is achieved [18,25,26,41,47,48]. In this respect, CBDA is an unusual cycloaliphatic TCDA with relatively high structural linearity/rigidity (a crank-shaft-like structure [18], Figure 17), suggesting a high possibility of reducing the CTE [32,37]. This structural feature also contributes to the enhancement of the *T*_g_ [47]. Thus, to achieve demands (i)–(vi), there was no alternative to CBDA as the optimum cycloaliphatic TCDA for the purposes of the present study.

**Scheme 26:**
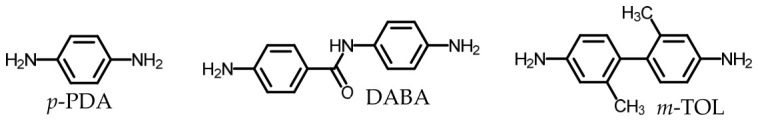
Molecular structures of *p*-phenylenediamine (*p*-PDA), 4,4′-diaminobenzanilide (DABA), and *m*-tolidine (*m*-TOL).

The aromatic diamines used with CBDA must also have linear/rigid structures to ensure a low CTE, with typical examples shown in Scheme 26.

**Scheme 27:**
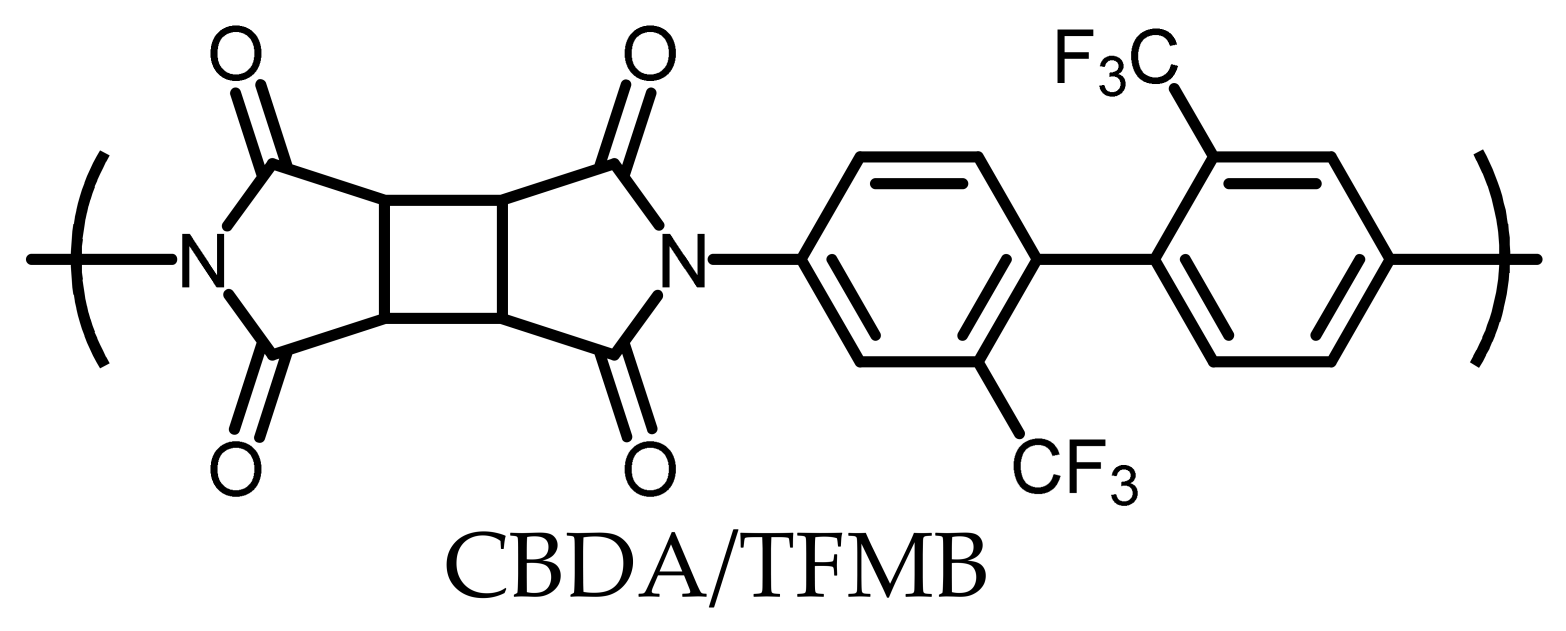
Structure of selected pristine PI system.

However, the use of these rigid diamines often significantly deteriorates the optical transparency of the resulting PI films because of the difficulty in removing unknown colored impurities contained in them. Therefore, TFMB with a rigid structure and complete non-coloration was exclusively selected to achieve our goal. Thus, in this study, we focused on CBDA/TFMB polyimide (Scheme 27) as the optimal pristine system to be modified.

### 3.2. Impact of the Polymerization/PI Film Preparation Route

Although Route-C is often more advantageous for improving PI film transparency and reducing the CTE than Route-T, it is only applicable to highly soluble PI systems [26,32,65]. The benefit of Route-C on transparency is attributed to its milder film preparation condition without a thermal imidization process and the end-capping protection of the thermally unstable terminal amino groups by Ac_2_O during chemical imidization. Route-R also has similar advantages compared to Route-T. More specifically, Route-C is slightly advantageous over Route-R in terms of film transparency because a catalyst residue (1-ethylpiperidine) in Route-R is responsible for only slight film coloration in some cases [39]. Thus, the effect of the polymerization/film preparation route on the film transparency is as follows: Route-C ≥ Route-R >> Route-T (Table 2), as long as undesirable solvents (e.g., NMP) for solution casting are not used, as discussed later.

In systems with high polyaddition reactivity, the advantage for lowering CTE is as follows: Route-R (or Route-C) > Route-T (Table 2). The superiority of Route-R and Route-C is based on unique self-orientation behavior during the solution casting using PI solutions [26,39,65]. This unique behavior is usually not observed in common polymer systems, as evident by our observation that an NMP-cast poly(ether sulfone) film shows a three-dimensionally random chain orientation distribution with zero ∆*n*_xy_ and an almost zero ∆*n*_th_ (0.0001), corresponding to its high CTE (65 ppm/K). CTE is also prone to decrease as the molecular weights of PIs increase [26]. From this perspective, the advantage is as follows in some cases: Route-R > Route-C. Moreover, when the polyaddition reactivity of the monomers used is not sufficiently high to obtain PAAs with sufficiently high molecular weights, the process superiority in terms of film-forming ability (or toughness) is as follows: Route-R >> Route-C ≥ Route-T (Table 2).

Unfortunately, the selected pristine system to be modified (CBDA/TFMB) was not compatible with Route-C and Route-R [66] (Figure S3). Therefore, the success of this study depends on whether the solubility of CBDA/TFMB can be dramatically improved by copolymerization with adequate modifiers, and consequently, Route-C or Route-R becomes applicable.

### 3.3. Trade-Off Between Low CTE and Low Δn_th_

**Figure 18:**
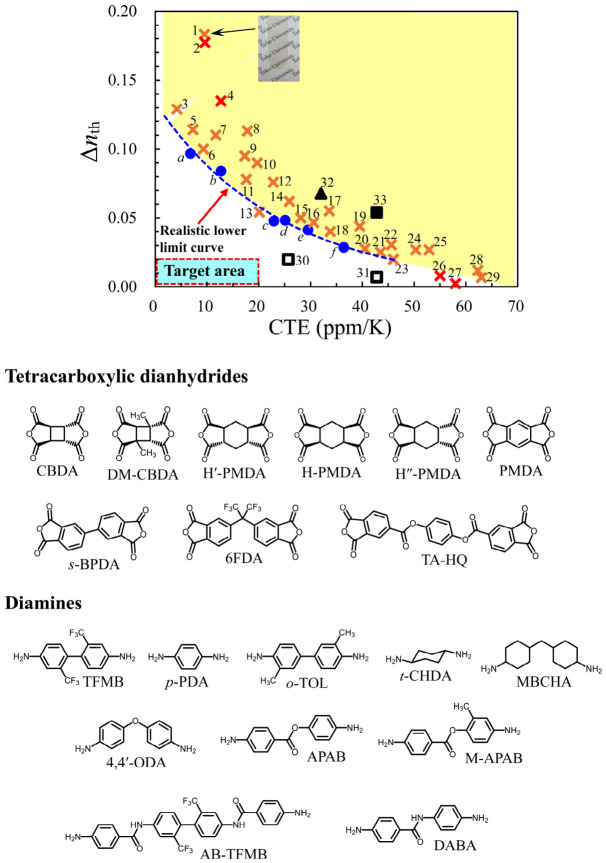
The CTE–Δ*n*_th_ correlation for the CBDA/TFMB polyimide films (**●**) prepared under different conditions from the PAA film dried at 60 °C for 2 h, followed by (**code-*a***) soaking of the PAA cast film formed on a glass substrate in Ac_2_O/pyridine at room temperature for 24 h, washing with *n*-hexane, vacuum-drying at 200 °C/1 h on the substrate, and subsequent annealing at 330 °C/1 h without substrate under vacuum; (***b***) soaking of the PAA cast film on the glass substrate in Ac_2_O/pyridine at room temperature for 24 h, washing with *n*-hexane, and vacuum-drying at 200 °C/1 h without the substrate; (***c***) standard route (T)—thermal imidization of the PAA cast film at 200 °C/1 h + 300 °C/1 h (<*T*_g_) on the substrate under vacuum, and subsequent annealing at 330 °C/1 h without substrate under vacuum; (***d***) Route-T using a copper substrate; (***e***) thermal imidization of the PAA cast film at 200 °C/1 h + 360 °C/1 h (>*T*_g_) on the substrate under vacuum, and subsequent annealing at 330 °C/1 h without substrate under vacuum; and (***f***) thermal imidization at 200 °C/1 h + 300 °C/1 h without the substrate. Our previous data for other colorless PIs (×, □) are also plotted: (**code-1**) *s*-BPDA/*t*-CHDA(T) [66], (**2**) PMDA/*t*-CHDA(T) [67], (**3**) 6FDA(20);CBDA(80)/AB-TFMB(C) [37], (**4**) TA-HQ/*t*-CHDA(T) [73], (**5**) 6FDA(30);CBDA(70)/AB-TFMB(C) [37], (**6**) CBDA/DABA(T) [25], (**7**) 6FDA(40);CBDA(60)/AB-TFMB(C) [37], (**8**) CBDA/APAB(T) [25], (**9**) 6FDA(50);CBDA(50)/AB-TFMB(C) [37], (**10**) CBDA/M-APAB(T) [25], (**11**) DM-CBDA/DABA(T) [25], (**12**) 6FDA(70);CBDA(30)/AB-TFMB(C) [37], (**13**) CBDA/*p*-PDA(T) [25], (**14**) H-PMDA/AB-TFMB(R) [39], (**15**) DM-CBDA/TFMB(T) [25], (**16**) H′-PMDA/DABA(T) [26], (**17**) 6FDA/AB-TFMB(C) [37], (**18**) *s*-BPDA/TFMB(T) [66], (**19**) H′-PMDA/TFMB(C), (**20**) H″-PMDA/DABA(T) [24], (**21**) H″-PMDA/TFMB(C) [24], (**22**) H″-PMDA/*o*-TOL(C) [24], (**23**) H′-PMDA/TFMB(C) [26], (**24**) CBDA/4,4′-ODA(T) [37], (**25**) 6FDA/TFMB (C) [65], (**26**) PMDA/MBCHA(T) [67], (**27**) 6FDA/*t*-CHDA(T) [66], (**28**) *s*-BPDA/MBCHA(T) [68], (**29**) CBDA/MBCHA(T) [67], (**30**) CBDA/*t*-CHDA(T) [67], (**31**) DM-CBDA/*t*-CHDA(T) [25] for wholly cycloaliphatic PIs (**□**). For comparison, the data for wholly aromatic PMDA/4,4′-ODA(T) (▲, ■) prepared under different conditions are plotted in this diagram: **code-32** [61] and **code-33** [68]. The inserted image is the appearance of the *s*-BPDA/*t*-CHDA PI film(T).

The addition of demand (vii), low Δ*n*_th_, to demands (i)–(vi) is overwhelmingly challenging to achieve owing to the inherent difficulty in simultaneously achieving low CTE and low Δ*n*_th_, as mentioned previously (Figure 1). This trade-off is evident from the CTE–Δ*n*_th_ correlation diagram of various transparent PIs that we have investigated so far (Figure 18). For example, the colorless films of rigid PIs, i.e., *s*-BPDA/*t*-CHDA (*s*-BPDA = 3,3′,4,4′-biphenyltetracarboxylic dianhydride, *t*-CHDA = *trans*-1,4-cyclohexanediamine, **#1**, inset photograph in Figure 18 [66]) and PMDA/*t*-CHDA (PMDA = pyromellitic dianhydride, **#2** [67]), show very low CTE values (10 ppm/K) and concomitantly increased Δ*n*_th_ (>0.1). Conversely, their counterparts using a flexible cycloaliphatic diamine (4,4′-methylenebis(cyclohexylamine), mixture of isomers, MBCHA), i.e., *s*-BPDA/MBCHA, **#28** [68] and PMDA/MBCHA, **#26** [67]), showed high CTE values (>50 ppm/K), which are due to poor in-plane orientation based on their distorted main-chain structures, and concomitantly, significantly low Δ*n*_th_ values.

First, we narrowed down an optimum pristine system with the potential to achieve our goals via various modifications by understanding the structure–Δ*n*_th_ relationship. The intrinsic birefringence (Δ*n*_0_) of polymers, which is determined only by the molecular structures, is expressed based on the Lorentz–Lorenz equation as follows [69,70]:

(6) $$\Delta n_{0}\;=\;\frac{2}{9}\pi \;\frac{({\bar{n}}^{2}\;+\;{2)}^{2}}{\bar{n}}\frac{\rho N_{A}}{M_{0}}\Delta \alpha$$

where *ρ* and $\bar{n}$ denote the density and average refractive index of the film specimens, respectively, *N*_A_ is Avogadro constant, and *M*_0_ and Δ*α* represent the molar mass and polarization anisotropy of the polymer repeating units. In in-plane isotropic (negative-C) films, such as non-stretched PI cast films discussed in this study, birefringence (Δ*n*) is expressed by defining the *Z* (thickness)-direction as an optical axis in the following equation:

Δ*n* = *n*_z_ − *n*_xy_ = *f* Δ*n*_0_ (7)

where *f* represents the Hermans orientation function: *f* = <3cos^2^*θ* − 1>/2 (*θ*: statistical angle between the polymer main chains and the optical axis (*Z*-direction)). The *f* ranges from –0.5 at *θ* = 90° [perfect main-chain orientation in the *XY*-direction (perfect in-plane orientation) to 1 at *θ* = 0° (perfect main-chain orientation in the *Z*-direction, i.e., perfect vertical alignment). Therefore, from Equations (3) and (7), Δ*n*_th_ is expressed as follows:

Δ*n*_th_ = −*f* Δ*n*_0_ (8)

For example, using Δ*n*_0_ = 0.66 of the *s*-BPDA/*p*-PDA (*p*-PDA = *p*-phenylenediamine) PI film, which was estimated in our previous paper [71], a virtual film of *s*-BPDA/*p*-PDA with perfect in-plane (ideal 2D) orientation is presumed to have Δ*n*_th_ = –(–0.5) × 0.66 = 0.33 from the simple calculation based on Equation (8). The actual *s*-BPDA/*p*-PDA PI film exhibited a very high Δ*n*_th_ value (0.24 [72]) close to that of the ideal 2D orientation (Δ*n*_th_ = 0.33), corresponding to the extremely low CTE (5–10 ppm/K) arising from significant in-plane orientation [49,50,51,61,62].

In contrast to the *s*-BPDA/*p*-PDA system, the only way to suppress an increase in Δ*n*_th_ while maintaining a low CTE is to reduce the structure-originating Δ*n*_0_ value. Thus, Equation (6) indicates that chemical structures with lower $\bar{n}$ and Δ*α* are valuable as those of the pristine PI system. Considering that the electronic polarization of PIs decreases in the following order: wholly aromatic PIs > semi-cycloaliphatic PIs (A-type) ≥ semi-cycloaliphatic PIs (B-type) > wholly cycloaliphatic PIs, wholly cycloaliphatic PIs without π electrons are, in principle, the best choice as the pristine system. Poorly polarized fluorine-containing systems are also preferable for similar reasons. Indeed, the plots (□) of wholly cycloaliphatic PIs in Figure 18 are positioned in a much-lower-Δ*n*_th_ region away from the other plots. In particular, the wholly cycloaliphatic CBDA/*t*-CHDA system (□, **#30** in Figure 18), consisting of a relatively linear/rigid structure, exhibited relatively low CTE (25.7 ppm/K) and Δ*n*_th_ (0.0199) [67]. In contrast, the plots of π-electron-rich wholly aromatic PMDA/4,4′-ODA (4,4′-ODA = 4,4′-oxydianiline, ▲, ■ in Figure 18) were located in a higher-Δ*n*_th_ region than the other plots, corresponding to the above descriptions.

Despite the above-mentioned superiority of the CBDA/*t*-CHDA system, this PI was excluded as a candidate for the pristine PIs owing to the manufacturing complexity to obtain a flexible colorless PI film: a controlled fraction of silylation of *t*-CHDA, polyaddition in a mixed solvent containing toxic hexamethylphosphoric triamide, and solvent extraction (removal) from the PAA cast film before thermal imidization [67].

**Scheme 28:**
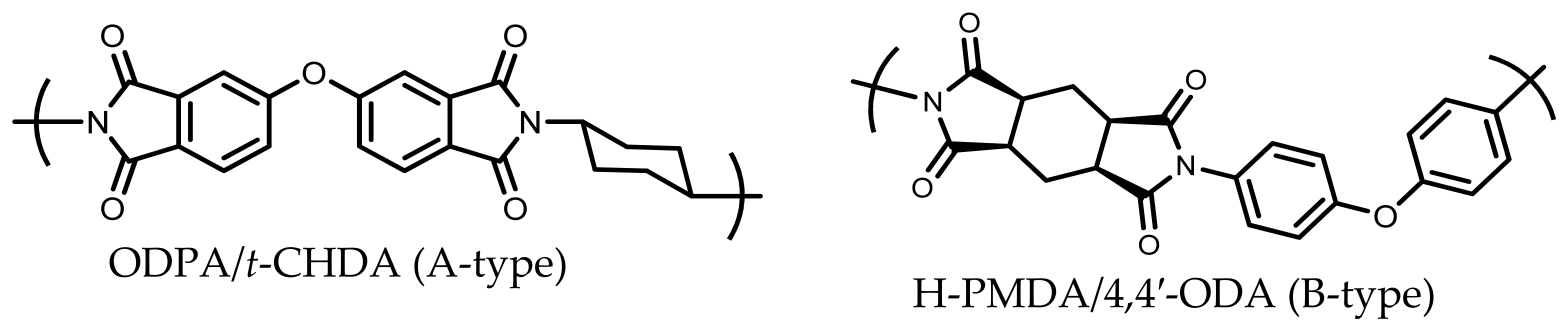
Structures of comparative semi-cycloaliphatic PIs (A-type and B-type).

The above-mentioned order of electronic polarization—semi-cycloaliphatic PIs (A-type) ≥ semi-cycloaliphatic PIs (B-type)—is based on the following facts: in A-type, the imide C=O groups participate in electronic conjugation with the central aromatic groups, whereas in B-type, such conjugation extension in the aromatic unit in the diamine parts is minor [10,74], as suggested by the comparison of *n*_av_ between the biphenyl ether-containing systems: *n*_av_ = 1.634 for ODPA/*t*-CHDA (ODPA = 4,4′-oxydiphthalic anhydride, A-type) and *n*_av_ = 1.622 for H-PMDA/4,4′-ODA (H-PMDA = hydrogenated PMDA, B-type) (Scheme 28).

Among semi-cycloaliphatic PIs (B-type), the CBDA/TFMB PI film prepared via Route-T under the standard thermal conditions (**code-*c*** in Figure 18) maintained a relatively low CTE and Δ*n*_th_ in addition to high optical transparency, as mentioned previously. We subsequently prepared PI films of this system under various conditions (see the caption of Figure 18). The obtained plots (●) formed a clear CTE–Δ*n*_th_ curve without significant data scattering. This clear CTE–Δ*n*_th_ correlation also suggests that both experimental parameters were accurately determined, and the CTE values are primarily governed by the degree of in-plane orientation. The CTE–Δ*n*_th_ curve obtained in the CBDA/TFMB system seems to represent a “realistic lower boundary” because many other plots (except for the plots of the wholly cycloaliphatic PIs (□)) are located above this curve (yellow-shaded region). As mentione above, the structure-dependent electronic polarization decreases in the following order—wholly aromatic PIs > semi-cycloaliphatic PIs (A-type) > B-type > wholly cycloaliphatic PIs. Therefore, the CTE–Δ*n*_th_ curve is presumed to shift parallel toward the lower Δ*n*_th_ direction while maintaining a similar shape. Indeed, it appears that the plots of the wholly cycloaliphatic PIs (□) form another CTE–Δ*n*_th_ curve that is significantly shifted downward, although there are only a few data points.

In this study, we established a target area satisfying both CTE ≤ 20 ppm/K and Δ*n*_th_ ≤ 0.02 as a temporal goal, as shown in Figure 18, and undertook various chemical modifications of CBDA/TFMB to exceed the lower limit curve toward the target area without the help of wholly cycloaliphatic PIs and fillers.

### 3.4. Attempt to Modify CBDA/TFMB Using a Liquid-Crystalline Diamine

We first attempted to modify CBDA/TFMB with 35DAB-BPC_12_ (Scheme 4), which is expected to vertically align. This diamine monomer showed complex thermal-transition behavior, as shown in its DSC curves during the heating and cooling processes (Figure S4a). A thermotropic liquid-crystal (LC) phase was observed in the temperature range of 172.8–178.4 °C during the heating process and 176.4–165.5 °C during the cooling process, as shown in the POM photograph taken at 172.8 °C in the heating process (Figure S4b). The results indicate that the *p*-biphenylene benzoate unit in this diamine can behave as a mesogenic unit. From the appearance of the LC phase, it is presumed that the biphenyl benzoate units are extended with a rod-like shape and densely aggregated (Figure S4c), as is common in nematic liquid crystals.

When this LC diamine was partially incorporated into CBDA/TFMB, the PI main chains align in the *XY*-direction, and concomitantly, the *n*-dodecyl-substituted mesogenic units are expected to extend in the *Z*-direction (homeotropic alignment) to minimize the molecular contact with PI chains owing to expected poor miscibility between the *n*-dodecyl group and PI chains (Figure S5). Due to the high polarization anisotropy (∆*α* = *α*_//_ − *α*_⊥,_ *α*_//_: long-axis polarization; *α*_⊥_: short-axis polarization) of the *p*-biphenylene benzoate side group [69], this side group, if vertically aligned, is expected to negate the *XY*-direction polarization due to the main-chain in-plane orientation, and consequently, reduce the Δ*n*_th_.

**Figure 19:**
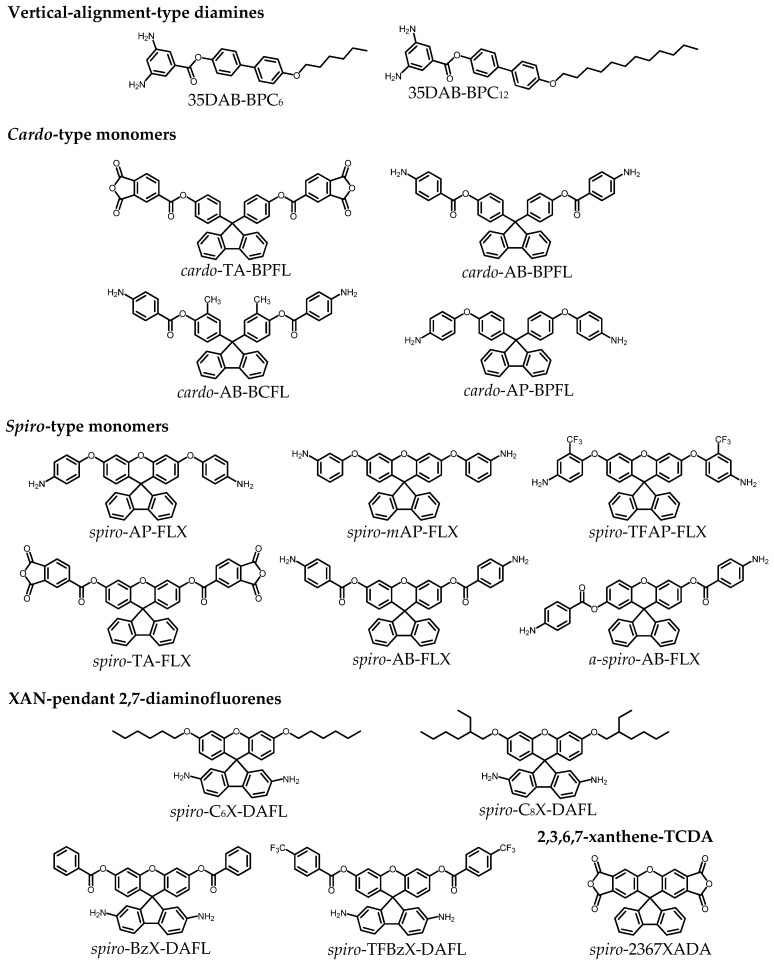
Structures and abbreviations of the modifiers used in this study.

**Figure 20:**
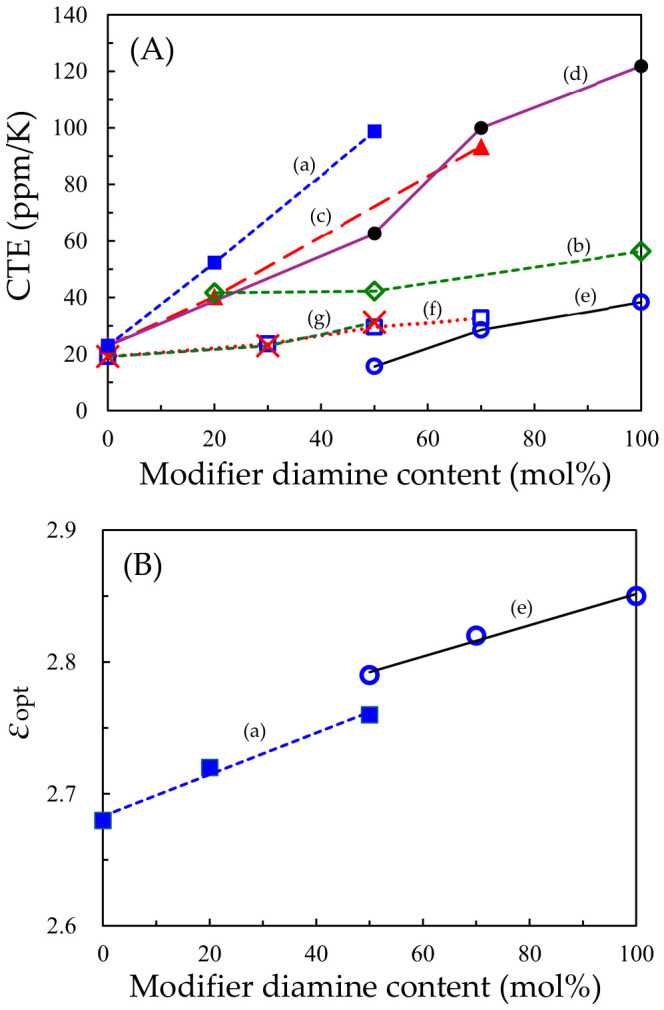
Impacts of the modifier diamine content on CTE (**A**) and *ε*_opt_ (**B**) for the PIs using different modifier diamines: (a) 35DAB-BPC_12_ (■, **#0T**, **#1T**, **#2T**), (b) *spiro*-TFAP-FLX (**◊, #25R**, **#26C**, **#27C**), (c) *spiro*-C_6_X-DAFL (▲, **#0T**, **#44T**, **#45T**), (d) *spiro*-C_8_X-DAFL (●, **#0T**, **#47T**, **#48T**, **#49T**), (e) *spiro*-TFBzX-DAFL (**○**, **#50C**, **#51C**, **#52C**), (f) *spiro*-TFBzX-DAFL (**□**, **#55R**, **#56R**, **#57R**, **#58R**), (g) *spiro*-BzX-DAFL (**×**, **#55R**, **#62R**, **#63R**).

The results of the polymerization and film properties (Route-T) of CBDA/TFMB modified with 35DAB-BPC_12_ (Figure 19) are summarized in Table 3. In the copolymer with 20 mol% 35DAB-BPC_12_ (**#1T**), no appreciable deterioration of the optical transparency was observed compared to the pristine system (**#0T**), as shown in the photograph of the film (**#1T**) at the bottom of Table 3. However, the transparency significantly decreased at 50 mol% 35DAB-BPC_12_ (YI = 8.57). This coloration is probably due to a trace amount of a colored product generated from partial thermal decomposition of the less thermally stable *n*-dodecyl group during thermal imidization. As the 35DAB-BPC_12_ content increases when going from **#0T** to **#1T** and **#2T**, there was no significant decrease in the *T*_g_, whereas the CTE abruptly increased (CTE = 98.7 ppm/K, **#2T**), as plotted in Figure 20A(a). This significant increase in CTE is a crucial problem, as low CTE is essential in this study. When going from **#0T** to **#1T** and **#2T**, the *ε*_opt_ increased almost linearly with increasing 35DAB-BPC_12_ content, corresponding to the increase in the π electrons originating from the aromatic units of this modifier (Figure 20B(a)). This reasonable linear increase in *ε*_opt_ supports the experimental accuracy for the refractive indices (*n*_xy_ and *n*_z_), ∆*n*_th_, and *n*_av_ of these films, because *ε*_opt_ is calculated using the raw data of the refractive indices. In other modifier systems, similar reasonable linear increases in *ε*_opt_ were observed with increasing modifier contents (Figure 20B(e)), as discussed later.

**Table 3:** Polymerization results and film properties of CBDA/TFMB- and H′-PMDA/TFMB-based PIs modified using 35DAB-BPC_12_ and related systems. The photographs at the bottom of this table show the appearance of selected PI films (**#0T, #1T**, **#3C**, and **#4C**).

No.	TCDA (mol%)	Diamine (mol%)	*η*_red_ (dL/g)	Cast ^c^	*T*_400_ (%)	YI	*λ*_0_ (nm)	*T*_tot_ (%)	Haze (%)	Δ*n*_th_	*ε*_opt_	*T*_g_ (°C)	CTE (ppm/K)	*T*_d_^5^ (N_2_) (°C)	*T*_d_^5^ (air) (°C)
0T	CBDA	TFMB	1.63 ^a^	---	84.2	3.97	302	89.8	0.87	0.0477	2.68	345	22.9	444	432
1T	CBDA	TFMB (80) 35DAB-BPC_12_ (20)	1.56 ^a^	---	84.8	1.74	328	90.2	1.91	0.0235	2.72	311	52.3	397	384
2T	CBDA	TFMB (50) 35DAB-BPC_12_ (50)	1.28 ^a^	---	68.0	8.57	349	82.7	1.17	0.0097	2.76	324	98.7	409	383
3C	H′-PMDA	TFMB	3.28 ^a^ 1.35 ^b^	CPN 15 wt%	86.1	1.77	293	90.5	2.02	0.0439	2.63	337	39.5	502	443
4C	H′-PMDA	TFMB (80) 35DAB-BPC_12_ (20)	1.30 ^a^ 0.87 ^b^	CPN 20 wt%	83.0	2.00	330	90.3	2.78	0.0144	2.69	287	42.0	386	372
5C	H′-PMDA	TFMB (70) 35DAB-BPC_12_ (30)	1.58 ^a^ 1.04 ^b^	CPN 20 wt%	81.3	1.97	337	90.0	1.74	0.0205	2.70	284	46.9	389	368
6T	CBDA (50) H′-PMDA (50)	TFMB (50) 35DAB-BPC_12_ (50)	1.43 ^a^	---	74.8	5.00	344	89.0	1.02	0.0018	2.76	298	106.3	391	370
6C	CBDA (50) H′-PMDA (50)	TFMB (50) 35DAB-BPC_12_ (50)	1.43 ^a^ 0.88 ^b^	CPN 3 wt%	72.1	2.85	349	89.3	4.01	0.0073	2.81	272	83.3	378	374
7C	H′-PMDA	TFMB (80) 35DAB-BPC_6_ (20)	2.35 ^a^ 0.81 ^b^	CPN 20 wt%	85.1	--- ^d^	324	--- ^d^	--- ^d^	0.0266	2.68	344	54.3	445	399
8C	H′-PMDA	TFMB (70) 35DAB-BPC_6_ (30)	1.85 ^a^ 0.65 ^b^	CPN 20 wt%	83.9	--- ^d^	327	--- ^d^	--- ^d^	0.0240	2.70	337	61.4	422	415

^a^ Data for PAAs. ^b^ Data for PIs prepared via Route-C. ^c^ Homogeneous PI solutions (at room temperature) used for the solution casting process. The solutions were prepared upon warming as appropriate. ^d^ Unmeasured data. GPC data: $\bar{M}$_n_ = 7.28 × 10^4^, $\bar{M}$_w_ = 1.25 × 10^5^ for **#3C**.


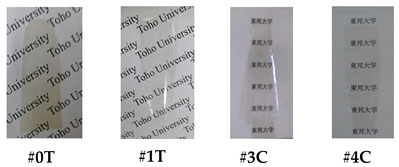


When replacing CBDA with H′-PMDA (an isomer of H-PMDA, Figure 13), the solubility of the resulting PIs was significantly improved (Table S2), and consequently, Route-C became applicable. No deterioration of the solubility with increasing 35DAB-BPC_12_ content occurred, and the originally excellent solubility was maintained (Table S2). An increase in film coloration was significantly suppressed even when the 35DAB-BPC_12_ content was increased from 0 (**#3C**) to 20 (**#4C**) and 30 mol% (**#5C**), as shown in the photographs of these films at the bottom of Table 3. In addition, the increase in CTE with increasing 35DAB-BPC_12_ content was very gradual.

The solubility of **#2T** was significantly improved by copolymerization with H′-PMDA. Consequently, the resulting system (**#6C**) became compatible with Route-C and concomitantly afforded a cast film with suppressed coloration (YI = 2.85).

**Figure 21:**
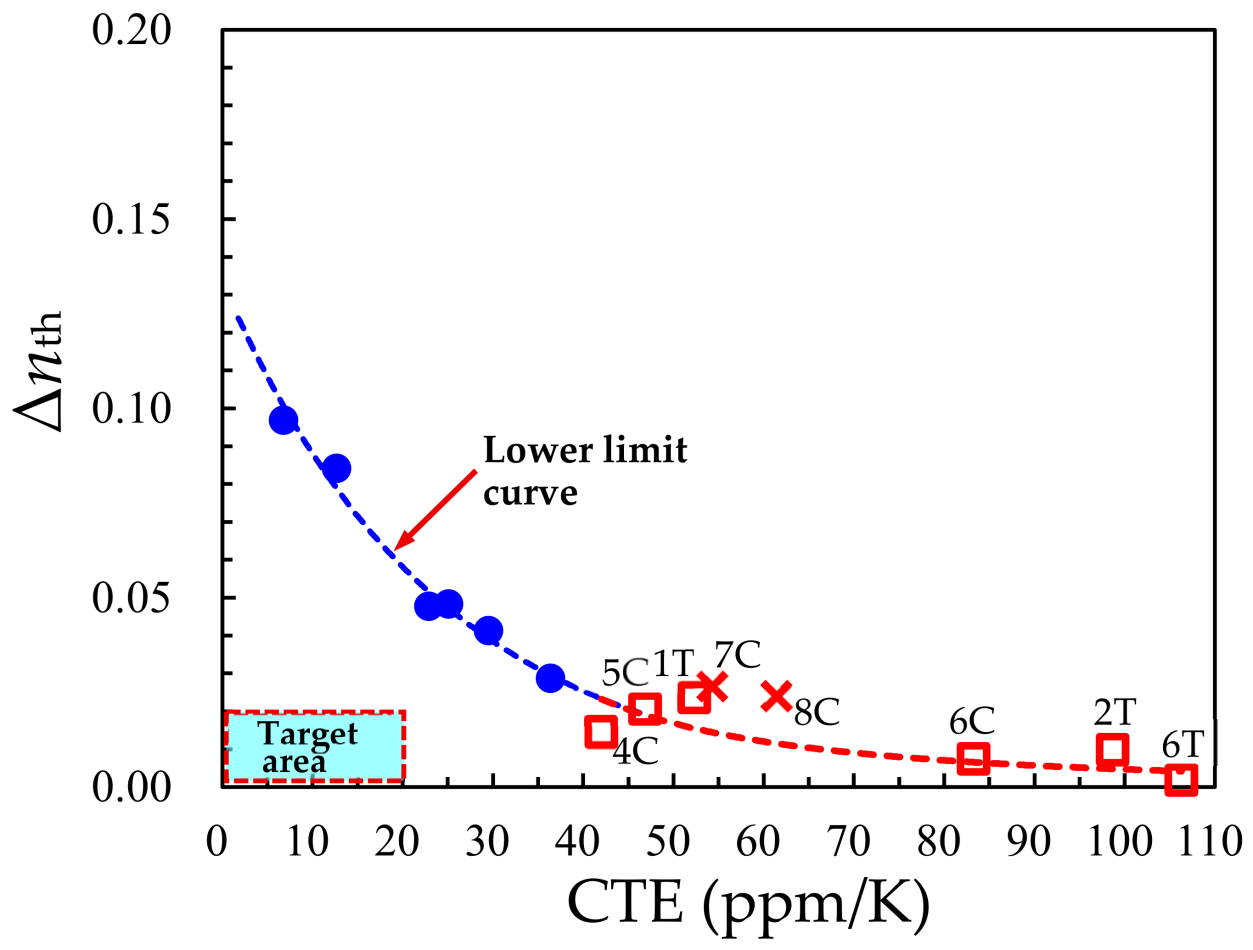
The CTE–Δ*n*_th_ relationship: (**□**) PIs modified with 35DAB-BPC_12_, (**×**) PIs modified with 35DAB-BPC_6_ (red dashed fitted curve), and (**●**) CBDA/TFMB PI films prepared under different conditions (blue dashed fitted curve: the lower limit curve). The inserted numbers represent the sample code (Table 3).

Figure 21 shows the CTE–Δ*n*_th_ correlation diagram for the 35DAB-BPC_n_-modified systems listed in Table 3. Most of these plots (□) were located on an extended curve (red dashed curve) of the lower boundary (blue dashed curve); thus, no prominent effect on overcoming the trade-off was observed. Furthermore, 35DAB-BPC_6_ (**×**), containing a shorter alkyl (*n*-hexyl) group, was also ineffective because these plots were positioned above the lower limit curve (blue dashed curve). Only **#4C** (**□**) slightly exceeded the lower limit curve, although its effect was not prominent. Thus, the modification effect of 35DAB-BPC_n_ was much smaller than expected. Possible reasons for this are as follows: (1) obstruction of the main-chain in-plane orientation by the *m*-phenylene diamine-based functional group of 35DAB-BPC_n_, and (2) unrealized vertical alignment of the modifier unit due to the collapse of the extended form of the modifier unit (Figure S6), which can be caused by obstruction of inter-mesogenic dense stacking (an essential condition for LC formation, Figure S4c) due to a “thinning-out” (dilution) effect of the modifier units as a result of copolymerization.

### 3.5. Effect of Cardo-Type Modifiers

**Figure 22:**
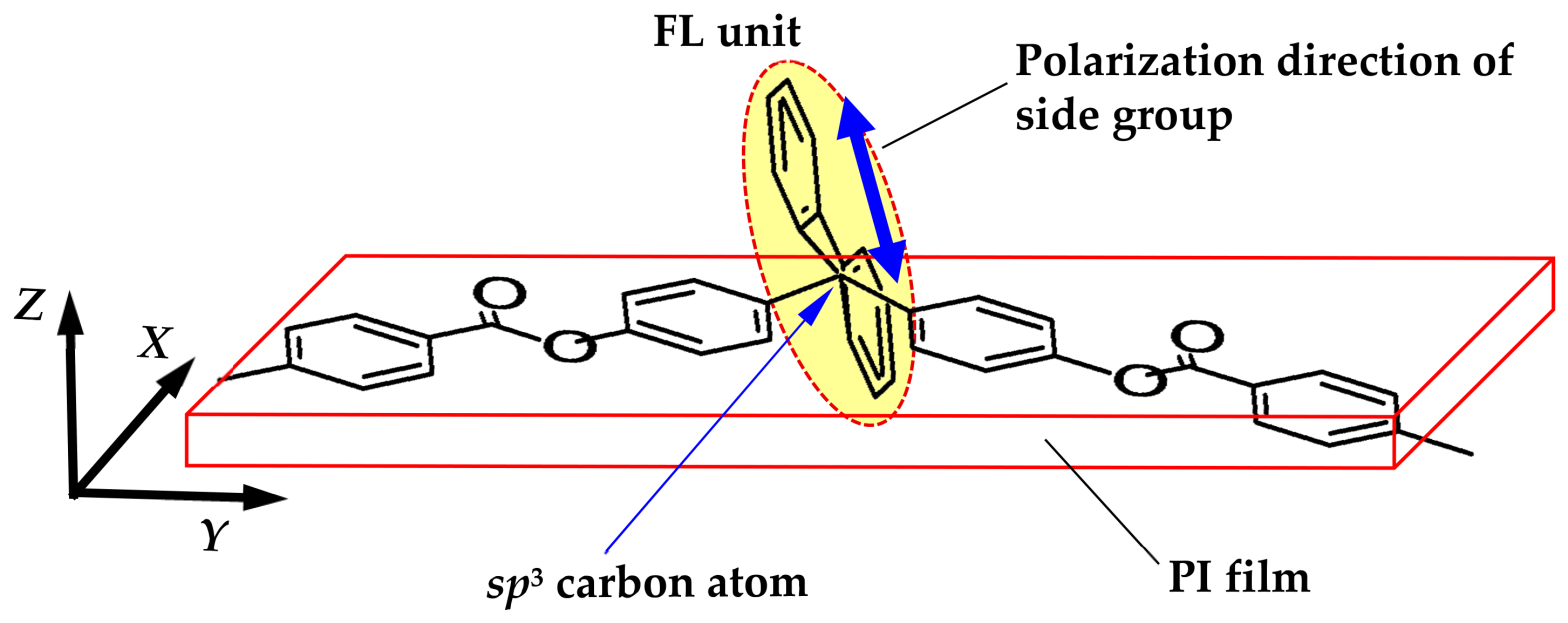
A schematic drawing for the expected *Z*-direction alignment of the fluorene (FL) side group connected to the PI main chains through the *sp*^3^ carbon atom in the *cardo*-type modifiers.

The results discussed in Section 3.4 suggested the importance of “perpendicularly and firmly” connecting the rotation-restricted rigid side groups to the PI main chains, while maintaining the *para*-configuration (or close to it) of the functional groups in the modifiers. From this point of view, we next attempted to modify CBDA/TFMB using *cardo*-type monomers, in which a rigid fluorene (FL) side group is firmly connected through the *sp*^3^ carbon atom to the 4,4′-methylenedianiline-based functional unit (Figure 22). Additionally, the bulky FL side group is expected to be effective for significantly improving the solubility of the resulting PIs, which plays a large role in achieving our goal, as discussed later, owing to the obstruction of dense inter-chain stacking [75,76,77].

We have previously investigated the basic properties of the PIs derived from *cardo*-TA-BPFL and *cardo*-AB-BPFL (Figure 19) [38]. However, at that time, we had no perspective on whether these monomers are effective in overcoming the trade-off between low CTE and low ∆*n*_th_. As a first attempt, we comprehensively re-examined the effectiveness of the systems using various *cardo*-type monomers by adding important optical properties.

Table 4 summarizes the polymerization results and film properties of the CBDA/TFMB-based PIs modified with a series of *cardo*-type monomers. The partial use of *cardo*-type monomers did not cause a significant decrease in *η*_red_, suggesting their sufficiently high polyaddition reactivity (*η*_red_ > 1.0 dL/g). Some of the modified systems exhibited significantly improved solubility; consequently, they became compatible with Route-C and soluble in various common solvents, including non-amide solvents (e.g., GBL, cyclopentanone (CPN)), as summarized in Table S2. The features of the systems using each *cardo*-type modifier are described below.

**Table 4:** Polymerization results and film properties of the CBDA/TFMB-based PIs modified using *cardo*-type monomers and related systems. The photographs at the bottom of this table show the appearance of selected PI films (**#10C** and **#13C**).

No.	TCDA (mol%)	Diamine (mol%)	*η*_red_ (dL/g)	Cast ^c^	*T*_400_ (%)	YI	*λ*_0_ (nm)	*T*_tot_ (%)	Haze (%)	Δ*n*_th_	*ε*_opt_	*T*_g_ (°C)	CTE (ppm/K)	*E* (GPa)	*ε*_b_ (%) ave max	*σ*_b_ (GPa)	*T*_d_^5^ (N_2_) (°C)	*T*_d_^5^ (air) (°C)
0T	CBDA	TFMB	1.63 ^a^	---	84.2	3.97	302	89.8	0.87	0.0477	2.68	345	22.9	6.57	5.2 8.0	0.19	444	432
9C	CBDA (70) *cardo*-TA-BPFL (30)	TFMB	1.53 ^a^ 1.63 ^b^	DMAc 9.9 wt%	70.0	3.06	350	88.7	4.58	0.0585	2.76	275	37.3	4.46	4.0 5.7	0.12	456	443
10C	CBDA (50) *cardo*-TA-BPFL (50)	TFMB	1.19 ^a^ 1.19 ^b^	GBL 9.8 wt%	65.9	3.58	358	88.6	1.36	0.0353	2.79	304	39.9	3.27	5.4 9.2	0.10	461	448
11C	*cardo*-TA-BPFL	TFMB	0.67 ^a^ 0.68 ^b^	GBL 9.1 wt%	55.9	2.71	364	88.6	1.32	0.0205	2.88	285	50.7	2.40	5.0 7.3	0.08	498	469
12T	CBDA	TFMB (80) *cardo*-AB-BPFL (20)	1.62 ^a^	---	84.1	3.12	318	89.4	0.79	0.0328	2.75	--- ^d^	--- ^d^	--- ^d^	--- ^d^	--- ^d^	417	419
13C	CBDA	TFMB (50) *cardo*-AB-BPFL (50)	1.72 ^a^ 1.57 ^b^	DMAc 8 wt%	84.4	1.81	318	88.8	3.65	0.0342	2.87	343	30.8	3.53	7.2 9.8	0.14	440	425
14C	CBDA	*cardo*-AB-BPFL	1.07 ^a^	---	Chemical imidization was unsuccessful due to precipitation													
15C	CBDA	TFMB (80) *cardo*-AB-BCFL (20)	1.35 ^a^	DMAc 9.9 wt%	79.9	5.80	321	89.3	0.80	0.0215	2.75	--- ^d^	--- ^d^	--- ^d^	--- ^d^	--- ^d^	436	427
16C	CBDA	TFMB (50) *cardo*-AB-BCFL (50)	1.37 ^a^ 1.90 ^b^	CPN 17 wt%	85.7	1.57	317	88.2	0.92	0.0349	2.85	335	36.6	4.52	7.6 14.4	0.16	441	421
17C	CBDA	*cardo*-AB-BCFL	2.00 ^a^ 1.03 ^b^	CPN 17 wt%	84.8	1.65	319	88.6	1.55	0.0176	2.95	326	42.8	3.83	7.9 11.0	0.14	413	413
18T	CBDA	TFMB (80) *cardo*-AP-BPFL (20)	1.62 ^a^	---	78.1	5.94	319	88.1	1.01	0.0288	2.76	--- ^d^	--- ^d^	--- ^d^	--- ^d^	--- ^d^	448	433

^a^ Data for PAAs. ^b^ Data for PIs prepared via Route-C. ^c^ Homogeneous PI solutions (at room temperature) used for solution casting. The solutions were prepared by heating the PI powder/solvent mixture for a short period as appropriate. ^d^ Data not available because of the difficulty of the measurements due to film brittleness. GPC data: $\bar{M}$_n_ = 3.26 × 10^4^ and $\bar{M}$_w_ = 8.14 × 10^4^ for **#10C**; $\bar{M}$_n_ = 1.98 × 10^4^ and $\bar{M}$_w_ = 5.04 × 10^4^ for **#11C**.


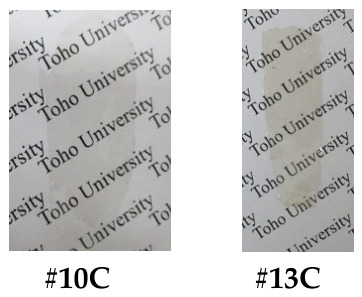


#### 3.5.1. *Cardo*-TA-BPFL-Modified Systems

The film properties of CBDA/TFMB-based systems modified with ester-linked *cardo*-TA-BPFL (Figure 19) are summarized in Table 4. The increase in the *cardo*-TA-BPFL content from 0 (**#0T**) to 30 (**#9C**), 50 (**#10C**), and 100 mol% (**#11C**) caused a gradual decrease in the optical transparency, as illustrated by the concomitant decreases in *T*_400_ and *T*_tot_. As a typical example, the appearance of the **#10C** film is shown at the bottom of this table. The observed gradual decrease in the transparency can likely be attributed to a gradual increase in weak intramolecular (through-bond) CT interactions [10,11,12] between the electron-accepting trimellitimide unit (originating from *cardo*-TA-BPFL) and the electron-donating *N*-aromatic unit (from TFMB) (Figure S7a). An increase in the *cardo*-TA-BPFL content also resulted in gradual increases in the thermal stability [*T*_d_^5^(N_2_)], thermo-oxidative stability [*T*_d_^5^(air)], and *ε*_opt_, owing to the concomitantly increased aromaticity. Moreover, as the *cardo*-TA-BPFL content increases, the ∆*n*_th_ decreased with an undesirable increase in the CTE. No clear toughening effect of *cardo*-TA-BPFL was observed, as suggested from the comparison of the *ε*_b_ of **#0T**, **#9C**, **#10C**, and #**11C**.

#### 3.5.2. *Cardo*-AB-BPFL-Modified Systems

The film properties of the systems modified with ester-linked *cardo*-AB-BPFL (Figure 19) are summarized in Table 4. In contrast to the *cardo*-TA-BPFL-modified systems, an increase in the *cardo*-AB-BPFL content did not deteriorate the film transparency, as indicated by their maintained high *T*_400_ values, owing to the absence of the afore-mentioned CT interactions in these PIs using only non-aromatic CBDA as the TCDA.

Unexpectedly, the copolymer film with 50 mol% *cardo*-AB-BPFL (**#13C**) exhibited higher transparency (lower YI) than the pristine system (homo CBDA/TFMB (**#0T**)). The appearance of this colorless PI film (**#13C**) is shown at the bottom of Table 4. This result reflects the fact that the **#13C** film was prepared via Route-C due to the significantly improved solubility, which is more advantageous for suppressing film coloration than Route-T, as described in Section 3.2. In addition, copolymerization with *cardo*-AB-BPFL did not cause a significant decrease in *T*_g_; for example, *T*_g_ = 343 °C at 50 mol% *cardo*-AB-BPFL (**#13C**) and 345 °C for CBDA/TFMB (**#0T**). The extremely high *T*_g_ of **#13C** reflects the prohibited own internal rotation of the bulky FL side group firmly connected through the *sp*^3^ carbon atom in addition to the restricted internal rotation around the ester groups due to a large sweep volume of the FL side group. The **#13C** film also showed a somewhat decreased ∆*n*_th_ compared to that of the pristine system (CBDA/TFMB, **#0T**), with an inevitably somewhat increased CTE. From the comparison of **#13C** and **#0T**, no clear toughening effect of c*ardo*-AB-BPFL was observed.

#### 3.5.3. *Cardo*-AB-BCFL-Modified Systems

CBDA/TFMB was also modified using dimethyl-substituted *cardo*-AB-BPFL, i.e., *cardo*-AB-BCFL (Figure 19). The incorporation of dimethyl groups into *cardo*-AB-BPFL was effective for further improving the solubility of the resulting PIs, as shown in Table 4 and Table S2. For example, the copolymer with 20 mol% *cardo*-AB-BCFL (**#15C**) became compatible with Route-C in contrast to the incompatibility of its methyl group-free counterpart (**#12T**). Furthermore, **#15C** provided a stable DMAc solution even at a high solid content (9.9 wt%) at room temperature. Thus, the dimethyl substituents of *cardo*-AB-BCFL greatly contributed to the improvement of solubility. An increase in the *cardo*-AB-BCFL content to 50 mol% (**#16C**) led to a highly soluble PI in a non-amide solvent (CPN) at a high solid content of 17 wt%. The resulting cast film exhibited a relatively high modulus (4.52 GPa) while maintaining the highest toughness (*ε*_b max_ = 14.4%) among the PIs listed in Table 4. This copolymer film (**#16C**) showed a somewhat decreased ∆*n*_th_, compared to that of the pristine system (**#0T**), with a concomitantly increased CTE. No clear trends of the deterioration of the film transparency and *T*_g_ were observed with an increase in the *cardo*-AB-BCFL content.

#### 3.5.4. *Cardo*-AP-BPFL-Modified Systems

A counterpart of ester-linked *cardo*-AB-BPFL, ether-linked *cardo*-AP-BPFL (Figure 19), was also used to modify CBDA/TFMB with the expectation of significant improvement in film toughness due to the flexible ether group of this modifier. The copolymer with 20 mol% *cardo*-AP-BPFL (**#18T**) was incompatible with Route-C owing to gelation during chemical imidization, as was the case for the corresponding copolymer using the ester-linked counterpart (**#12T**). Even increasing the *cardo*-AP-BPFL content to 50 mol% remained ineffective for improving the Route-C compatibility owing to precipitation during chemical imidization, in contrast to the corresponding copolymer using its ester-linked counterpart (**#13C**) with the Route-C compatibility. Thus, the solubility-improving effect of these modifiers was as follows: *cardo*-AP-BPFL (ether-linked) < *cardo*-AB-BPFL (ester-linked).

Despite a sufficiently high *η*_red_ value of the PAA (1.62 dL/g), the thermally imidized copolymer film with 20 mol% *cardo*-AP-BPFL (**#18T**) was unexpectedly too brittle to conduct mechanical property measurements. This PI film (**#18T**) exhibited higher *T*_d_^5^ values in both N_2_ and air atmospheres than the corresponding copolymer using the ester-linked counterpart (**#12T**), without contradicting the fact that the ether-connecting group is more thermally stable than the ester-connecting group [78].

#### 3.5.5. CTE–Δ*n*_th_ Correlation for the Systems Using *Cardo*-Type Modifiers

**Figure 23:**
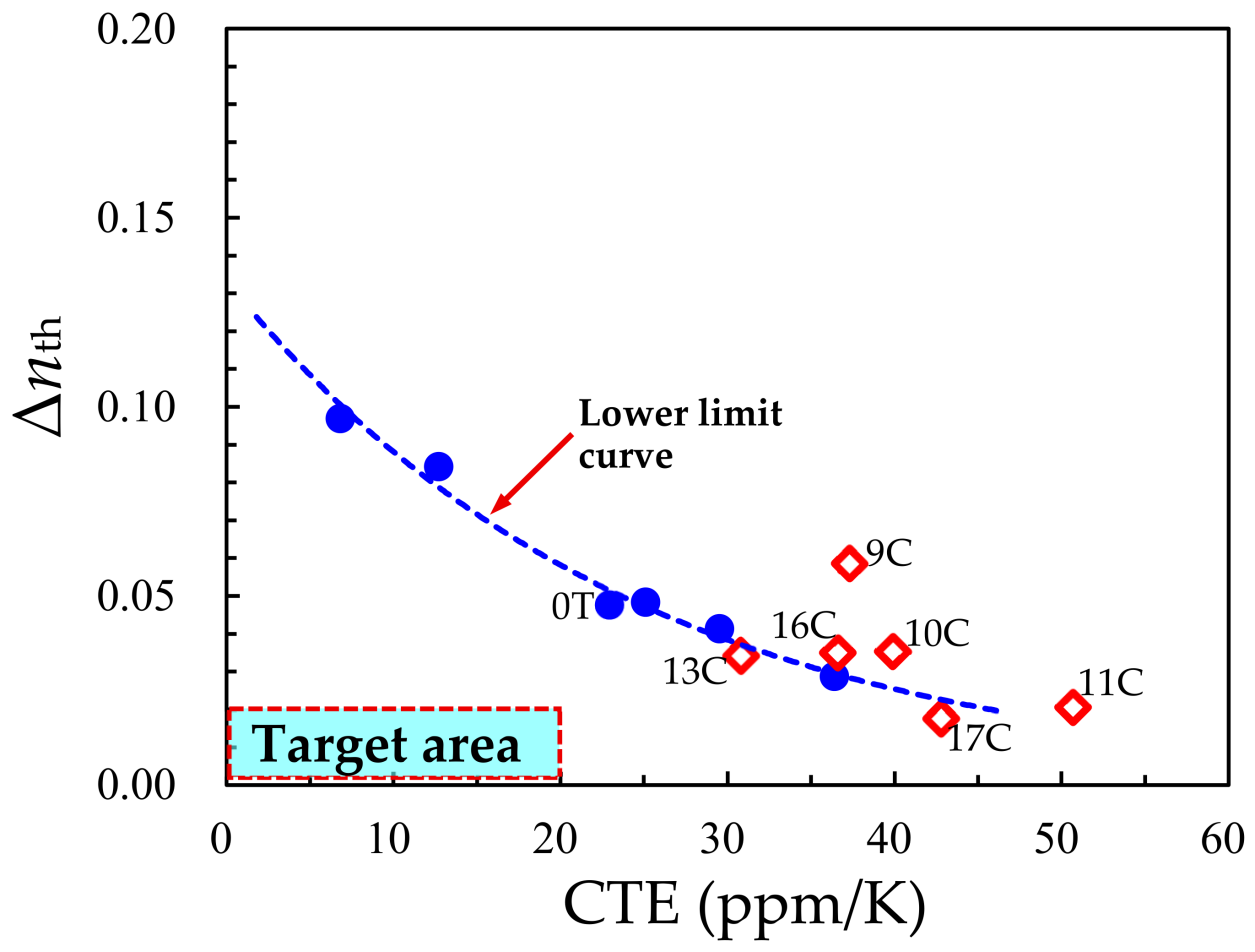
The CTE–Δ*n*_th_ relationship: (**◊**) PIs modified with *cardo*-type monomers and (**●**) CBDA/TFMB PI films prepared under different conditions (blue dashed fitted curve: the lower limit curve). The inserted numbers represent the sample code (Table 4).

Figure 23 shows the CTE–Δ*n*_th_ correlation diagram for the systems using various *cardo*-type modifiers listed in Table 4. Many of these plots (**◊**) were located above the lower limit curve, suggesting that they were ineffective in overcoming the trade-off between low CTE and low ∆*n*_th_. A few plots (**#13C** and **#17C**) only slightly exceeded the lower limit curve, although the observed effect was much smaller than expected. The *cardo*-AB-BPFL used in **#13C** was not advantageous for reducing CTE. This is probably due to a “hinge” structure common to *cardo*-type monomers, which reduces main-chain linearity due to chain distortion at the *sp*^3^ carbon atom, which consequently disturbs main-chain in-plane orientation during thermal imidization (Route-T) or solution casting (Route-C).

### 3.6. Effect of Spiro-Type Modifiers

The results discussed in Section 3.5 suggested that it is essential to exclude the hinge structures, such as those in the *cardo*-type modifiers, for achieving our goals. Therefore, next, we focused on *spiro*-type modifiers that have a unique structure comprising a planar xanthene (XAN) unit and a firmly/perpendicularly bound FL unit (Figure 2). Here, the long axis of the FL side group is expected to align in the *Z*-direction when the XAN-incorporated PI main chains are aligned in the *XY*-direction along with the “face-on” local orientation of the XAN molecular plane. The mutual perpendicular configuration between the XAN and FL molecular planes is supported by a distinct high-magnetic field shift in the ^1^H-NMR spectra (*δ* ≈ 6.2 ppm) of the 1,8-protons of the FL-pendant XAN (cf. *δ* = 7.25 ppm for the 1,8-protons of non-substituted XAN in DMSO-*d*_6_ at 400 MHz [57]), as mentioned in the Experimental Section. We have previously investigated the basic properties of PIs using *spiro*-TA-FLX and *spiro*-AB-FLX without considering the CTE–Δ*n*_th_ problem [38]. In this study, we re-examined the related systems to comprehensively discuss the effects of these *spiro*-type monomers by adding important optical properties.

#### 3.6.1. Modifications with Ether-Linked *Spiro*-Type Diamines

The polymerization results and film properties of CBDA/TFMB-based PIs modified using ether-linked *spiro*-type diamines (Figure 19) are summarized in Table 5. In these modified systems, PAAs with sufficiently high *η*_red_ values (≥ ~ 1.0 dL/g) were obtained, suggesting sufficiently high polyaddition reactivity of these modifier diamines with CBDA. The deterioration of the film transparency arising from copolymerization with these modifiers was limited. However, a prominent solubility-improving effect of these modifiers was not observed; consequently, Route-C remained incompatible, as exemplified by the copolymers with 20 mol% modifiers (**#19T**, **#22T**, and **#28T**). The features of systems using each modifier are described below.

**Table 5:** Polymerization results and film properties of the CBDA-based PIs modified using ether-linked *spiro*-type diamines and related systems. The photographs at the bottom of this table show the appearance of selected PI films (**#20C** and **#23T**).

No.	Diamine (mol%)	*η*_red_ (dL/g)	Cast ^c^	*T*_400_ (%)	YI	*λ*_0_ (nm)	*T*_tot_ (%)	Haze (%)	Δ*n*_th_	*ε*_opt_	*T*_g_ (°C)	CTE (ppm/K)	*E* (GPa)	*ε*_b_ (%) ave max	*σ*_b_ (GPa)	*T*_d_^5^ (N_2_) (°C)	*T*_d_^5^ (air) (°C)
0T	TFMB	1.63 ^a^	---	84.2	3.97	302	89.8	0.87	0.0477	2.68	345	22.9	6.57	5.2 8.0	0.19	444	432
19T	TFMB (80) *spiro*-AP-FLX (20)	1.26 ^a^	---	85.3	1.91	314	88.5	1.22	0.0297	2.79	--- ^d^	--- ^d^	--- ^d^	--- ^d^	--- ^d^	443	447
20T	TFMB (50) *spiro*-AP-FLX (50)	2.31 ^a^	---	75.5	7.55	317	87.6	0.66	0.0180	2.86	331	49.9	2.21	3.4 5.2	0.046	445	427
20C	TFMB (50) *spiro*-AP-FLX (50)	1.85 ^a^ 2.24 ^b^	DMAc 7.4 wt%	77.4	5.03	317	88.1	2.18	0.0360	2.87	356	39.1	3.95	8.0 11.3	0.14	449	433
21C	*spiro*-AP-FLX	2.50 ^a^ 1.64 ^b^	DMAc 7 wt%	80.0	3.41	317	87.7	1.71	0.0198	3.00	360	49.1	2.75	8.7 19.5	0.11	467	447
22T	TFMB (80) *spiro-m*AP-FLX (20)	1.45 ^a^	---	82.4	4.03	314	89.0	0.30	0.0249	2.77	326	43.5	--- ^d^	--- ^d^	--- ^d^	428	429
23T	TFMB (50) *spiro-m*AP-FLX (50)	0.99 ^a^	---	80.7	4.12	316	88.4	0.99	0.0091	2.88	302	47.3	--- ^d^	--- ^d^	--- ^d^	466	429
23C	TFMB (50) *spiro-m*AP-FLX (50)	0.99 ^a^ 1.14 ^b^	CPN 15 wt%	53.3	16.52	316	84.8	0.89	0.0317	2.88	304	39.3	4.11	4.8 6.9	0.13	449	432
24T	*spiro-m*AP-FLX	0.34 ^a^	---	Data not available because of the absence of film-forming ability													
25T	TFMB (80) *spiro-*TFAP-FLX (20)	2.36 ^a^	---	81.3	4.29	314	89.2	4.00	0.0238	2.71	--- ^e^	44.1	--- ^d^	--- ^d^	--- ^d^	439	425
25R ^f^	TFMB (80) *spiro-*TFAP-FLX (20)	2.41	DMAc 10.5 wt%	59.6	16.5	311	85.7	0.99	0.0354	2.73	369 ^g^	41.6	2.57	3.2 3.3	0.072	---	---
26T	TFMB (50) *spiro-*TFAP-FLX (50)	1.82 ^a^	---	78.6	4.91	315	88.3	2.18	0.0091	2.78	--- ^d^	--- ^d^	--- ^d^	--- ^d^	--- ^d^	442	431
26C	TFMB (50) *spiro-*TFAP-FLX (50)	1.82 ^a^ 2.28 ^b^	CPN 13 wt%	80.8	3.06	314	89.1	3.26	0.0453	2.79	339	42.2	3.82	9.7 21.1	0.13	456	437
27C	*spiro-*TFAP-FLX	1.08 ^a^ 1.16 ^b^	CPN 18 wt%	84.7	2.01	314	89.3	1.16	0.0198	2.84	328	56.3	2.98	9.4 13.1	0.12	460	449
28T	AB-TFMB (80) *spiro*-AP-FLX (20)	1.26 ^a^	---	79.1	4.09	342	84.6	0.72	0.0623	2.92	346	15.2	--- ^d^	--- ^d^	--- ^d^	448	433
29T ^h^	AB-TFMB (50) *spiro*-AP-FLX (50)	2.31 ^a^	---	79.9	3.75	338	87.8	0.75	0.0424	2.95	344	30.5	3.60	8.4 14.4	0.14	446	428

^a^ Data for PAAs. ^b^ Data for PIs prepared via Route-C. ^c^ Homogeneous PI solutions (at room temperature) used for the solution casting process. The solutions were prepared upon warming as appropriate.^d^ Data not available because of the difficulty of the measurements due to film brittleness. ^e^ Data not available because the specimen fractured during DMA. ^f^ Sample polymerized in NMP via Route-R. ^g^ *T*_g_ determined by TMA method. ^h^ Chemical imidization was successful (*η*_red_ = 1.85 dL/g (PAA) and 2.24 dL/g (PI)). However, it was difficult to obtain a sufficiently high solid content of the PI solution even in DMAc for the subsequent solution casting process. Thus, the PI film was prepared via Route-T. GPC data: $\bar{M}$_n_ = 1.02 × 10^5^ and $\bar{M}$_w_ = 1.84 × 10^5^ for **#26C**; $\bar{M}$_n_ = 9.15 × 10^4^ and $\bar{M}$_w_ = 1.84 × 10^5^ for **# 27C**.


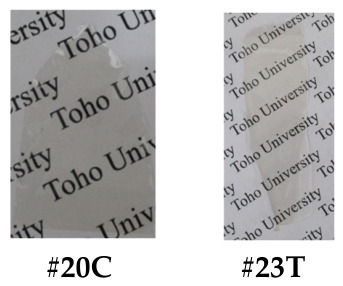

(a) *spiro*-AP-FLX-modified systems

Even though this diamine contains a flexible/rotatable ether linkage, copolymerization with this modifier did not result in a crucial decrease in the *T*_g_ compared to that of the pristine system (**#0T**, *T*_g_ = 345 °C), even at a high modifier content of 50 mol% (*T*_g_ = 331 °C for **#20T** and 356 °C for **#20C**). This PI film (**#20C**) also maintained relatively high transparency, as shown in the photograph at the bottom of Table 5. The observed high-*T*_g_ characteristics likely reflect that the internal rotation around the ether groups, which are usually easily rotatable, is significantly restricted, owing to a very large sweep volume of the FL-pendant XAN unit. However, from the comparison of **#0T**, **#20C**, and **#21C**, copolymerization using ether-linked *spiro*-AP-FLX inevitably resulted in a significant increase in the CTE with somewhat improved *ε*_b_ values.

We have previously demonstrated that an amide-linked CF_3_-containing diamine (AB-TFMB, bottom of Figure 18, Document S4, Scheme S6) is very effective for lowering CTE [32,37,39]. In the present study, when replacing TFMB in **#19T** with AB-TFMB (**#28T**), a quite low CTE of 15.2 ppm/K was obtained along with an undesirable significant increase in the ∆*n*_th_. Even at 50 mol% *spiro*-AP-FLX (**#29T**), a relatively low CTE (30.5 ppm/K) was maintained with the help of AB-TFMB. However, when CBDA/AB-TFMB was selected as another pristine system, as in **#29T**, the resulting copolymers tended to show somewhat lower transparency than when CBDA/TFMB as the pristine system was selected, as suggested by the comparison between **#19T** and **#28T**.

(b) *spiro-m*AP-FLX-modified systems

The observed increase in the CTE by copolymerization with *spiro*-AP-FLX was very likely attributable to a hindered in-plane orientation accompanied by the decreased main-chain linearity due to its ether linkage. Taking this interpretation into account, we attempted to modify the pristine system using an isomeric counterpart of *spiro*-AP-FLX, namely, *meta*-ether-linked *spiro-m*AP-FLX (Figure 19), with the expectation of lowering the CTE. This idea is inspired by the fact that PMDA/3,4′-ODA containing a *meta*-linkage has a somewhat higher modulus and lower CTE than PMDA/4,4′-ODA [79,80], which is probably related to an improved main-chain linearity of the former (Figure S8). The comparison of a copolymer modified with *spiro-m*AP-FLX (**#23C**) and its counterpart with *spiro-*AP-FLX (**#20C**) showed that the former has obviously improved solubility (Table S2). However, no prominent effect of using *spiro-m*AP-FLX on reducing CTE was observed. Thus, this attempt was unsuccessful.

There was an irregular case; the copolymer using *spiro-m*AP-FLX (**#23T**) led to a PI film with relatively high transparency, as shown in the photograph at the bottom of Table 5, whereas the same composition of PI via Route-C (**#23C**) formed a slightly colored film, although the reason for this is not clear. The latter may be related to the coloration of the CPN solution prepared for the subsequent solution casting; the CPN solutions of PIs become empirically colored upon heating at elevated temperatures in some cases.

(c) *spiro-*TFAP-FLX-modified systems

To significantly enhance the solubility-improving effect of the modifiers, CF_3_-substituted *spiro-*TFAP-FLX (Figure 19) was used. The film properties of the *spiro-*TFAP-FLX-modified systems are summarized in Table 5. When comparing the copolymer with 20 mol% *spiro-*TFAP-FLX (**#25R**) and its counterpart with 20 mol% CF_3_-free *spiro-*AP-FLX (**#19T**), the former became compatible with Route-C owing to its significantly improved solubility, whereas the latter was applicable to only Route-T owing to its insufficient solubility. The PI film (**#25R**) did not break during a convenient folding test at virtually zero curvature radius, suggesting the minimum-necessary ductility of this film. However, the *ε*_b_ value measured upon tensile testing suggested that this PI film is quite brittle. The CBDA/*spiro-*TFAP-FLX homo PI film (**#27C**) exhibited a lower *ε*_opt_ (2.84) than its CF_3_-free counterpart (CBDA/*spiro-*AP-FLX, **#21C**) (*ε*_opt_ = 3.00), reflecting the very low polarizability of the CF_3_ groups. From a comparison of the copolymer with 50 mol% *spiro-*TFAP-FLX (**#26C**) and the corresponding copolymer with 50 mol% CF_3_-free *spiro-*AP-FLX (**#20C**), the former exhibited an appreciable toughening effect.

As the *spiro-*TFAP-FLX content increases from **#25R** to **#26C** and **#27C**, a gradual increase in the CTE was observed with an unexpectedly small slope despite the presence of the ether linkage in this modifier (Figure 20A(b)).

(d) CTE–Δ*n*_th_ correlation for the systems modified using ether-linked *spiro*-type diamines

**Figure 24:**
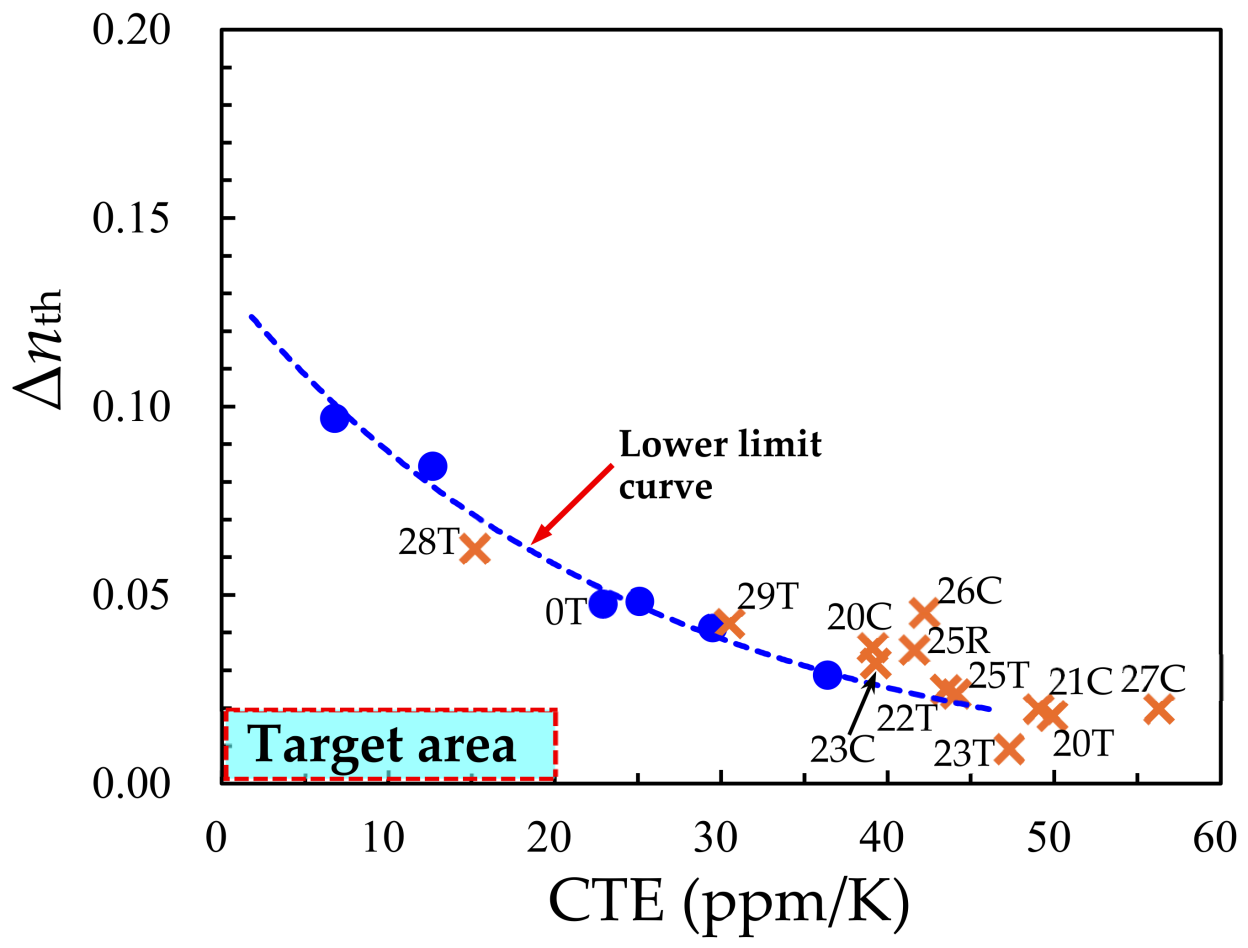
The CTE–Δ*n*_th_ relationship: (**×**) PIs modified with ether-linked *spiro*-type monomers and (**●**) CBDA/TFMB PI films prepared under different conditions (blue dashed fitted curve: the lower limit curve). The inserted numbers represent the sample code (Table 5).

Figure 24 exhibits the CTE–Δ*n*_th_ correlation diagram for the systems modified using ether-linked *spiro*-type diamines listed in Table 5. Many of these plots (×) were positioned above the lower limit curve, indicating that these modifications were ineffective. On the other hand, **#28T** and **#23T** slightly exceeded the lower limit curve, although the effect was not prominent. Although the former (**#28T**), which is the combination of AB-TFMB and CF_3_-containing *spiro-*TFAP-FLX, was satisfactory in terms of the desired low-CTE property, its observed Δ*n*_th_ was too large. Conversely, the latter (**#23T**), modified using *spiro-m*AP-FLX, was satisfactory in terms of low Δ*n*_th_, but its observed CTE was not.

#### 3.6.2. Modification with Ester-Linked *Spiro*-Type Monomers

To reduce CTE without the help of AB-TFMB, the connecting group in the above-mentioned ether-linked modifiers was replaced with a more rigid ester group. The polymerization results and film properties of the CBDA/TFMB-based systems modified using ester-linked *spiro*-type monomers (Figure 19) are summarized in Table 6. These monomers had sufficiently high polyaddition reactivity, as suggested by the sufficiently high *η*_red_ values of the resulting PAAs. Additionally, these modifiers significantly improved their solubility (Table S2). The features of the systems modified using each modifier are described below.

**Table 6:** Polymerization results and film properties of the CBDA-based PIs modified using ester-linked *spiro*-type monomers and related systems. The photographs at the bottom of this table show the appearance of selected PI films (**#31C** and **#38C**).

No.	TCDA (mol%)	Diamine (mol%)	*η*_red_ (dL/g)	Cast ^c^	*T*_400_ (%)	YI	*λ*_0_ (nm)	*T*_tot_ (%)	Haze (%)	Δ*n*_th_	*ε*_opt_	*T*_g_ (°C)	CTE (ppm/K)	*E* (GPa)	*ε*_b_ (%) ave max	*σ*_b_ (GPa)	*T*_d_^5^ (N_2_) (°C)	*T*_d_^5^ (air) (°C)
0T	CBDA	TFMB	1.63 ^a^	---	84.2	3.97	302	89.8	0.87	0.0477	2.68	345	22.9	6.57	5.2 8.0	0.19	444	432
30C	CBDA (70) *spiro*-TA-FLX (30)	TFMB	0.99 ^a^ 1.20 ^b^	DMAc 7 wt%	69.2	3.86	348	88.9	1.50	0.0400	2.84	--- ^e^	27.9	4.62	5.9 7.2	0.15	454	447
31C	CBDA (50) *spiro*-TA-FLX (50)	TFMB	1.22 ^a^ 1.44 ^b^	DMAc 7 wt%	66.7	3.31	353	88.8	1.13	0.0506	2.86	345	29.0	5.10	6.7 9.9	0.18	452	445
32C	*spiro*-TA-FLX	TFMB	1.53 ^a^ 1.17 ^b^	GBL 9.1 wt%	48.3	3.74	367	88.2	1.90	0.0367	2.89	323	45.6	2.77	6.1 9.8	0.09	491	471
33T	CBDA	AB-TFMB	8.21 ^a^	---	70.0	7.60	345	85.6	2.86	0.0810	2.67	342	11.7	5.76	6.2 9.5	0.16	445	438
34T	CBDA (70) *spiro*-TA-FLX (30)	AB-TFMB	3.55 ^a^	---	53.2	6.26	360	87.7	0.81	0.0530	2.92	--- ^d^	26.8	--- ^d^	--- ^d^	--- ^d^	442	437
34C	CBDA (70) *spiro*-TA-FLX (30)	AB-TFMB	3.55 ^a^ 4.46 ^b^	DMAc 8 wt%	71.7	2.61	345	87.5	1.14	0.1160	2.97	354	5.6	--- ^d^	--- ^d^	--- ^d^	453	441
35T	CBDA (50) *spiro*-TA-FLX (50)	AB-TFMB	2.35 ^a^	---	37.1	8.64	370	87.4	0.76	0.0450	2.97	--- ^d^	36.5	--- ^d^	--- ^d^	--- ^d^	443	434
35C	CBDA (50) *spiro*-TA-FLX (50)	AB-TFMB	2.35 ^a^ 2.11 ^b^	DMAc 8 wt%	59.3	3.54	357	87.7	1.17	0.0847	2.98	350	14.6	--- ^d^	--- ^d^	--- ^d^	448	449
36C	*spiro*-TA-FLX	AB-TFMB	1.46 ^a^ 1.37 ^b^	CPN 8 wt%	43.4	6.70	368	87.0	1.14	0.0643	3.03	315	22.2	2.57	9.6 13.7	0.11	451	455
37T	CBDA	TFMB (80) *spiro*-AB-FLX (20)	1.71 ^a^	---	76.2	5.87	322	87.7	1.26	0.0256	2.76	--- ^d^	--- ^d^	--- ^d^	--- ^d^	--- ^d^	441	431
38C	CBDA	TFMB (50) *spiro*-AB-FLX (50)	1.07 ^a^ 1.63 ^b^	CPN 8 wt%	82.8	3.67	320	87.0	2.29	0.0378	2.83	364	26.0	4.63	7.2 12.2	0.17	448	434
39C	CBDA	*spiro*-AB-FLX	1.53 ^a^ 1.75 ^b^	CPN 8 wt%	79.2	5.66	328	87.3	1.20	0.0300	3.01	371	39.0	3.01	5.6 8.4	0.11	449	466
40T	CBDA	AB-TFMB (80) *spiro*-AB-FLX (20)	1.27 ^a^	---	78.0	4.50	343	88.1	0.45	0.0456	2.90	423	24.6	--- ^d^	--- ^d^	--- ^d^	445	429
41C	CBDA	AB-TFMB (50) *spiro*-AB-FLX (50)	1.88 ^a^ 1.95 ^b^	DMAc 10 wt%	80.9	4.31	340	87.7	1.43	0.0638	2.93	360	21.9	4.72	7.3 10.9	0.18	450	443
42C	CBDA	*t*-CHDA (50) *spiro*-AB-FLX (50)	1.15 ^a^ 0.93 ^b^	DMAc 10 wt%	80.5	5.46	325	88.3	1.48	0.0182	2.91	335	40.8	1.98	5.6 9.0	0.067	414	430
43C	CBDA	TFMB (50) *a*-*spiro*-AB-FLX (50)	2.38 ^a^ 6.92 ^b^	DMAc 10 wt%	80.8	3.36	320	87.7	2.81	0.0733	2.90	351	10.5	6.12	3.7 6.0	0.15	440	427

^a^ Data for PAAs. ^b^ Data for PIs prepared via Route-C. ^c^ Homogeneous PI solutions (at room temperature) used for the solution casting process. The solutions were prepared upon warming as appropriate. ^d^ Data not available because of the difficulty of the measurements due to film brittleness. ^e^ Data not available because the specimen fractured during DMA. GPC data: $\bar{M}$_n_ = 5.09 × 10^4^ and $\bar{M}$_w_ = 1.12 × 10^5^ for **# 32C;** $\bar{M}$_n_ = 2.26 × 10^4^ and $\bar{M}$_w_ = 6.35 × 10^4^ for **#36C**.


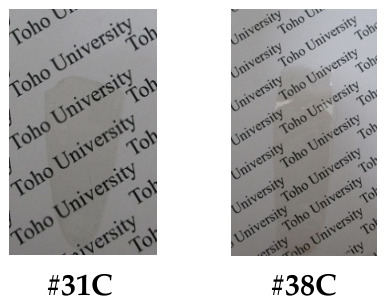

(a) Modification with *spiro*-TA-FLX

As shown in Table 6, the modifications of CBDA/TFMB or CBDA/AB-TFMB using *spiro*-TA-FLX (Figure 19) resulted in gradual decreases in T_400_, owing to the afore-mentioned gradual increase in the weak intramolecular (through-bond) CT interactions [10,11,12] between the trimellitimide unit (from *spiro*-TA-FLX) and *N*-aromatic units (from TFMB or AB-TFMB) (Figure S7b). The observed increase in the CTE with increasing *spiro*-TA-FLX content from **#0T** to **#30C** and **#31C** was unexpectedly gradual. No clear toughening effect was observed during the increase in the *spiro*-TA-FLX content. These modified PI films (**#30C** and #31C) showed relatively high transparency with controlled YI values, as exemplified by the appearance of **#31C** at the bottom of Table 6.

Significantly reduced CTEs were observed in the CBDA/AB-TFMB-based PIs modified using *spiro*-TA-FLX (CTE = 5.6 ppm/K for **#34C** and 14.6 ppm/K for **#35C**), but with the concomitant significant increases in Δ*n*_th_.

(b) Modification with *spiro*-AB-FLX

As shown in Table 6, the CBDA/TFMB-based copolymer (**#38C**) with 50 mol% *spiro*-AB-FLX (Figure 19) exhibited obviously high transparency (*T*_400_), as is evident from the appearance of this film at the bottom of this table, and a low CTE (26.0 ppm/K), but without a clear toughening effect.

Even though *t*-CHDA is usually disadvantageous for ensuring the solubility of the resulting PIs, the CBDA/*t*-CHDA(50);*spiro*-AB-FLX(50) copolymer (**#42C**) was unexpectedly compatible with Route-C, thus suggesting a significant solubility-improving effect of this modifier.

(c) Modification with *asymmetric spiro*-AB-FLX

**Scheme 29:**
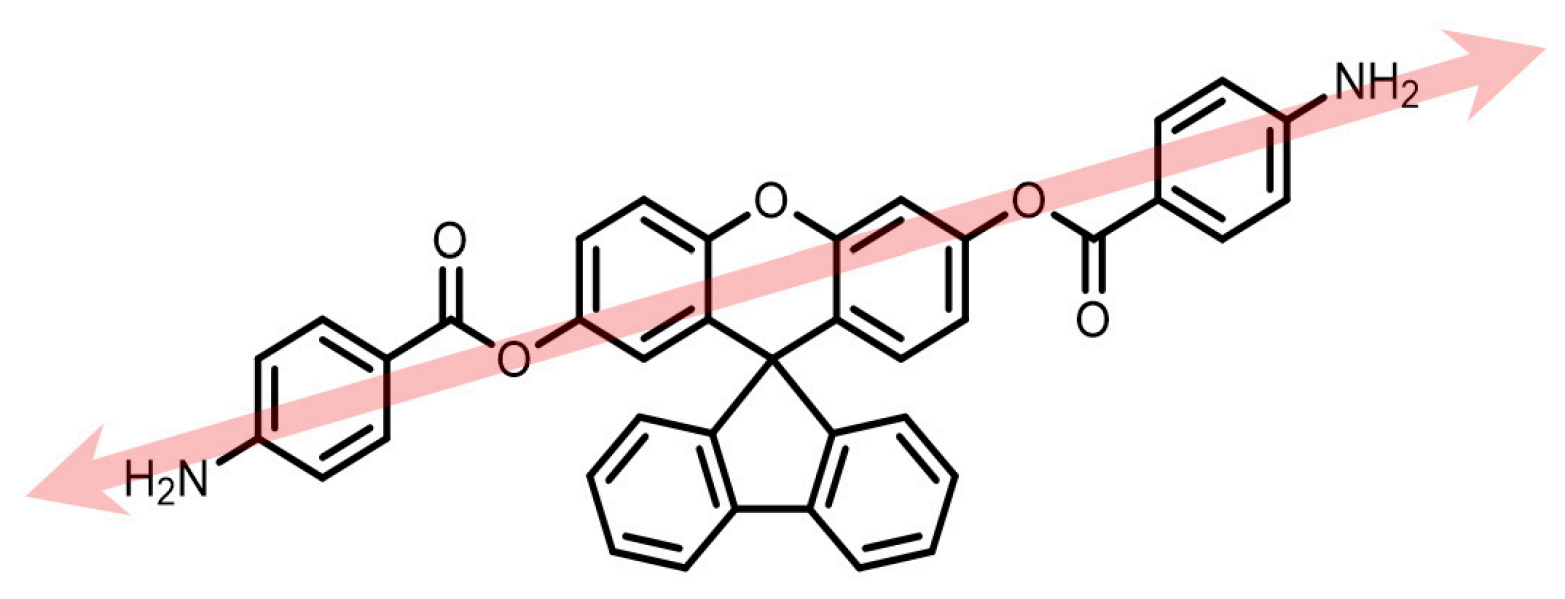
Molecular structure of *a-spiro*-AB-FLX. The double-headed arrow indicates a highly linear structure across the two functional groups.

We have previously investigated the basic properties of PIs derived from an asymmetric structure of ester-linked *spiro*-type monomers [38]. Among them, the properties of the CBDA/TFMB-based system modified using 50 mol% asymmetric (*a*)-*spiro*-AB-FLX (**#43C**) were compared with those of its symmetric counterpart (**#38C**) in Table 6. The former exhibited a prominent feature, namely, a significantly reduced CTE (10.5 ppm/K) compared to that of the latter (26.0 ppm/K). This is closely related to a mutual linear configuration between the two functional groups of *a*-*spiro*-AB-FLX (Scheme 29), which does not deteriorate the original main-chain linearity of pristine CBDA/TFMB when this modifier was copolymerized.

The observed very low CTE of the copolymer film (**#43C**) arises from self-in-plane orientation during solution casting [26,32,37,38,39,47,48,65], but with a concomitant significantly increased Δ*n*_th_.

(d) CTE–Δ*n*_th_ correlation for the systems modified using ester-linked *spiro*-type monomers

**Figure 25:**
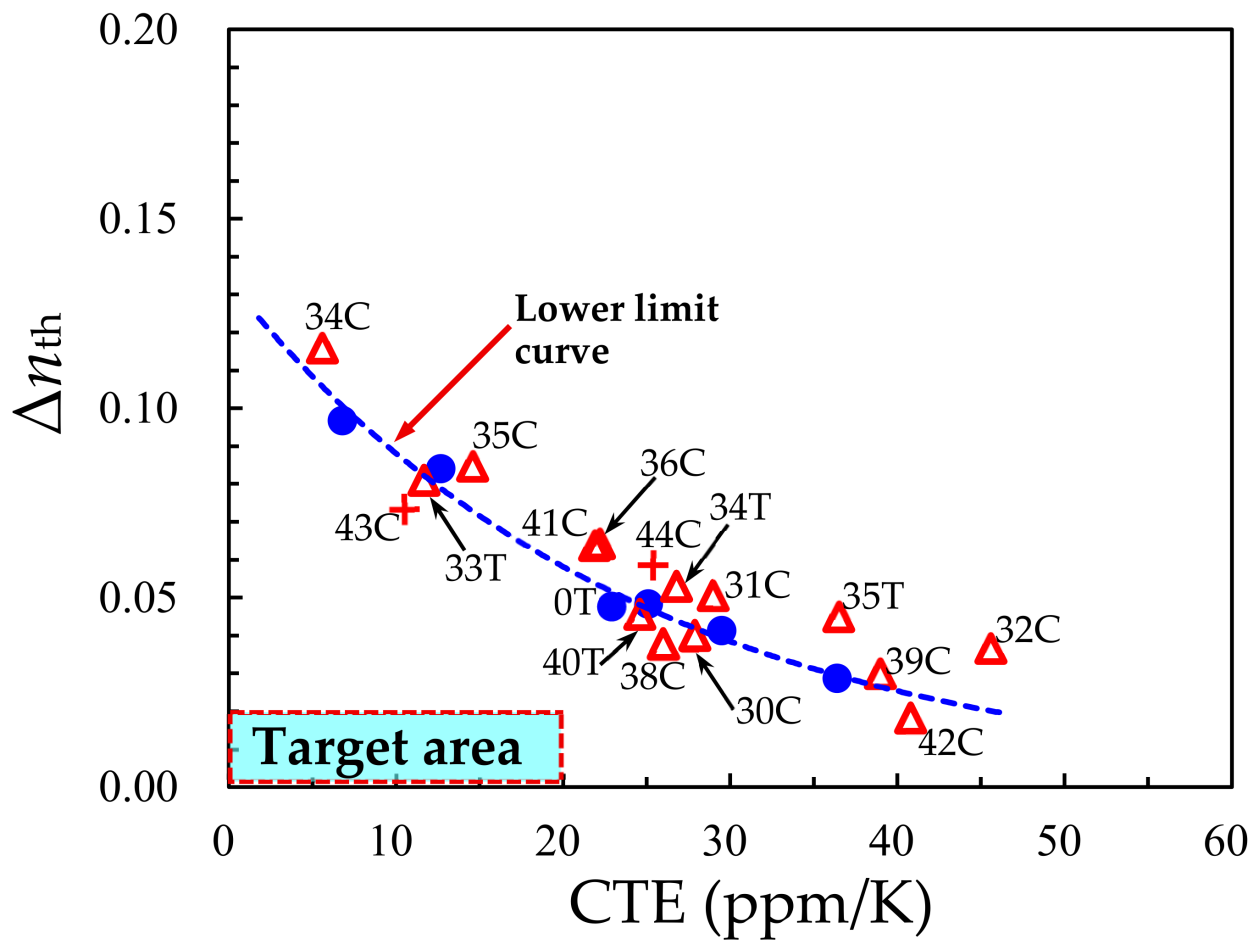
The CTE–Δ*n*_th_ relationship: (**∆**) PIs modified with ester-linked *spiro*-type monomers and (**+**) ester-linked asymmetric *spiro*-type monomers [CBDA/TFMB(50);*a*-*spiro*-AB-FLX(50) (**#43C**), *a*-*spiro*-TA-FLX/TFMB (**#44C**)] and (**●**) CBDA/TFMB PI films prepared under different conditions (blue dashed fitted curve: the lower limit curve). The inserted numbers represent the sample code (Table 6).

Figure 25 exhibits the CTE–Δ*n*_th_ correlation diagram for the systems modified using ester-linked *spiro*-type monomers listed in Table 6. Many of these plots (**∆**) were located above the lower limit curve, suggesting the ineffectiveness of the ester-linked *spiro*-type monomers. On the other hand, the CBDA/TFMB-based copolymer (**#38C**) modified using 50 mol% *spiro*-AB-FLX slightly exceeded the lower limit curve.

Its counterpart using 50 mol% *a*-*spiro*-AB-FLX (**#43C**) also provided a plot (+) located slightly below the lower limit curve.

### 3.7. Comparison of the Modification Effect of Cardo-Type, Ether-Linked Spiro-Type, and Ester-Linked Spiro-Type Monomers and the Impact of Polymerization/Film Preparation Route

As typical examples, the superiority of each modifier for the CBDA/TFMB(50);modifier(50) copolymers was compared, as summarized in Table 7. The system using ester-linked *spiro*-AB-FLX (**#38C**) demonstrated better overall properties than its counterparts using the ester-linked *cardo*-type modifier (**#13C**), ether-linked *spiro*-type modifier (**#20C**), and ester-linked *a*-*spiro*-type modifier (**#43C**), with only one exception (**#38C** was inferior to **#43C** in terms of low CTE).

**Figure 26:**
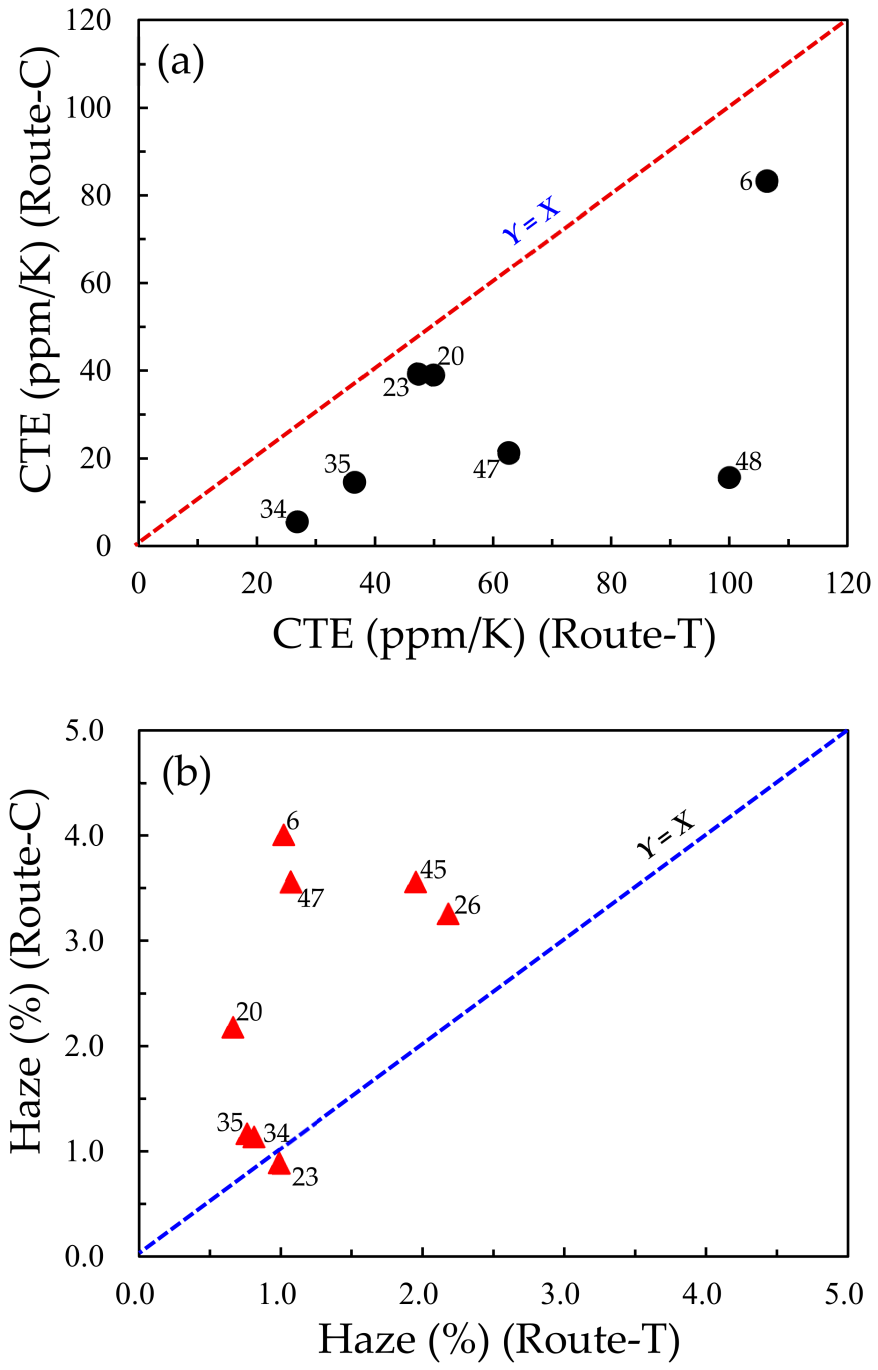
Impacts of the polymerization/film preparation route (C and T) on CTE (**a**) and haze (**b**) of the resulting PI films in a comparison between the identical chemical compositions of PIs. The inserted numbers represent the sample code.

When comparing Route-C with Route-T, a major benefit of Route-C becomes evident in Figure 26a. In a plot of the CTE data of the PI films prepared via Route-C (ordinate) against those of the identical chemical compositions of the PI films prepared via Route-T (abscissa), all plots are positioned below the *Y* = *X* line. This confirms that Route-C is always more effective in reducing CTE than Route-T.

Another benefit of Route-C is also evident when the optical transparency of the films prepared via Route-C is compared with that of the films via Route-T, especially in **#26**, **#34**, and **#35**. This trend is always true as long as undesirable solvents such as NMP for solution casting are not used, as discussed later.

However, Route-C also had a disadvantage; PI films prepared by solution casting from PI solutions via Route-C often showed higher haze than those prepared via Route-T, as illustrated in Figure 26b (haze (Route-C, ordinate) vs. haze (Route-T, abscissa)). This is obvious from the fact that many of these plots are located above the *Y* = *X* line. This strong tendency is probably related to the difference in the affinity of the PI–solvent and PAA–solvent systems (if the influence of crystallization on haze is not taken into account). Assuming that haze mainly originates from a sort of “partial precipitation” or “heterogeneous aggregation” during solvent evaporation of coatings, haze is probably closely associated simply with the polymer solubility in the solvents used. If so, such partial precipitation can easily occur in “nearly saturated” PI solutions when the solvent runs out due to evaporation, whereas there is little chance of such partial precipitation during solution casting of PAAs (Route-T) because the originally excellent solvation of PAAs in amide solvents is always retained during solution casting, regardless of the PAA structures, even when the solvent runs out due to evaporation.

### 3.8. Effects of Xanthene-Pendant Diaminofluorenes

**Figure 27:**
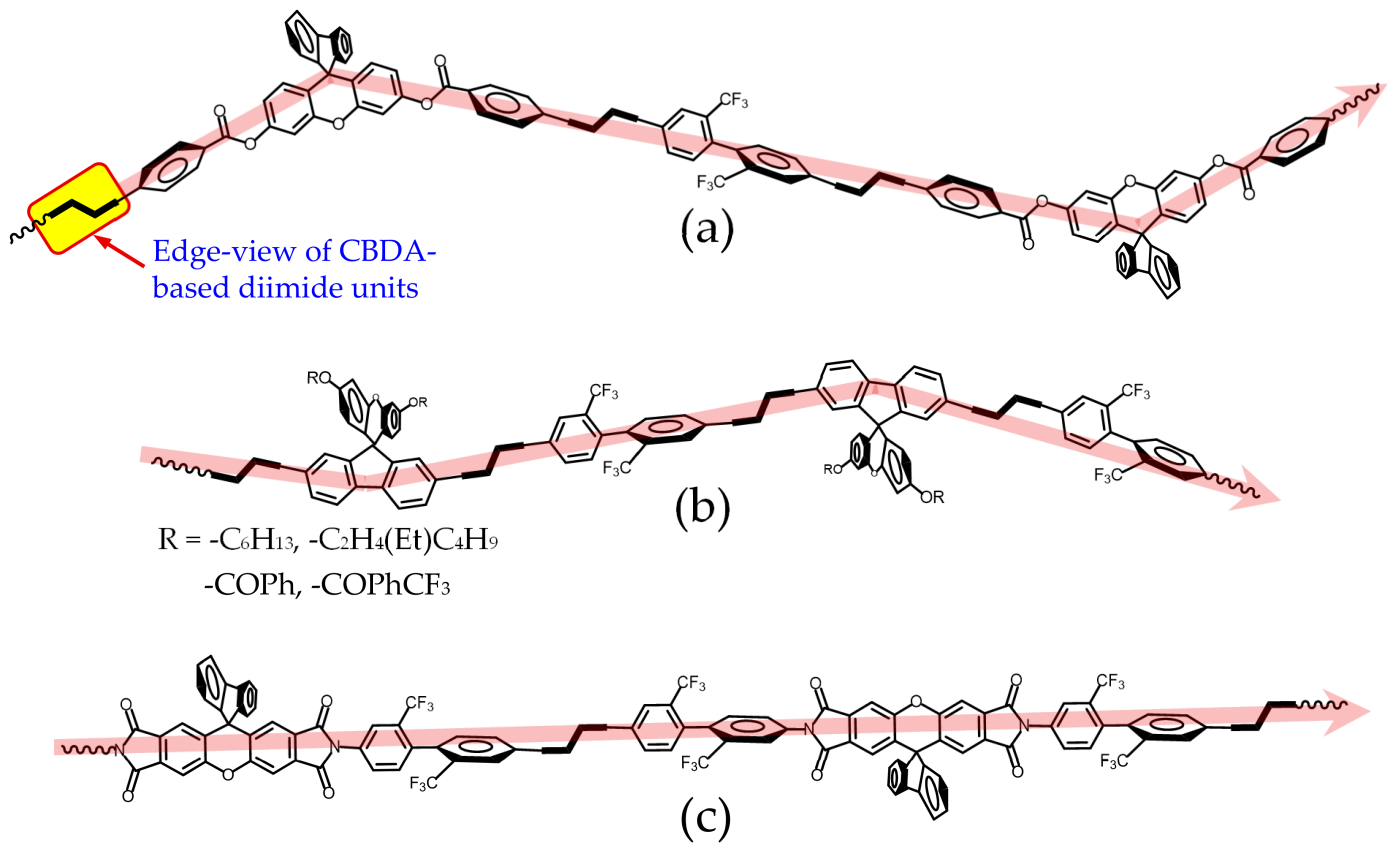
Schematically drawn chain linearity (red arrows) of the extended forms for the CBDA/TFMB-based PIs using different types of modifiers: (**a**) Ester-linked *spiro*-type diamines, (**b**) XAN-pendant diaminofluorenes, and (**c**) *spiro*-2367XADA.

So far, we have demonstrated that, when extended chain forms were schematically depicted, PI systems with higher linearity (lower extents of meandering of main chains) have a strong trend that the corresponding actual PI films show lower CTE values [26,32,39,41,47,65,68,73]. The extended chain forms of CBDA/TFMB-based PIs modified using typical modifiers are schematically drawn in Figure 27. The system modified using ester-linked *spiro*-AB-FLX (**#38C**), which comprises relatively linear main chains (Figure 27a), indeed exhibited a low CTE (26.0 ppm/K), thus suggesting that the above-mentioned criterion is reasonable. However, its depicted extended form is slightly meandering owing to a slightly distorted configuration at the COO–XAN–OCO unit (Figure 27a). Therefore, as long as this type of modifier is applied, there seems to be a limit to the reduction of CTE. In contrast, the use of its asymmetric counterpart (ester-linked *a*-*spiro*-AB-FLX, Scheme 29) enabled us to obtain an extremely low CTE (10.5 ppm/K for **#43C**). However, the synthesis of this asymmetric modifier was complex; it required a complicated purification process of the intermediate bisphenol (asymmetric FL-DHX) [38].

To overcome the low-CTE–low-∆*n*_th_ trade-off without the help of the asymmetric modifiers, we focused on new modifiers, XAN-pendant 2,7-diaminofluorenes (Figure 19), which can maintain a higher main-chain linearity (Figure 27b) that is advantageous for further reducing CTE. The features of the systems modified using a series of XAN-pendant 2,7-diaminofluorenes are described below.

#### 3.8.1. Modifications with *Spiro*-C_n_X-DAFL

*spiro*-C_n_X-DAFL, which contains long alkyl groups connected to the XAN unit, was applied as a new modifier with the expectation of *Z*-direction alignment of the long axis of XAN together with the alkyl chains. The polymerization results and film properties of the systems modified using *spiro*-C_n_X-DAFL with *n*-hexyl or 2-ethylhexyl group are summarized in Table 8. *spiro*-C_n_X-DAFL showed high polyaddition reactivity with CBDA and resulted in PAAs with sufficiently high *η*_red_ values. The observed high reactivity of these modifiers likely indicates that their bulky side groups (alkoxyl-pendant XAN unit) did not participate in steric hindrance during the polyaddition with CBDA, probably owing to the spatial separation of the side groups from the functional groups as a result of the XAN–FL perpendicular arrangement.

**Table 8:** Polymerization results and film properties of the CBDA-based PIs modified using xanthene-pendant 2,7-diaminofluorenes and related systems. The photographs at the bottom of this table show the appearance of selected PI films (**#47C**, **#48T**, and **#48C**).

No.	Diamine (mol%)	*η*_red_ (dL/g)	Cast	*T*_400_ (%)	YI	*λ*_0_ (nm)	*T*_tot_ (%)	Haze (%)	Δ*n*_th_	*ε*_opt_	*T*_g_ (°C)	CTE (ppm/K)	*E* (GPa)	*ε*_b_ (%) ave max	*σ*_b_ (GPa)	*T*_d_^5^ (N_2_) (°C)	*T*_d_^5^ (air) (°C)
0T	TFMB	1.63 ^a^	---	84.2	4.0	302	89.8	0.87	0.0477	2.68	345	22.9	6.57	5.2 8.0	0.19	444	432
44T	TFMB (80) *spiro*-C_6_X-DAFL (20)	0.84 ^a^	---	67.8	12.1	335	87.8	0.99	0.0295	2.72	329	40.3	3.78	1.5 2.8	0.051	403	385
45T	TFMB (30) *spiro*-C_6_X-DAFL (70)	0.37 ^a^	---	15.5	63.8	344	78.7	1.95	0.0135	3.00	298	93.4	--- ^d^	--- ^d^	--- ^d^	416	356
45C	TFMB (30) *spiro*-C_6_X-DAFL (70)	0.37 ^a^ 0.75 ^b^	NMP 15 wt%	48.7	25.1	343	85.3	3.56	0.0225	2.85	--- ^d^	--- ^d^	--- ^d^	--- ^d^	--- ^d^	417	346
46C	*spiro*-C_6_X-DAFL	0.65 ^a^ 1.21 ^b^	DMAc 15 wt%	60.6	9.5	346	86.4	7.79	0.0189	3.16	280	56.8	--- ^d^	--- ^d^	--- ^d^	---	---
47T	TFMB (50) *spiro*-C_8_X-DAFL (50)	1.60 ^a^	---	58.6	14.8	344	88.4	1.07	0.0138	2.78	344 ^c^	62.6	2.20	2.8 4.2	0.050	396	342
47C	TFMB (50) *spiro*-C_8_X-DAFL (50)	1.60 ^a^ 8.16 ^b^	NMP 9.1 wt%	45.8	24.4	341	81.2	3.56	0.0388	2.86	389 ^c^	21.3	---	---	---	408	347
48T	TFMB (30) *spiro*-C_8_X-DAFL (70)	1.32 ^a^	---	67.3	11.3	345	88.9	0.43	0.0051	2.82	310	99.9	1.92	3.9 7.5	0.044	390	338
48C	TFMB (30) *spiro*-C_8_X-DAFL (70)	1.32 ^a^ 6.85 ^b^	NMP 8.0 wt%	48.0	16.3	340	84.4	23.6	---	---	343	15.7	---	---	---	405	338
49T	*spiro*-C_8_X-DAFL	3.72 ^a^	---	59.9	15.7	346	87.9	2.66	0.0066	2.87	299	121.8	1.45	6.3 8.9	0.044	388	329

^a^ Data for PAAs. ^b^ Data for PIs prepared via Route-C. ^c^ *T*_g_ determined by the TMA method. ^d^ Data not available because of the difficulty of the measurements due to film brittleness.


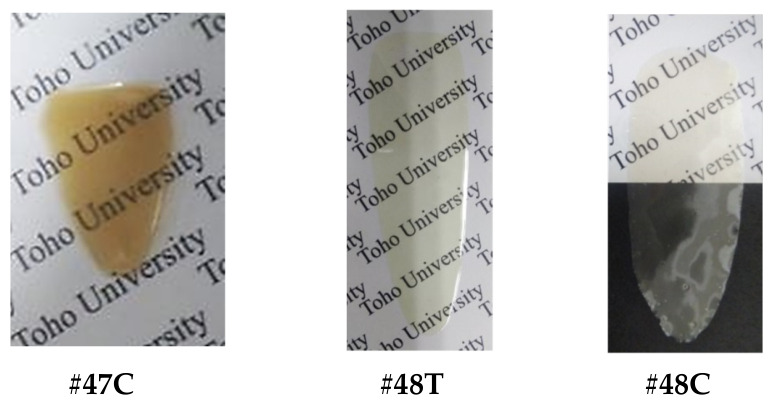

(a) *spiro*-C_6_X-DAFL

The CBDA/TFMB-based copolymer systems modified with 20 and 50 mol% *spiro*-C_6_X-DAFL were not compatible with Route-C owing to gelation during chemical imidization. At a *spiro*-C_6_X-DAFL content of 20 mol%, the PI film prepared via Route-T (**#44T**) showed significantly decreased film transparency compared to the pristine system (CBDA/TFMB, **#0T**). This is probably related to the presence of the less thermally stable *n*-hexyl group in this modifier, which can participate in the formation of a trace amount of unknown colored product via its partial thermal decomposition during thermal imidization, corresponding to its decreased *T*_d_^5^(N_2_) (403 °C). The increase in the *spiro*-C_6_X-DAFL content to 70 mol% resulted in a greater decrease in film transparency, probably owing to the increased *n*-hexyl group content.

With increasing *spiro*-C_6_X-DAFL content from **#0T** to **#44T** and **#45T**, a significant decrease in *T*_g_ was observed with an abrupt increase in the CTE (Figure 20A(c)), possibly reflecting a significant disruption of dense main-chain stacking by its bulky and flexible *n*-hexyl groups.

The CBDA/TFMB-based copolymer modified with 70 mol% *spiro*-C_6_X-DAFL (**#45C**) became compatible with Route-C. However, the solvent for dissolving the resulting PI powder form at a high solid content for subsequent solution casting was limited to NMP (Table S3). Even though Route-C was applicable, the resultant NMP-cast PI film (**#45C**) was significantly colorized, as suggested by its high YI value (25.1), which was lower than that of the corresponding thermally imidized film (YI = 63.8 for **#45T**). The unexpected significant coloration of the former is probably affected by the use of NMP as the casting solvent, which is prone to cause an appreciable coloration of PI films, particularly when the films are heated at elevated temperatures. Thus, ensuring sufficient PI solubility in solvents other than NMP (e.g., DMAc) is undoubtedly one of the keys to achieving this goal. Indeed, the CBDA/*spiro*-C_6_X-DAFL homo PI (**#46C**), which showed significantly improved solubility (Table S3), allowed the solution casting from its DMAc solution, and consequently, this film had dramatically restored transparency (YI = 9.5).

(b) *spiro*-C_8_X-DAFL

To enhance the less noticeable solubility-improving effect of *spiro*-C_6_X-DAFL, its analogue, *spiro*-C_8_X-DAFL, where the *n*-hexyl group of the former was replaced with the branched 2-ethylhexyl group, was used to modify the pristine system (CBDA/TFMB). The copolymer with 50 mol% *spiro*-C_8_X-DAFL (**#47T**) resulted in a somewhat colored PI film (YI = 14.8) with a significantly decreased *T*_d_^5^(N_2_) (396 °C), which is ascribed to the less thermally stable 2-ethylhexyl group, as discussed for the *spiro*-C_6_X-DAFL-modified systems. The same chemical composition of the NMP-cast PI film (**#47C**) unexpectedly caused a further deterioration in transparency (YI = 24.4), as is evident from the appearance of this film at the bottom of Table 8. This is probably due to the use of easy-to-color NMP as the solvent for solution casting.

The *spiro*-C_8_X-DAFL-modified PI film (**#48T**) showed significantly restored transparency (YI = 11.3 and haze = 0.43%), as shown in the photograph at the bottom of Table 8, compared with the corresponding *spiro*-C_6_X-DAFL-modified PI film (**#45T**) with a very high YI (63.8).

The applicable solvents for solution casting in the *spiro*-C_8_X-DAFL-modified system (**#48C**) were still limited to NMP, as in the *spiro*-C_6_X-DAFL-modified counterpart (**#45C**). Thus, the replacement of the *n*-hexyl group with the branched 2-ethylhexyl group was less effective in significantly improving the solubility of the resulting PIs. The film of the former (**#48C**) was also significantly turbid (haze = 23.6%, see its photograph at the bottom of Table 8) in contrast to the very low haze (0.43%) of its counterpart with the same chemical composition (**#48T**). This probably results from “partial precipitation” during solution casting from its “nearly saturated” PI solution. This highly turbid film (**#48C**) made it difficult to accurately measure its ∆*n*_th_. Surprisingly, this PI film (**#48C**) resulted in a dramatically reduced CTE (15.7 ppm/K, Table 8), probably owing to significant self-in-plane orientation generated during solution casting. A similar prominent CTE-reducing effect of Route-C (Figure 26a) was also observed in **#47C** (CTE = 21.3 ppm/K, Table 8).

As in the *spiro*-C_6_X-DAFL-modified system, an increase in the *spiro*-C_8_X-DAFL content from **#0T** to **#47T**, **#48T**, and **#49T** gave rise to a monotonous reduction in *T*_g_ with an abrupt increase in the CTE (Figure 20A(d)).

(c) CTE–Δ*n*_th_ correlation for the systems modified using *spiro*-C_n_X-DAFL

**Figure 28:**
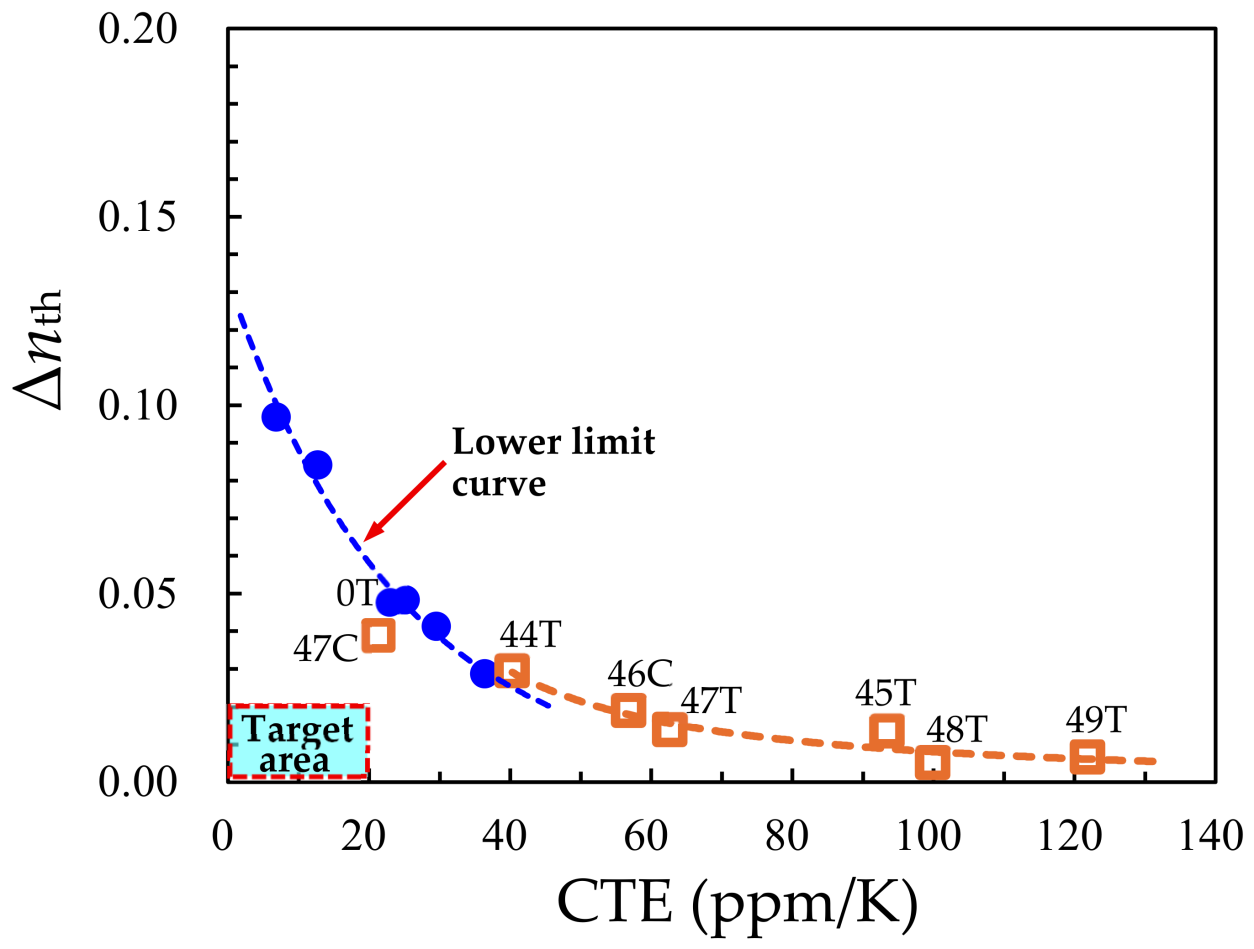
The CTE–Δ*n*_th_ relationship: (**□**) PIs modified with *spiro*-C_n_X-DAFL (red dashed fitted curve) and (**●**) CBDA/TFMB PI films prepared under different conditions (blue dashed fitted curve: the lower limit curve). The inserted numbers represent the sample code (Table 8).

Figure 28 exhibits the CTE–Δ*n*_th_ correlation diagram for the systems modified using *spiro*-C_n_X-DAFL listed in Table 8. Many of these plots (**□**) were located above the lower limit curve, thus suggesting that these modifiers were ineffective for the present purpose, although a certain positive effect was observed in the CBDA/TFMB(50);*spiro*-C_8_X-DAFL(50) system (**#47C**). Unfortunately, this system also had poor solubility-improving effects and a crucial film coloration problem triggered by the use of easy-to-color NMP.

#### 3.8.2. Modification with *Spiro*-TFBzX-DAFL

The above results of the *spiro*-C_n_X-DAFL-modified systems suggested the importance of the thermal stability of the XAN-pendant diaminofluorene modifiers and their solubility-enhancing effect to ensure the excellent transparency of the resulting PI films. Therefore, we used *spiro*-TFBzX-DAFL as a new modifier, wherein the thermally unstable alkoxy group in *spiro*-C_n_X-DAFL was replaced with a thermally stable benzoyl group containing a typical solubility-improving CF_3_ substituent.

The polymerization results and film properties of the systems using this modifier are summarized in Table 9. *spiro*-TFBzX-DAFL showed sufficiently high polyaddition reactivity with CBDA and led to PAAs with sufficiently high *η*_red_ values, probably reflecting that its bulky side groups connected to the XAN unit did not sterically hinder the NH_2_ functional groups, related to the mutual perpendicular configuration between the XAN and FL molecular planes.

**Table 9:** Polymerization results and film properties of the CBDA- and CpODA-based PIs modified using *spiro*-TFBzX-DAFL or its CF_3_-free counterpart (*spiro*-BzX-DAFL) and related systems. The photographs at the bottom of this table show the appearance of some selected PI films (**#51C**, **#53C**, **#56R**, and **#62R**) and a precipitated solution after one-pot polymerization (**#59R**).

No.	TCDA (mol%)	Diamine (mol%)	*η*_red_ (dL/g)	Cast ^c^	*T*_400_ (%)	YI	*λ*_0_ (nm)	*T*_tot_ (%)	Haze (%)	Δ*n*_th_	*ε*_opt_	*T*_g_ (°C)	CTE (ppm/K)	*E* (GPa)	*ε*_b_ (%) ave max	*σ*_b_ (GPa)	*T*_d_^5^ (N_2_) (°C)	*T*_d_^5^ (air) (°C)
0T	CBDA	TFMB	1.63 ^a^	---	84.2	4.0	302	89.8	0.87	0.0477	2.68	345	22.9	6.57	5.2 8.0	0.19	444	432
50C	CBDA	TFMB (50) *spiro*-TFBzX-DAFL (50)	1.37 ^a^ 7.80 ^b^	DMAc 7 wt%	73.0	5.8	336	88.0	3.84	0.0414	2.79	394	15.6	6.75	3.1 5.8	0.17	426	416
51C	CBDA	TFMB (30) *spiro*-TFBzX-DAFL (70)	1.11 ^a^ 3.08 ^b^	DMAc 12 wt%	72.0	5.9	342	88.1	1.87	0.0247	2.82	390	28.5	3.50	3.9 5.4	0.10	431	422
52C	CBDA	*spiro*-TFBzX-DAFL	0.75 ^a^ 2.27 ^b^	DMAc 15 wt%	72.2	5.5	341	89.6	0.95	0.0133	2.85	378	38.3	2.75	3.8 4.7	0.073	429	421
53C ^e^	CBDA (80) CpODA (20)	TFMB (30) *spiro*-TFBzX-DAFL (70)	0.41 ^a^ 0.69 ^b^	DMAc	50.1	22.0	342	85.1	0.59	0.0177	2.83	388	32.5	1.91	3.9 6.2	0.049	410	391
54C ^e^	CBDA (70) CpODA (30)	*spiro*-TFBzX-DAFL	0.27 ^a^ 0.33 ^b^	DMAc	53.1	19.1	346	84.2	0.34	0.0080	2.85	378	48.1	--- ^d^	--- ^d^	--- ^d^	406	391
55T	CpODA	TFMB	0.83 ^a^	---	Data not available because of film cracking													
55C	CpODA	TFMB	0.71 ^a^ 0.99 ^b^	DMAc	87.0	1.7	290	90.4	0.71	0.0473	2.66	336	23.7	--- ^d^	--- ^d^	--- ^d^	473	421
55R	CpODA	TFMB	3.19	DMAc 15 wt%	85.6	1.8	292	90.1	2.73	0.0513	2.66	404	19.1	2.84	12.5 23.5	0.11	476	419
56R	CpODA	TFMB (70) *spiro*-TFBzX-DAFL (30)	1.27	DMAc	84.3	2.1	339	89.4	0.50	0.0272	2.69	399	23.6	2.86	4.0 7.1	0.080	465	431
57R	CpODA	TFMB (50) *spiro*-TFBzX-DAFL (50)	1.48	DMAc	82.1	3.4	333	88.4	1.59	0.0221	2.77	396	29.5	2.44	6.3 10.7	0.098	450	437
58R	CpODA	TFMB (30) *spiro*-TFBzX-DAFL (70)	0.96	DMAc	78.8	4.6	340	87.4	3.51	0.0171	2.81	393	32.7	2.53	3.9 6.5	0.079	453	428
59R	CpODA	*spiro*-TFBzX-DAFL	Data not available because of precipitation during one-pot polymerization															
60R	CBDA (20) CpODA (80)	TFMB (50) *spiro*-TFBzX-DAFL (50)	1.10	DMAc	81.2	2.7	346	88.5	1.07	0.0260	2.75	404	26.2	3.06	4.3 5.9	0.10	448	432
61R	CBDA (20) CpODA (80)	TFMB (30) *spiro*-TFBzX-DAFL (70)	0.84	DMAc	69.2	8.5	349	86.7	2.49	0.0169	2.80	401	34.0	2.45	2.0 4.1	0.049	446	417
62R	CpODA	TFMB (70) *spiro*-BzX-DAFL (30)	4.10	DMAc 9 wt%	81.6	3.2	323	89.4	2.65	0.0150	3.04	405	22.9	2.30	7.2 12.9	0.11	477	414
63R	CpODA	TFMB (50) *spiro*-BzX-DAFL (50)	1.56	NMP 12 wt%	52.4	15.7	331	86.4	24.2	0.0093	3.04	401	31.2	1.94	6.7 7.6	0.088	470	417

^a^ Data for PAAs. ^b^ Data for PIs prepared via Route-C. ^c^ Homogeneous PI solutions (at room temperature) with high solid contents (9–15 wt%) were used for the subsequent solution casting process, unless otherwise specified. The solutions were prepared upon soft-heating as appropriate. ^d^ Data not available because of the difficulty of the measurements due to film brittleness. ^e^ PI films prepared upon annealing at a much higher temperature (350 °C) than the standard annealing temperature (250 °C) to improve film ductility.


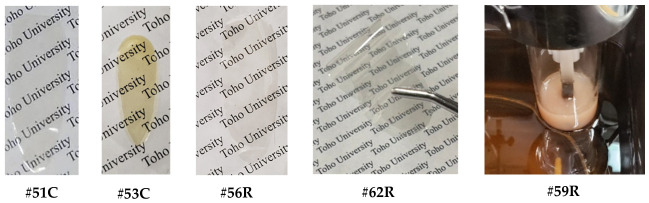


Indeed, a pronounced solubility-improving effect was observed in the CBDA/TFMB-based system modified with 50 mol% *spiro*-TFBzX-DAFL (**#50C**), as shown in Table S3, in contrast to its counterpart using *spiro*-C_8_X-DAFL (**#47C**). This undoubtedly results from effectively disturbed close main-chain stacking due to the perpendicularly arranged, bulky CF_3_-substituted benzoyl side groups. Copolymerization using *spiro*-TFBzX-DAFL also caused no significant deterioration in the film transparency. The thorough decolorization of this modifier (Figure 7) also undoubtedly contributed to this result. However, tensile testing revealed that the PI film (**#50C**) did not show a high *ε*_b_ value. Nonetheless, this film was not broken during the folding test at zero curvature radius, suggesting that it maintained the minimum-required ductility. This film (**#50C**) also achieved a very low CTE (15.6 ppm/K).

With an increase in the *spiro*-TFBzX-DAFL content from **#50C** to **#51C** and **#52C**, a gradual increase in the CTE was observed with a much smaller slope (Figure 20A(e)) than that of the above-mentioned *spiro*-C_n_X-DAFL-modified systems (Figure 20A(d)). A similar gradual increase in CTE with a further suppressed slope was also observed in another related series (from **#55R** to **#56R**, **#57R**, and **#58R**), as shown in Figure 20A(f). The magnitude of the slopes in the CTE–modifier content plots represents how easily in-plane orientation is disturbed by the modifiers. In this regard, *spiro*-TFBzX-DAFL was the most advantageous among all modifiers used in this study. If certain much bulkier side groups than the present CF_3_-substituted benzoyl group were introduced into the XAN-pendant diaminofluorenes, a crucial increase in the slope would occur.

Even though another related copolymer film (**#51C**) did not have a high *ε*_b_ value, it passed the folding test, whereas the film of **#52C** did not. The former (**#51C**) was also almost colorless (see its photograph at the bottom of Table 9) while maintaining a relatively low CTE (28.5 ppm/K) and a quite low water uptake (*W*_A_ = 0.39%) based on the less polarized CF_3_ groups present in this PI structure.

The effect of partial use of another cycloaliphatic TCDA, CpODA (Table 1 and Figure 13), was also explored with the expectation of obtaining a low CTE while avoiding a significant increase in ∆*n*_th_. This attempt is based on the notion that, compared to CBDA, a larger spacer between the functional groups in CpODA relatively lowers the polarized imide group content and can suppress main-chain electronic polarization.

However, a problem emerged; the modified version of **#51C** using 20 mol% CpODA (**#53C**) led to a PAA with a significantly decreased *η*_red_ (0.41 dL/g). Even though this film (**#53C**) was prepared via Route-C, it was appreciably colored (YI = 22.0), as evident from its photograph at the bottom of Table 9. This is because this film had to be annealed at a much higher final temperature (350 °C, see the footnote of Table 9) than the standard condition to avoid its brittleness. A further increase in the CpODA content to 30 mol% (**#54C**) resulted in a further crucial decrease in the *η*_red_ (0.27 dL/g) of the PAA, which led to a significantly colored PI film that was too brittle for subsequent tensile testing. Thus, the attempt of partial use of CpODA in the presence of CBDA was unsuccessful as long as Route-C was applied.

**Scheme 30:**
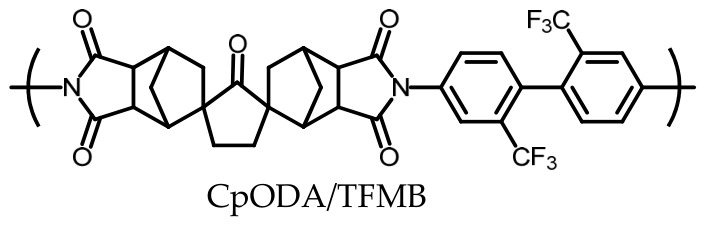
Structure of additional pristine PI system.

However, recently, we proved the significant effectiveness of the modified one-pot polymerization method (Route-R, Section 3.2 and Table 2) in producing CpODA/TFMB-based PI with an extremely high molecular weight and a resulting colorless and low-CTE cast film with sufficient toughness [48]. This suggests that CpODA/TFMB (Scheme 30) prepared via Route-R can be an additional pristine system, although film formation through Route-T (**#55T**) was hindered because of film cracking due to depolymerization emerging in an intermediate temperature range (~200 °C) during thermal imidization [48,81,82]. Therefore, to solve this issue (insufficient molecular weights), the modified one-pot polymerization method (Route-R) was applied to CpODA/TFMB-based systems using modifiers. Indeed, Route-R led to sufficiently high molecular weights (*η*_red_ >> 1.0 dL/g) of CpODA/TFMB-based PIs (**#55R**–**#58R**) and consequently, dramatically improved film toughness.

Specifically, the copolymer modified with 30 mol% *spiro*-TFBzX-DAFL (**#56R**) afforded a colorless PI film (YI = 2.1, see its photograph at the bottom of Table 9) with a low CTE and a relatively suppressed Δ*n*_th_. This film (**#56R**), as well as **#57R** with 50 mol% *spiro*-TFBzX-DAFL, passed the folding test. However, increasing the *spiro*-TFBzX-DAFL content to 70 mol% (**#58R**) made the film quite brittle without resistance to the folding test. At a *spiro*-TFBzX-DAFL content of 100 mol% (**#59R**), that is, in the homo system (CpODA/*spiro*-TFBzX-DAF), significantly deteriorated solubility was evident, as suggested by the precipitation yielded during the modified one-pot polymerization (see the appearance of the reaction mixture just after the reaction at the bottom of Table 9). This prevented the formation of homogeneous films.

To further reduce the relatively low CTE (29.5 ppm/K) of **#57R**, 20 mol% CBDA was copolymerized. The resulting system (**#60R**) still retained Route-R applicability and afforded a PI film with a slightly decreased CTE (26.2 ppm/K) with resistance to the folding test. However, a similar attempt to improve **#58R** using 20 mol% CBDA (**#61R**) was ineffective with an almost unchanged CTE and significant film brittleness (no resistance to the folding test).

#### 3.8.3. Modification Using *Spiro*-BzX-DAFL Without CF_3_ Group

As described above, the CF_3_ group in *spiro*-TFBzX-DAFL was indeed effective in improving the solubility of the resulting PIs. On the other hand, this less polarized substituent could contribute to a reduction in the overall side-group polarization, which can be disadvantageous in terms of reducing ∆*n*_th_. Therefore, the effect of the corresponding CF_3_-free modifier, *spiro*-BzX-DAFL, was investigated (Table 9). As the counterpart of the above-mentioned system (**#56R**) with low CTE, high transparency, and low Δ*n*_th_, the corresponding copolymer with 30 mol% CF_3_-free *spiro*-BzX-DAFL (**#62R**) achieved a lower Δ*n*_th_ (0.0150) than **#56R** while maintaining a low CTE (22.9 ppm/K) close to that of **#56R** in addition to high transparency with a suppressed YI of 3.2, as evident from its photograph (double-layerd loop-shaped film) at the bottom of Table 9.

On the other hand, further increasing the *spiro*-BzX-DAFL content to 50 mol% (**#63R**) obviously deteriorated the film transparency as a result of solution casting from the easy-to-color NMP solution, which was unavoidable due to its decreased solubility, in contrast to its counterpart (DMAc-cast film of the CF_3_-substituted *spiro*-TFBzX-DAFL-containing system, **#57R**). This film (**#63R**) was also highly hazy, probably owing to the same reason (its limited solubility), although it had a lower Δ*n*_th_ than **#57R** and a low CTE close to that of **#57R**.

With increasing *spiro*-BzX-DAFL content from **#55R** to **#62R** and **#63R**, the CTE gradually increased with a small slope (Figure 20A(g)), which was similar to that in the corresponding *spiro*-TFBzX-DAFL-modified systems (Figure 20A(f)). Thus, this modifier (*spiro*-BzX-DAFL), as well as *spiro*-TFBzX-DAFL, was also effective in modifying the pristine systems while suppressing the CTE increase.

#### 3.8.4. CTE–Δ*n*_th_ Correlation for PIs Modified with *Spiro*-TFBzX-DAFL and *Spiro*-BzX-DAFL

**Figure 29:**
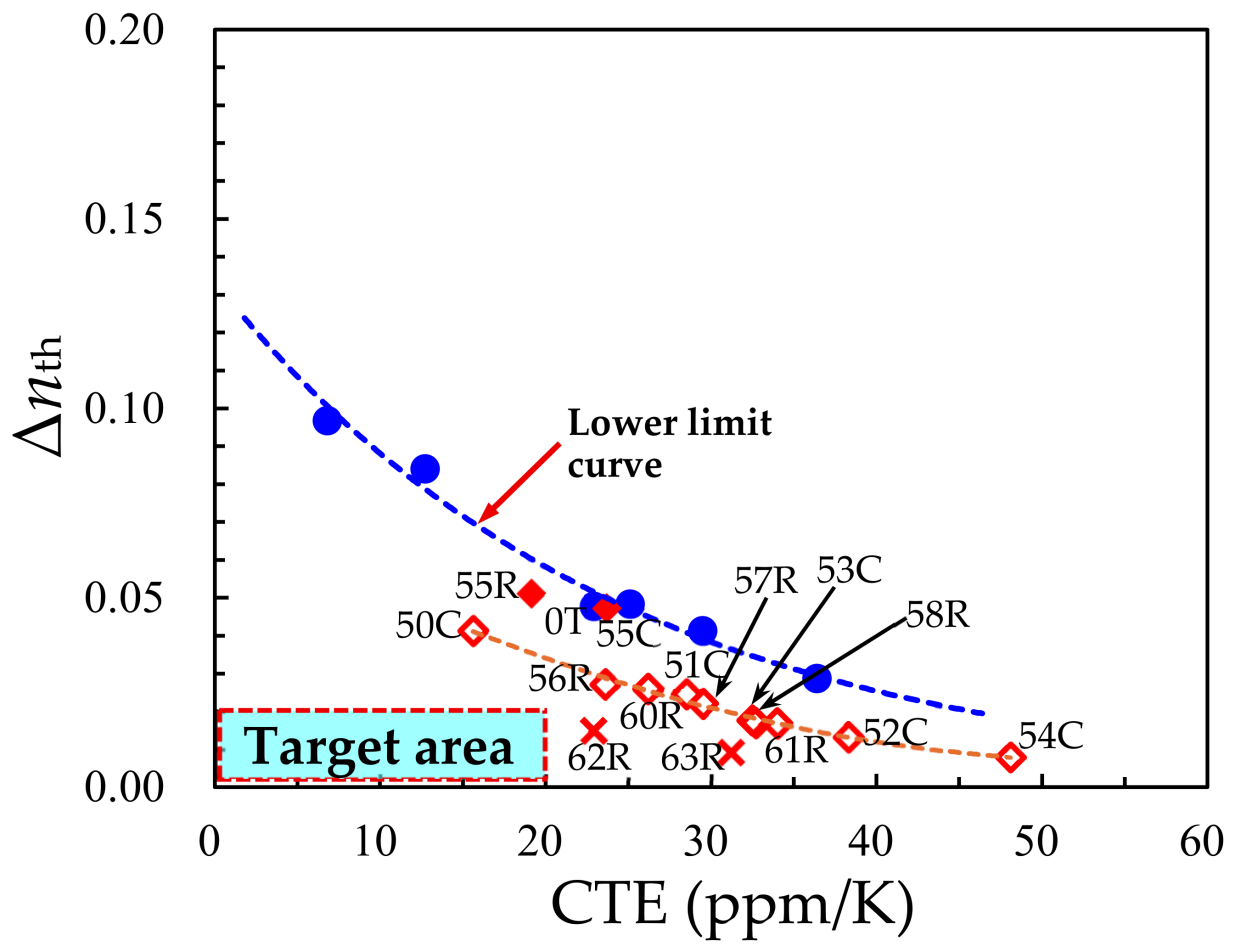
The CTE–Δ*n*_th_ relationship: (**◊**) PIs modified with *spiro*-TFBzX-DAFL (red dashed fitted curve), (**×**) PIs modified with *spiro*-BzX-DAFL, (♦) CpODA/TFMB homo PIs (**#55C**, **#55R**), and (**●**) CBDA/TFMB PI films prepared under different conditions (blue dashed fitted curve: the lower limit curve). The inserted numbers represent the sample code (Table 9).

Figure 29 shows the CTE–Δ*n*_th_ correlation diagram for the systems listed in Table 9. The plot (♦) of the CpODA/TFMB homo system (**#55R**) was located slightly below the lower limit curve, suggesting that it was optimal as the pristine system, along with CBDA/TFMB. On the other hand, it is noteworthy that most of the *spiro*-TFBzX-DAFL-modified systems provided plots (**◊**) positioned significantly below the lower limit curve, with a small data scattering on a red-fitted curve, which was shifted almost parallel to the original lower limit curve (blue dashed fitted curve), as mentioned in Section 3.3. Thus, *spiro*-TFBzX-DAFL was very effective in overcoming the trade-off between low CTE and low Δ*n*_th_.

The modification using CF_3_-free *spiro*-BzX-DAFL (particularly, **#62R** (**×**)) achieved a further enhanced effect for overcoming the trade-off compared to its counterpart using the CF_3_-subsituted modifier (**#56R**).

#### 3.8.5. Actual Z-Direction Alignment of the Side Group in Spiro-TFBzX-DAFL Unit Incorporated into the PI Main Chains

**Figure 30:**
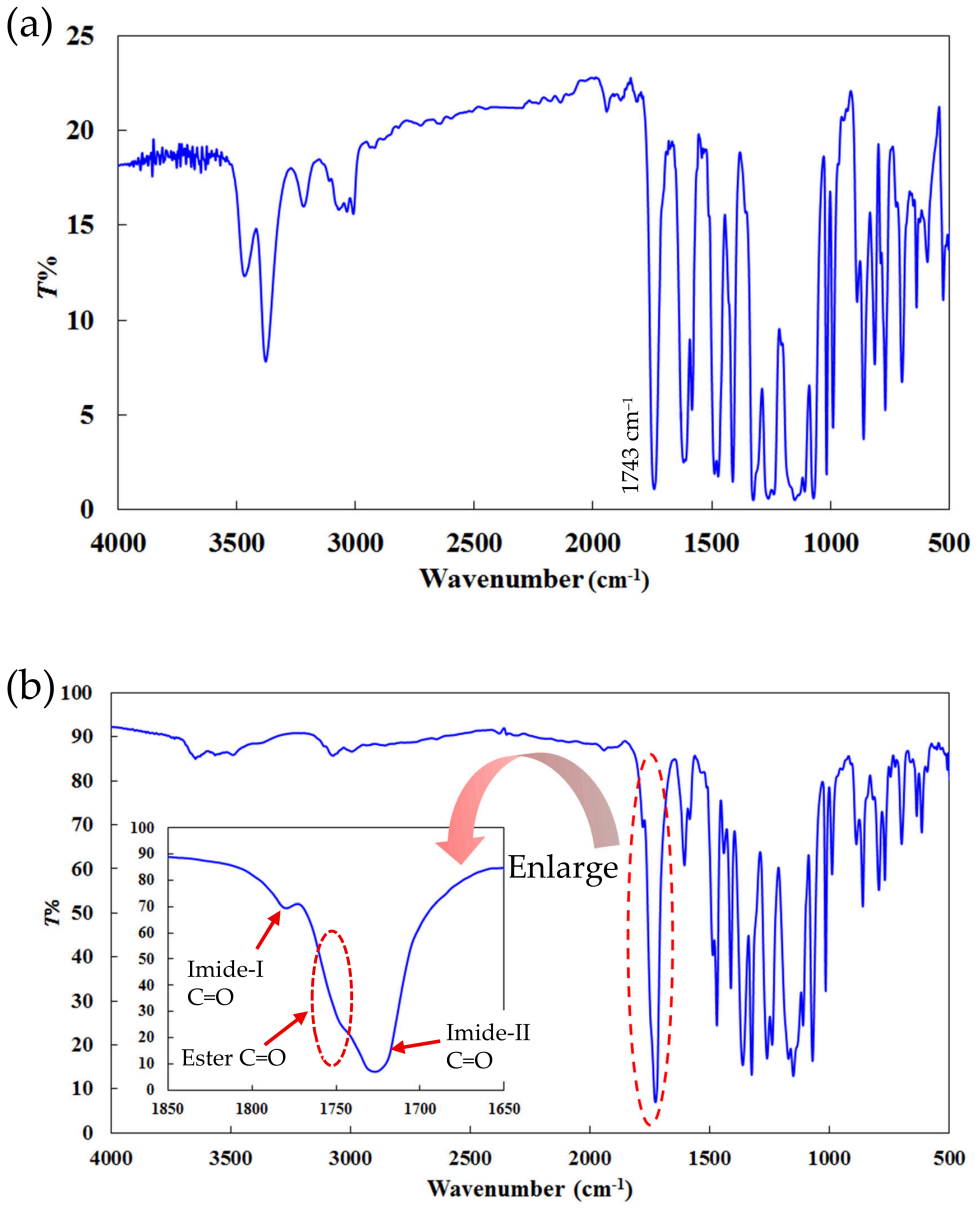
Transmission-mode non-polarized FT-IR spectra: (**a**) *spiro*-TFBzX-DAFL (KBr) and (**b**) CBDA/TFMB(50);*spiro*-TFBzX-DAFL(50) thin film (**#50C**).

The above-revealed pronounced modifier effect can be interpreted to be the result of the *Z*-direction alignment of the long axis of the side groups in the modifier units incorporated into the main chains. To verify this assumption, the polarized FT-IR spectra of a *spiro*-TFBzX-DAFL-modified PI film were measured at varying incidence angles (*θ*) with a focus on the ester C=O stretching vibration band at 1743 cm^−1^ of *spiro*-TFBzX-DAFL (Figure 30a). Figure 30b shows, as a typical example, the transmission-mode non-polarized FT-IR spectrum for a thin film of the CBDA/TFMB(50);*spiro*-TFBzX-DAFL(50) system (**#50C**). The ester C=O band at 1740 cm^−1^ appears as a shoulder of the parent peak at 1720 cm^−1^ (imide C=O), as shown in the inset of Figure 30b.

**Figure 31:**
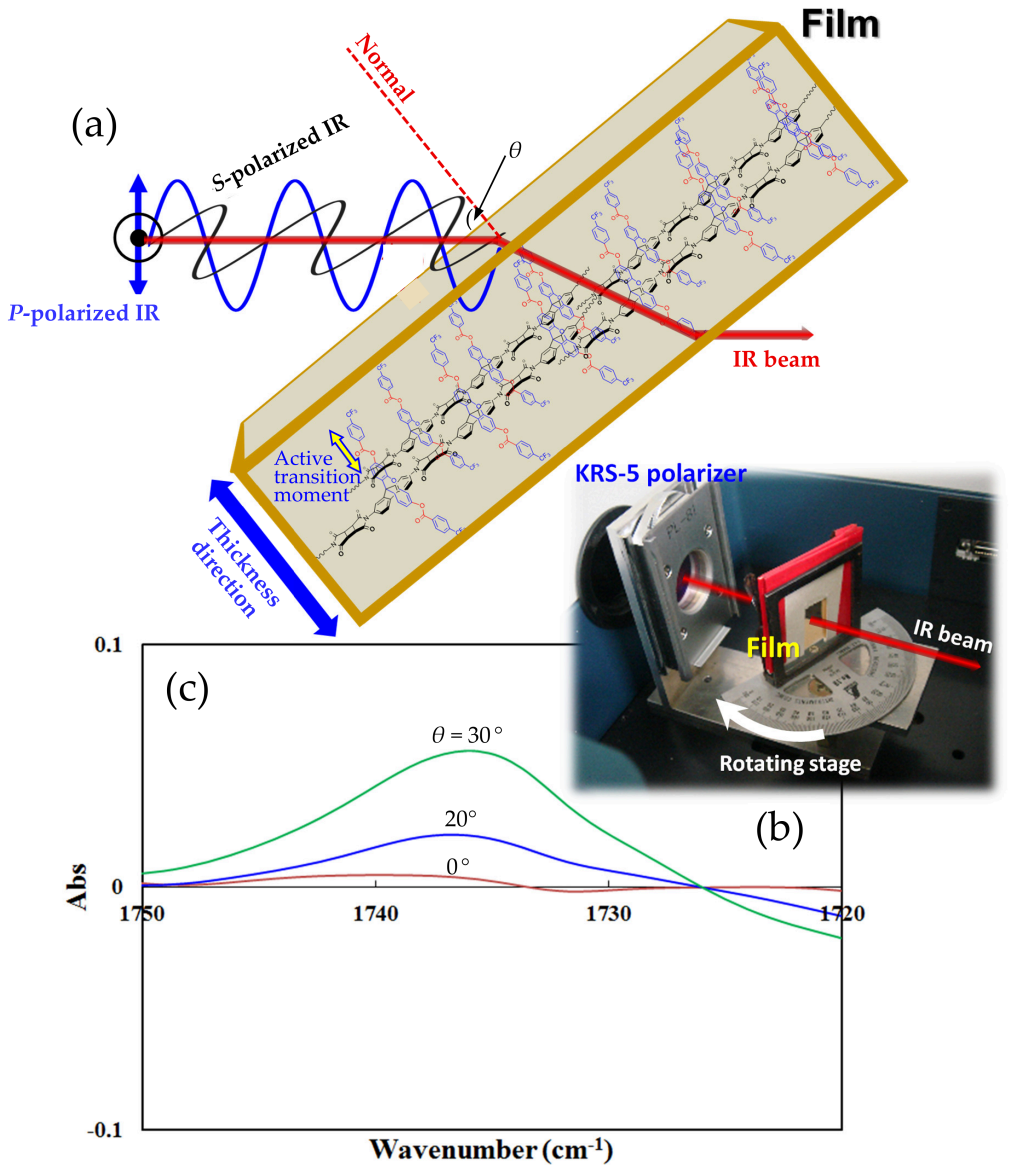
Transmission-mode polarized FT-IR spectrum measurements for the modified PI films: (**a**) A schematic drawing of the main-chain in-plane orientation, ideal vertical alignment of the side group, active transition moment (yellow arrow) of the ester C=O stretching vibration band at 1740 cm^−1^, the polarizing directions of incident IR beam, sample rotational direction, and incidence angle (*θ*); (**b**) the setup arrangement of a KRS-5 polarizer and film specimen mounted on a rotating stage; and (**c**) the difference spectra between the *P*- and *S*-polarized FT-IR spectra [normalized at 1726 cm^−1^ (imide-II band)] at different *θ* for the ester C=O band for the CBDA/TFMB(50);*spiro*-TFBzX-DAFL(50) thin film (**#50C**).

Figure 31a exhibits a schematic diagram of the in-plane-oriented PI main chains and concomitantly induced ideal *Z*-direction alignment of the side group in the *spiro*-TFBzX-DAFL unit incorporated into the PI main chains. The latter becomes more effective through the combination of the main-chain in-plane orientation and “face-on” orientation of the FL molecular planes in the modifier unit. If such side-group alignment preferentially occurred in the actual PI films, the ester C=O band at 1740 cm^−1^ is presumed to become more active to the *P*-polarized IR beam than the *S*-polarized beam at higher *θ*. This holds under the assumption that the ester C=O band at 1740 cm^−1^ has an absorption transition moment along the long axis of the side group (yellow arrow in Figure 31a). This assumption does not conflict with a previously reported result that the ester C=O stretching vibration band at around 1740 cm^−1^ of an uniaxially stretched aromatic polyester film exhibits parallel dichroism with an absorption transition moment along the main-chain direction [83]. The ester C=O band at 1755 cm^−1^ of a uniaxially oriented poly-DL-lactic acid fiber also has parallel dichroism [84]. The polarized FT-IR spectra of rotatable film specimens around the *Z*-axis were measured at different incidence angles (*θ*) using an infrared polarizer (KRS-5), as shown in Figure 31b.

Figure 31c exhibits the *θ* dependence of the difference spectra between the *P*-polarized and *S*-polarized IR absorption spectra, normalized at 1726 cm^−1^ (imide C=O band (imide-II)). The observed gradual growth of the normalized difference spectra with increasing *θ* (relative intensification of the *P*-polarized IR spectrum) suggests that a major fraction of the side groups in the *spiro*-TFBzX-DAFL unit incorporated into the PI main chains indeed align in the *Z*-direction. A similar trend in the normalized difference spectra was also observed in the CpODA/TFMB-based counterpart modified using CF_3_-free *spiro*-BzX-DAFL.

A more quantitative analysis (e.g., using the attenuated total reflection FT-IR method), which can determine the side-group orientation function, will be desired for future practical applications of the materials developed in this study. This is our upcoming subject.

### 3.9. Effect of Spiro-2367XADA

*spiro*-2367XADA is very likely more advantageous for reducing CTE than *spiro*-TFBzX-DAFL because of its completely linear mutual configuration between the two functional groups, which leads to a rod-like main-chain structure suitable for obtaining ultra-low-CTE PIs, when combined with rod-like diamines such as TFMB (Figure 27c). In addition, the combination of significant main-chain in-plane orientation and the face-on orientation of the huge and planar xanathenediimide units incorporated into the main chains ensures the vertical alignment of the long axis of the side group (FL unit in *spiro*-2367XADA). Consequently, it is expected to exhibit a significant effect in reducing both ∆*n*_th_ and CTE.

Table 10 summarizes the polymerization results and film properties of the *spiro*-2367XADA-modified systems. As **#66T** exhibited a relatively high *η*_red_ close to 1.0 dL/g, the polyaddition reactivity of *spiro*-2367XADA with diamines appears to have no crucial problems, although its *η*_red_ value is not as high as that of CBDA nor as low as that of H-PMDA (*η*_red_ = 0.25 dL/g for PAA in the H-PMDA/TFMB system [39]). However, the partial use of *spiro*-2367XADA (e.g., 30 mol% in **#64T**) did not significantly improve the originally insufficient polyaddition reactivity (low *η*_red_) of CpODA/TFMB. Consequently, the resulting PI film was cracked with appreciable coloration, as shown by its photograph at the bottom of Table 10. Applying the modified one-pot polymerization method to this system, the molecular weight and film-forming ability of the resultant PI (**#64R**) were dramatically enhanced. This modified copolymer was also highly soluble (Table S3). The DMAc-cast PI film (**#64R**) combined a high *T*_g_, high transparency, quite low CTE (20.6 ppm/K), and sufficient toughness (*ε*_b max_ = 16.0%). Optimization of the film preparation conditions afforded a PI film (**#64R′**) with a further reduced CTE (15.9 ppm/K) and somewhat improved transparency. Its appearance is shown at the bottom of Table 10. Furthermore, this PI film (**#64R′**) also passed the folding test.

**Table 10:** Polymerization results and film properties of the CpODA/TFMB-based PIs modified using *spiro*-2367XADA. The photographs at the bottom of this table show the appearance of some selected PI films (**#64T**, **#64R′**, **#65R**, and #**68R**).

No.	TCDA (mol%)	Diamine (mol%)	*η*_red_ (dL/g)	Cast ^d^	*T*_400_ (%)	YI	*λ*_0_ (nm)	*T*_tot_ (%)	Haze (%)	Δ*n*_th_	*ε*_opt_	*T*_g_ (°C)	CTE (ppm/K)	*E* (GPa)	*ε*_b_ (%) ave max	*σ*_b_ (GPa)	*T*_d_^5^ (N_2_) (°C)	*T*_d_^5^ (air) (°C)
55R	CpODA	TFMB	3.19	DMAc 15 wt%	85.6	1.8	292	90.1	2.73	0.0513	2.66	404	19.1	2.84	12.5 23.5	0.11	476	419
64T	CpODA (70) *spiro*-2367XADA (30)	TFMB	0.39	---	Data not available because of film cracking													
64R	CpODA (70) *spiro*-2367XADA (30)	TFMB	3.82	DMAc 12 wt%	72.6	7.1	360	87.9	0.58	0.0189	3.03	440 ^e^	20.6	2.45	9.5 16.0	0.10	474	421
64R′	CpODA (70) *spiro*-2367XADA (30)	TFMB	3.82	DMAc 9 wt%	74.9	6.0	360	88.4	1.01	0.0211	3.03	431	15.9	2.85	6.5 10.2	0.09	482	428
65R	CpODA (50) *spiro*-2367XADA (50)	TFMB	2.19	DMAc 14 wt%	75.1	5.0	360	88.2	0.60	0.0162	3.08	406	22.2	3.48	7.0 13.5	0.14	487	460
66T	*spiro*-2367XADA	TFMB	0.96 ^a^	---	Data not available because of obtaining free-standing film owing to film brittleness													
66R	*spiro*-2367XADA	TFMB	--- ^c^	*m*-cresol 5 wt%	36.6	14.6	340	86.2	49.9	0.0133	3.22	306 ^e^	16.4	--- ^f^	--- ^f^	--- ^f^	---	485
67R	CpODA (50) *spiro*-2367XADA (50)	TFMB (50) *spiro*-TFBzX-DAFL (50)	0.38	DMAc 30 wt%	Data not available because of obtaining free-standing film owing to film brittleness													
68R	CpODA (50) *cardo*-BPFLDA (50)	TFMB	2.58	DMAc	85.8	1.6	336	89.6	0.68	0.0275	2.77	413	33.2	2.70	6.5 10.7	0.12	498	460
69C	*cardo*-BPFLDA	TFMB	5.66 ^a^ 4.36 ^b^	DMAc	83.3	2.0	344	88.7	1.00	0.0210	2.92	389	42.9	2.17	7.3 11.7	0.11	---	---

^a^ Data for PAAs. ^b^ Data for PIs prepared via Route-C. ^c^ Data not available because the isolated PI powder was insoluble in NMP. ^d^ Homogeneous PI solutions (at room temperature) used for the solution casting process. The solutions were prepared upon warming as appropriate. ^e^ *T*_g_ determined by the TMA method. ^f^ Data not available because of the difficulty of the measurements due to film brittleness.


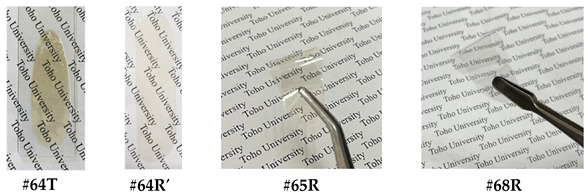


The copolymer with an increased *spiro*-2367XADA content to 50 mol% (**#65R**) also maintained a low CTE and high transparency, as shown by its photograph (double-layerd loop-shaped film) at the bottom of Table 10. Such high transparency could not have been achieved without thorough decolorization (Figure 9).

Despite a sufficiently high *η*_red_ for film formation, the *spiro*-2367XADA/TFMB homo PI film (**#66T**) prepared via Route-T was so brittle with cracks that its property evaluation was inhibited, probably owing to the afore-mentioned significant depolymerization in the intermediate temperature range. The application of Route-R in NMP to this homo system resulted in an unstable PI solution prone to gelation. The isolated PI powder was only soluble in hot *m*-cresol. Although its *m*-cresol-cast film (**#66R**) exhibited a quite low CTE (16.4 ppm/K), it was hazy, probably owing to the afore-mentioned partial precipitation during solution casting (solvent evaporation).

As described in Section 3.8.4, the CpODA/TFMB;*spiro*-TFBzX-DAFL copolymer was very effective in simultaneously achieving the low CTE and low Δ*n*_th_. Therefore, we attempted to further improve its CTE–Δ*n*_th_ relationship using *spiro*-2367XADA. However, the molecular weight of the resulting copolymer (**#67R**) did not increase (*η*_red_ = 0.38 dL/g), and consequently, it lacked film-forming ability. Thus, this attempt was unsuccessful.

The *spiro*-2367XADA-modified system was compared with its counterpart modified using 9,9-bis(3,4-dicarboxyphenyl)fluorene dianhydride (*cardo*-BPFLDA) without the ether-bridge. Specifically, the system (**#68R**) where *spiro*-2367XADA in **#65R** was replaced with *cardo*-BPFLDA exhibited a somewhat improved transparency with an obviously increased CTE (33.2 ppm/K). The latter property is probably due to a distorted chain structure at the hinge of *cardo*-BPFLDA, which contributed to disturbing the in-plane orientation during solution casting. Therefore, a further increase in the *cardo*-BPFLDA content, that is, the *cardo*-BPFLDA/TFMB homo system (**#69C**), gave rise to a further increased CTE (42.9 ppm/K). Overall, these comparative results proved the major role of the ether-bridge of *spiro*-2367XADA in lowering CTE. This homo PI film (**#69C**) also had obviously higher transparency than the corresponding homo PI film (ester-linked *cardo*-TA-BPFL/TFMB, **#11C**), likely owing to the difference in the electron-accepting ability of the phthalimide unit (originating from *cardo*-BPFLDA) and the trimellitimide unit (from *cardo*-TA-BPFL).

**Figure 32:**
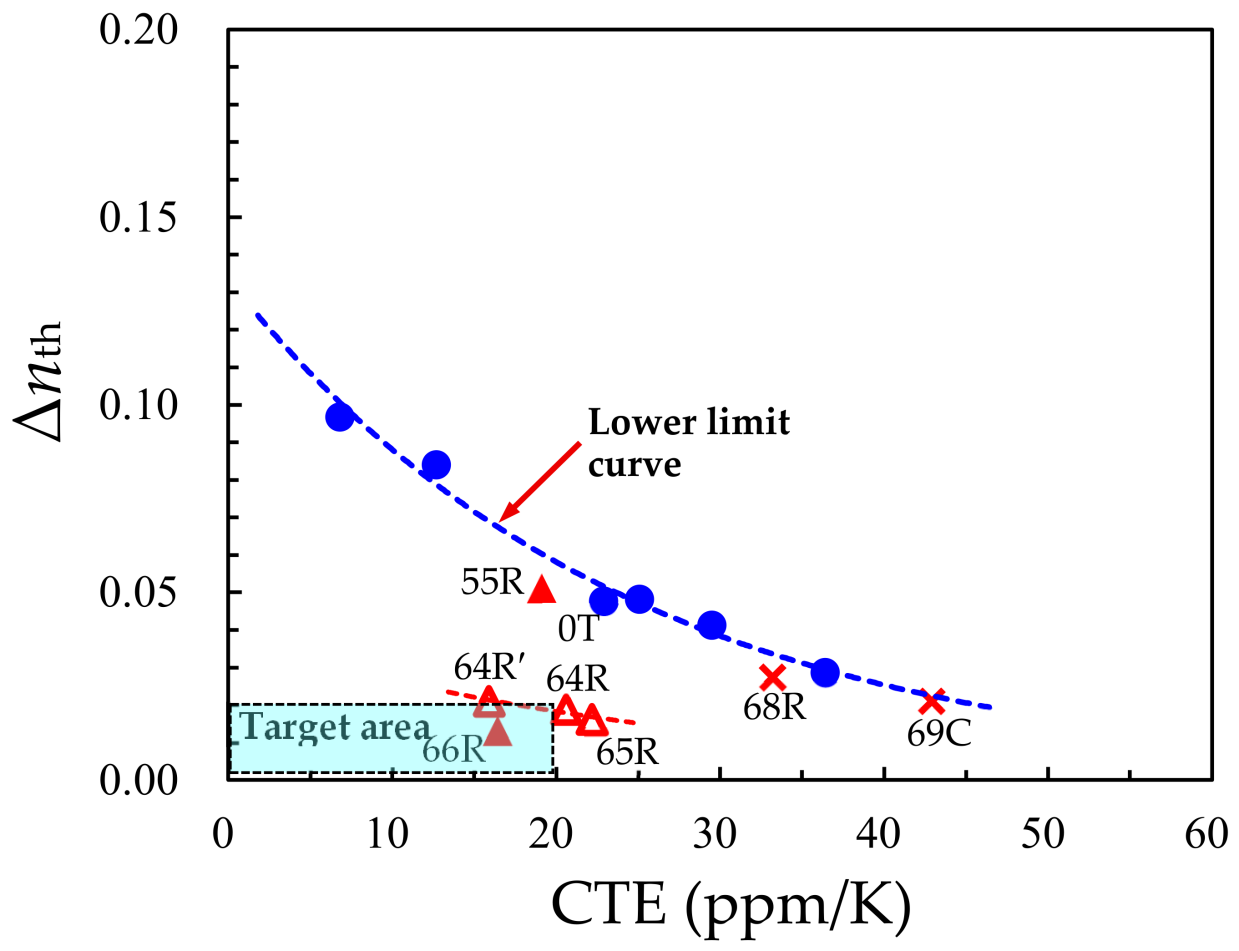
The CTE–Δ*n*_th_ relationship: (**∆**) PIs modified with *spiro*-2367XADA (**#64R**, **#64R′**, **#65R**, red dashed fitted curve), (▲) related homo PIs: CpODA/TFMB (**#55R**) and CpODA/*spiro*-2367XADA (**#66R**), (**×**) PIs using BPFLDA (**#68R**, **#69C**), and (**●**) CBDA/TFMB PI films prepared under different conditions (blue dashed fitted curve: the lower limit curve). The inserted numbers represent the sample code (Table 10).

Figure 32 shows the CTE–Δ*n*_th_ correlation diagram for the *spiro*-2367XADA-modified systems listed in Table 10. The *spiro*-2367XADA/TFMB homo PI (**#66R**, ▲) achieved the CTE–Δ*n*_th_ target. However, this film did not meet the goal in terms of transparency because of a strong haze. On the other hand, the plots of the CpODA;*spiro*-2367XADA/TFMB copolymers (**∆**, red dashed fitted curve) were located near the target area. Therefore, *spiro*-2367XADA was most effective among the modifiers examined in this study in overcoming the low-CTE–low-Δ*n*_th_ trade-off. In particular, the **#64R′** system combined a low CTE, low Δ*n*_th_, high transparency, high *T*_g_, and minimum-required film ductility. Conversely, the comparative *cardo*-BPFLDA-containing systems (**#68R**, **#69C**, **×**) were less effective, as suggested from the plots positioned near the lower limit curve.

### 3.10. Performance Balance of Selected Systems

Materials with a poor balance of the target properties are not applicable to new plastic substrates, an alternative to glass substrates, even if the trade-off between low CTE and low Δ*n*_th_ was overcome. This study emphasizes the importance of performance balance, which can be visualized using spider diagrams. The performance balance of the materials developed in this study was evaluated using five-grade evaluations, as described in our previous studies [37,39,47,48,65]. In the present paper, the detailed establishment process will be omitted. The criteria used in this study (Table 11) were established by adding a new target item, the low-Δ*n*_th_ property (abbreviated as LB), to our previous criteria. The five-grade ranking of this target item was established based on the regions in Figure 18; in this figure, most of the colorless low-CTE PIs (< 20 ppm/K) had high Δ*n*_th_ values exceeding ~0.07. Therefore, colorless PIs with Δ*n*_th_ ≥ 0.07 were assigned to the worst grade of the low-Δ*n*_th_ property (rank-1). Conversely, most of the colorless high-CTE PIs (> 60 ppm/K) exhibited Δ*n*_th_ values lower than ~0.01, as illustrated by Δ*n*_th_ = 0.0066 for the wholly cycloaliphatic CBDA/MBCHA system [67]. Accordingly, PIs with Δ*n*_th_ ≤ 0.007 were assigned as the best grade of the low-Δ*n*_th_ property (rank-5). The intermediate ranks (2–4) were established by evenly dividing the intermediate range of Δ*n*_th_ (0.007–0.07) into each intermediate rank. The evaluation criteria for other target items were established in a similar manner (the details were omitted here).

**Figure 33:**
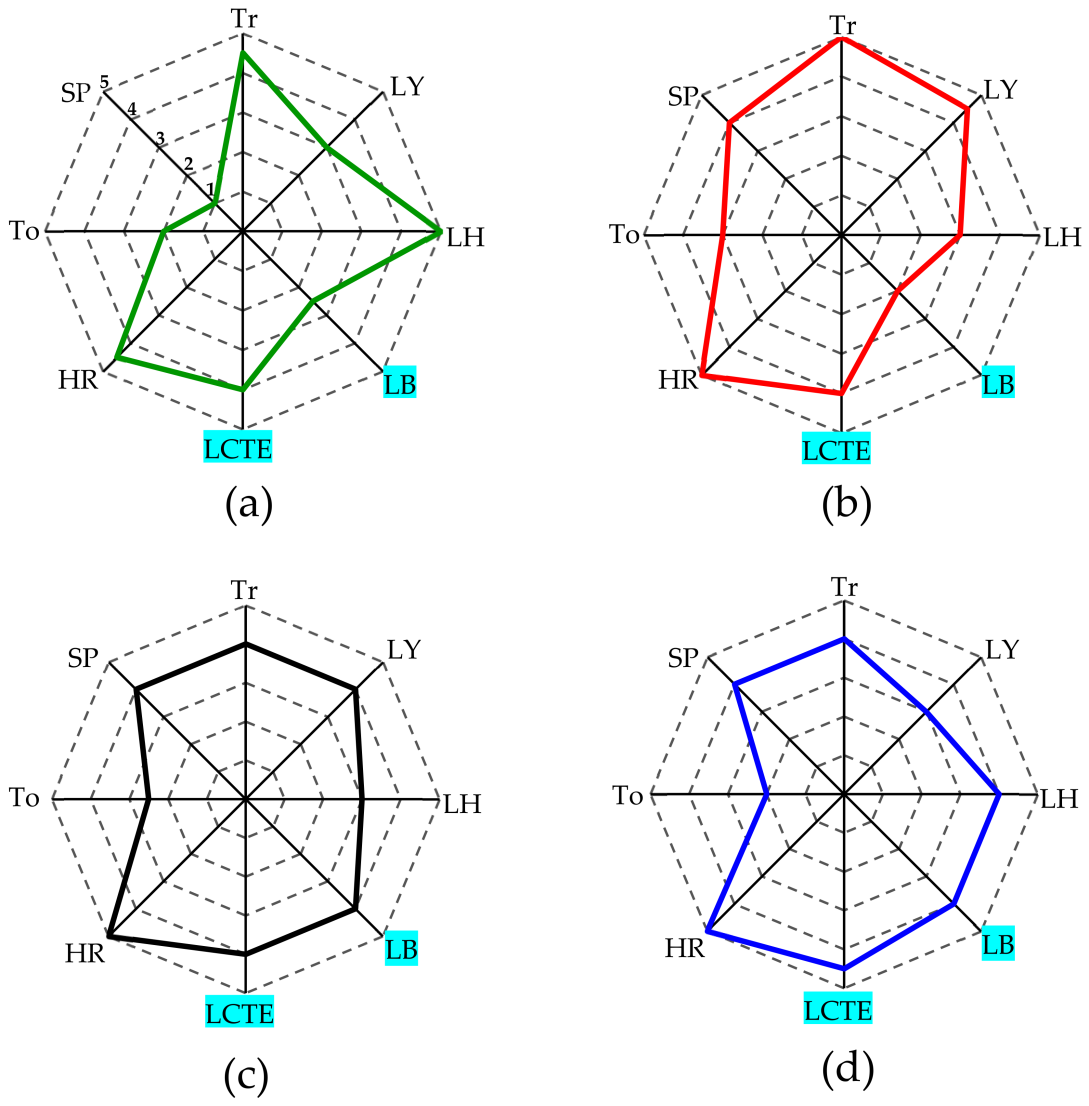
Performance balance of selected PIs examined in this study and related systems: (**a**) CBDA/TFMB (**#0T**), (**b**) CpODA/TFMB (**#55R**), (**c**) CpODA/TFMB(70);*spiro*-BzX-DAFL(30) (**#62R**), and (**d**) CpODA(70);*spiro*-2367XADA(30)/TFMB (**#64R′**). The ranking criteria of the target properties and the inserted abbreviations are shown in Table 11.

The spider diagrams for typical modified systems, along with those of the pristine systems (CBDA/TFMB and CpODA/TFMB) for comparison, are shown in Figure 33, where the focused targets in this study, low-CTE (LCTE) and low-Δ*n*_th_ (LB) properties, are highlighted in blue. A collapsed performance balance of CBDA/TFMB (**#0T**, Figure 33a) was evident from the greatly dented film toughness item (To) and solution-processability item (SP). The CpODA/TFMB homo PI system (**#55R**) showed somewhat expanded SP, but LB remained significantly dented (Figure 33b). On the other hand, the *spiro*-BzX-DAFL-modified system (**#62R**) simultaneously exhibited significantly expanded LCTE and LB, but with dented To (Figure 33c). Further expanded LCTE and LB were observed in the *spiro*-2367XADA-modified system (**#64R′**) while it satisfactorily achieved other targets, although there was still room for improvement of To, as shown in Figure 33d.

Thus, this study successfully developed unique PI films applicable to plastic substrates using well-designed *spiro*-type modifier monomers that simultaneously achieve a low CTE and low Δ*n*_th_ in addition to a very high *T*_g_, sufficiently high thermal stability, excellent optical transparency, good solubility, and minimum-required ductility without the use of fillers.

In this study, we focused on only the “initial” properties of the developed materials. However, there is another important property for practical applications to various image display devices, namely, durability against light from backlights or light-emitting elements. The durability of our materials is currently under investigation.

## 4. Conclusions

This study developed unique materials applicable to plastic substrates for use in flexible-display devices that overcome the low-CTE–low-Δ*n*_th_ trade-off relationship, in addition to achieving a very high *T*_g_, sufficiently high thermal stability, excellent optical transparency, good solubility, and minimum-required ductility.

The CBDA/TFMB PI films prepared under different conditions provided a clear lower boundary (lower limit curve) in the CTE–Δ*n*_th_ relationship. This study endeavored to overcome the trade-off between low CTE and low Δ*n*_th_, that is, to significantly exceed the lower limit curve toward our target region of CTE ≤ 20 ppm/K and Δ*n*_th_ ≤ 0.02, while simultaneously achieving other important properties. One of the keys to achieving the present goal was compatibility with Route-C or Route-R (high solubility of the PIs) because these manufacturing routes are more advantageous for reducing CTE and enhancing film transparency than conventional Route-T.

As our initial attempt, the pristine CBDA/TFMB system was modified by copolymerization with a thermotropic liquid-crystalline diamine, 35DAB-BPC_12_. However, this modification caused a significant increase in the CTE. Thus, this modifier was less effective in exceeding the lower limit curve, except for a slight positive effect observed in **#4C**.

The modification effects of a variety of *cardo*-type monomers were investigated. This type of modifiers significantly improved the solubility, which consequently enhanced the applicability of Route-C. Nonetheless, most of the modified systems were ineffective in exceeding the lower limit curve. Only **#13** exhibited a slight positive effect.

Ether-linked *spiro*-type monomers were used to modify CBDA/TFMB to significantly improve the film toughness of the resulting copolymers. However, this attempt was effective neither at improving the film toughness nor overcoming the trade-off. A slight positive effect on the latter was observed only in **#28T**. On the other hand, when using ester-linked *spiro*-type diamines, a certain positive effect in exceeding the lower limit curve was observed in **#38C**.

The modification of CBDA/TFMB using *spiro*-C_n_X-DAFL was less effective at improving solubility. The resulting copolymers showed appreciably deteriorated film transparency, probably owing to the presence of the less thermally stable alkyl groups in these modifiers. The system that had a slight positive effect on overcoming the trade-off was limited to **#47C**.

When CBDA/TFMB and/or CpODA/TFMB were modified using *spiro*-TFBz-DAFL, which contains thermally stable CF_3_-substituted benzoyl side groups, a prominent effect on overcoming the trade-off was observed (particularly in **#56R**). The use of its CF_3_-free counterpart (*spiro*-Bz-DAFL) was more effective (particularly in **#62R**). Polarized FT-IR difference spectra measured at different incidence angles for these copolymer films suggested that these side groups align in the *Z*-direction, corresponding to the observed prominent effect.

When using FL-pendant XAN-type TCDA, *spiro*-2367XADA, the highest effect on overcoming the trade-off among the modifiers examined in this study was observed (particularly in **#64R′**). This PI film also combined other excellent target properties.

Overall, this study successfully developed unique materials applicable to plastic substrates using well-designed *spiro*-type modifiers, which overcame the trade-off between low CTE and low Δ*n*_th_ without the help of any fillers, while also achieving other target properties, including a very high *T*_g_, sufficiently high thermal stability, excellent optical transparency, and good solubility, although there was still room for improvement regarding film toughness.

## Figures and Tables

**Table 1:** Abbreviations, sources, vacuum-drying conditions, and melting points of the commercially available monomers used in this study.

Monomer	Source	Vacuum-Drying Condition	Melting Point (°C)
1,2,3,4-Cyclobutanetetracarboxylic dianhydride (CBDA)	Nissan Chemical	180 °C/12 h	241 ^a^
Norbornane-2-*spiro*-*α*-cyclopentanone-*α*′-*spiro*-2″-norbornane-5,5″,6,6″-tetracarboxylic dianhydride (CpODA)	ENEOS	160 °C/12 h	300 ^a^
1*S*,2*S*,4*R*,5*R*-Cyclohexanetetracarboxylic dianhydride (H′-PMDA)	Iwatani Industrial Gases	150 °C/24 h	274 ^a^
9,9-Bis(3,4-dicarboxyphenyl)fluorene dianhydride (*cardo*-BPFLDA)	JFE Chemical	160 °C/12 h	271 ^b^
2,2′-Bis(trifluoromethyl)benzidine (TFMB)	Wakayama Seika	50 °C/12 h	184 ^a^
*Trans*-1,4-cyclohexanediamine (*t*-CHDA)	Iwatani Industrial Gases	30 °C/24 h	70 ^a^

^a^ *T*_m_ determined from the endothermic peak temperature measured at 5 °C/min on DSC. ^b^ *T*_m_ from the safety data sheets.

**Table 2:** The features of three polymerization/film preparation processes applied in this study.

Route	Polymerization Process	Casting Process	PI Solubility	High Monomer Reactivity for Film Formation	Heating Temperature Range for PI Film Formation (°C)	To Obtain Colorless PI Films for Soluble Semi-Aromatic Systems	To Reduce CTE for Soluble Semi-Aromatic Systems
T	PAA (without cat.)	PAA solution	Needless	Essential	300–350	Unfavorable	Often unfavorable
C	PAA (without cat.)	PI solution	Essential	Essential	200–250	Most effective	Effective in some cases
R	PI (with catalysts)	PI solution	Essential	Desired, even if not available, compensable with cat. and heating	200–250	Applicable	Effective in some cases

**Table 7:** The superiority/inferiority of the *cardo*- and *spiro*-type modifiers in terms of improving the selected properties in a comparison of the CBDA/TFMB(50);modifier(50) systems.

	Compared Systems and Superiority		
Properties	Ester-Linked *Spiro*-(#38C) vs. Ester-Linked *Cardo* Modifiers (#13C)	Ester-Linked *Spiro*-(#38C) vs. Ether-Linked *Spiro*-Modifiers (#20C)	Ester-Linked *Spiro*-(#38C) vs. Ester-Linked *a*-*Spiro*-Modifiers (#43C)
Solubility	38C ≥ 13C	38C >> 20C	38C > 43C
Film transparency	13C ≥ 38C	38C > 20C	38C ≥ 43C
Heat resistance (*T*_g_)	38C > 13C	38C ≥ 20C	38C ≥ 43C
Low-CTE properties	38C ≥ 13C	38C > 20C	43C > 38C
Film toughness	38C ≈ 13C	38C ≈ 20C	38C > 43C

**Table 11:** The criteria established for a five-grade ranking of the achievement level for each target property.

Property	Parameter	Ranking				
		1	2	3	4	5
Physical heat resistance (HR)	*T*_g_ (°C)	≤200	220–240	250–270	280–300	≥360 or unclear *T*_g_ on DMA
Low CTE (LCTE)	CTE (ppm/K)	≥70	60–50	45–35	30–20	≤10
Low birefringence (LB)	Δ*n*_th_	≥0.07	0.06–0.05	0.04–0.03	0.02–0.01	≤0.007
Light transmittance (Tr)	*T*_400_ (%)	≤5	20–30	40–60	70–80	≥85
Low yellowness (LY)	YI	≥20	10–7	6–4	3–2	≤1
Low haze (LH)	Haze (%)	≥10	4–3	3–2	2–1	≤1
Toughness (To)	*ε*_b max_ (%)	≤2 or no film-forming ability	5–10	20–30	40–60	≥80
Solution- processability (SP)	Qualitative solubility	Insoluble	Soluble in amide solvents (≤1 wt%)	Soluble in amide solvents (3–5 wt%)	Soluble in amide solvents (>~10 wt%)	Soluble in non-amide solvents (>~10 wt%)

## Data Availability

The original contributions presented in this study are included in the article/Supplementary Materials. Further inquiries can be directed to the corresponding author.

## Supplementary Materials

The following supporting information can be downloaded at https://www.mdpi.com/article/10.3390/polym18091108/s1: Table S1: Abbreviations and melting points of the raw materials used in this study. Figure S1: Reaction schemes for the synthesis of *cardo*-type monomers (*cardo*-TA-BPFL, *cardo*-AB-BPFL, and *cardo*-AP-BPFL). Document S1. Scheme S1: Molecular structure of *cardo*-TA-BPFL with numbering. Document S2. Scheme S2: Molecular structure of *cardo*-NB-BPFL with numbering. Scheme S3: Molecular structure of *cardo*-AB-BPFL with numbering. Document S3. Scheme S4: Molecular structure of *cardo*-NP-BPFL with numbering. Scheme S5: Molecular structure of *cardo*-AP-BPFL with numbering. Figure S2: GPC curve for CBDA(50);*spiro*-TA-FLX(50);TFMB copolymer. Figure S3: Incompatibility with Route-C and Route-R for the CBDA/TFMB system: (a) appearance of gelation during chemical imidization in DMAc and (b) a viscous and inhomogeneous reaction mixture just after one-pot polymerization in NMP. Figure S4: DSC thermogram of 35DAB-BPC12 (a) and polarizing optical microscope (POM) photograph taken at 172.8 °C during the heating process (LC ranges: 172.8–178.4 °C during the heating process, 176.4–165.5 °C during the cooling process) (b) and a typical stacked structure between the mesogenic units (c). Figure S5: A schematic drawing of expected vertical alignment for the 35DAB-BPC12 unit connected to the CBDA-based PI chains. Table S2: Results of qualitative solubility test at about 1 wt% using PI powder samples obtained via Route-C or Route-R. Figure S6: A schematic drawing for disturbed vertical alignment due to internal rotation at the ester group in the 35DAB-BPC12 unit incorporated into the PI main chains. Figure S7: Possible intramolecular (through-bond) electron donor–acceptor interactions generated in the CBDA/TFMB-based PI systems using TCDA modifiers: (a) *cardo*-TA-BPFL and (b) *spiro*-TA-FLX. Document S4. Scheme S6: Structure of AB-TFMB with numbering. Figure S8: Schematically depicted main-chain linearity of the extended forms (red arrows) for isomeric PIs: (a) PMDA/4,4′-ODA and (b) PMDA/3,4′-ODA. Table S3: Results of qualitative solubility test at about 1 wt% using PI powder samples obtained via Route-C or Route-R.
