# Supplementary material for: Colorless Polyimides Derived from Novel Role-Dividing Spiro-Type Monomers: Strategies to Overcome the Trade-Off Between Low Linear Coefficients of Thermal Expansion and Low Thickness-Direction Birefringence Without Fillers
Source: Polymers (Basel). 2026 Apr 30;18(9):1108. doi: 10.3390/polym18091108 (PMC13165796; doi:10.3390/polym18091108)
Supplement: Supplementary file 1 [file polymers-18-01108-s001.zip › polymers-4241928-supplementary.pdf]

Supplementary Materials

Table S1. Abbreviations and melting points of the raw materials used in this study.

Raw material	Source	Melting point (°C)
9,9-Bis(4-hydroxyphenyl)fluorene (BPFL)	TCI	227 [a]
9,9-Bis(4-hydroxy-3-methylphenyl)fluorene (BCFL)	TCI	219 [a]
9-Fluorenone (9FLN)	TCI	84 [a]
2,7-Dinitro-9-fluorenenone (27DNFLN)	TCI	298 [a]
4,4′-Bipnenol (44BP)	TCI	281 [a]
Resorcinol (RC)	Wako Chemical	111 [a]
Hydroquinone (HQ)	Wako Chemical	173 [b]
3,4-Dimethylphenol (34DMP)	TCI	66 [a]
1-Bromohexane (BrHx)	TCI	–85 [a]
1-Bromododecane (BrDD)	TCI	---
1-Bromo-2-ethylhexane (Br2EH)	TCI	---
4-Fluoronitrobenzene (4FNB)	Wako Chemical	21 [a]
3-Fluoronitirobenzene (3FNB)	Wako Chemical	---
1-Chloro-4-nitro-2-(trifluoromethyl)benzene (1C4NTFB)	Wako Chemical	22 [a]
3,5-Dinitrobenzoyl chloride (35DNBC)	TCI	69 [a]
Trimellitic anhydride chloride (TMAC)	TCI	69 [b]
4-Nitrobenzoyl chloride (4-NBC)	TCI	73 [a]
4-(Trifluoromethyl)benzoyl chloride (4TFBC)	TCI	---
Benzoyl chloride (BzC)	TCI	---

[a] Data from the safety data sheets.

[b] Data determined from the endothermic peak temperatures in DSC thermograms recorded at a heating rate of 5 °C/min.

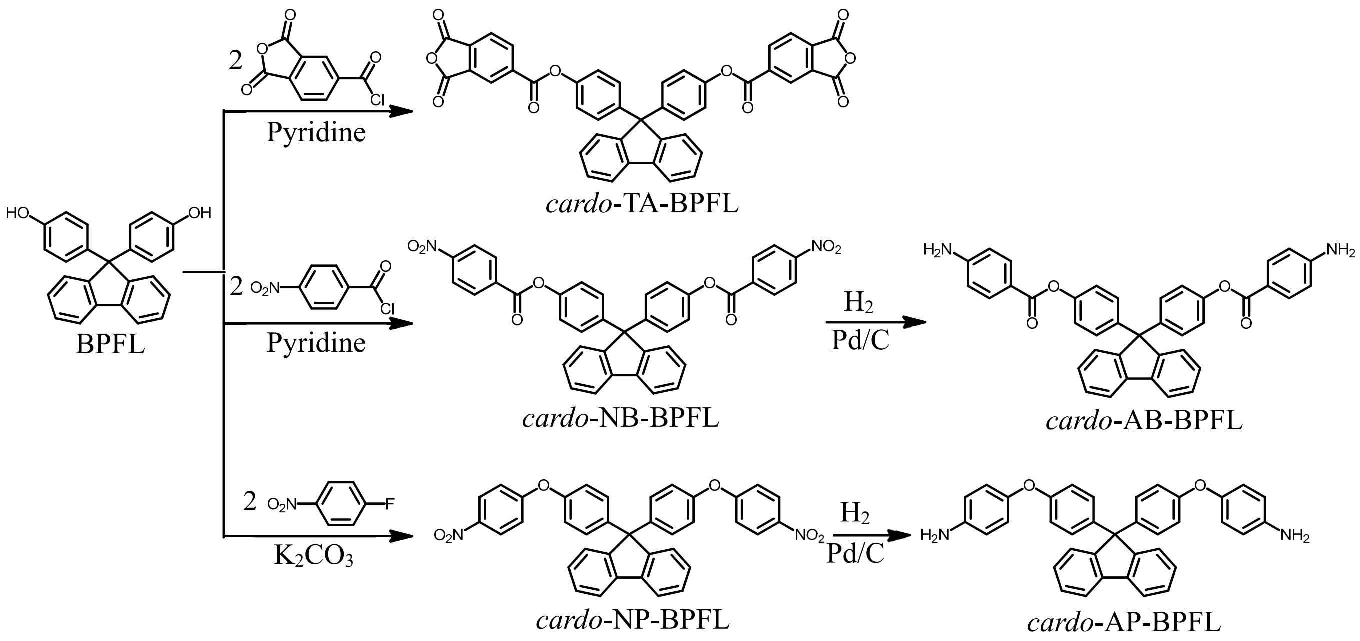

Figure S1. Reaction schemes for the synthesis of *cardo*-type monomers (*cardo*-TA-BPFL, *cardo*-AB-BPFL, and *cardo*-AP-BPFL).

Document S1. A *cardo*-type ester-linked tetracarboxylic dianhydride (TCDA), *cardo*-TA-BPFL, was synthesized according to the reaction scheme (**Figure S1**) as follows. Trimellitic anhydride chloride (TMAC, 60 mmol) was dissolved in anhydrous tetrahydrofuran (THF, 28 mL) in a septum cap-sealed flask. In another septum cap-sealed flask, 9,9-bis(4-hydroxyphenyl)fluorene (BPFL, 30 mmol) was dissolved in THF (50 mL) in the presence of pyridine (90 mmol, 7.2 mL). The BPFL solution was gradually added to the TMAC solution kept at 0 °C with a syringe with continuous magnetic stirring for 2 h, and the reaction mixture was stirred for several hours and additionally at room temperature for 12 h. After the pyridine–HCl salt formed was removed by filtration, the excess of THF was removed using an evaporator. The pale-yellow solid obtained was vacuum-dried at 120 °C for 12 h. The crude product was first dissolved in acetic anhydride at 80 °C and kept at room temperature for 12 h. This procedure caused the formation of white precipitate in acetic anhydride. The product collected by filtration was washed with *n*-hexane and vacuum-dried at 100 °C. The product was recrystallized from an anhydrous mixed solvent [1,4-dioxane/toluene (1/1, v/v)]. The needle-like white crystal formed was collected by filtration and dried at 120 °C under vacuum for 12 h (yield: 77%).

The molecular structure of this product (Scheme S1) was confirmed by the following data. T_m = 294 ºC (DSC). FT-IR (KBr plate method, cm^{-1}): 3065 (C_{arom}–H), 1863/1782 (acid anhydride, C=O), 1742 (ester, C=O), 1502 (1,4-phenylene group), and the absence of the absorption bands at 3400–3500 cm^{-1} (O–H) from the unreacted BPFL and ~2600 cm^{-1} (hydrogen-bonded carboxylic acid O–H) from the hydrolyzed by-product. ^{1}H-NMR [400 MHz, DMSO-d_6, δ, ppm]: 8.62–8.57 [m, 4H (4.00H), 3,3′- + 5,5′-protons of the phthalic anhydride (PAn) unit], 8.27 [d, 2H (2.04H), J = 8.0 Hz, 6,6′-protons of PAn], 7.99 [d, 2H (2.17H), J = 8.0 Hz, 4,5-protons of fluorene (FL) unit], 7.56 [d, 2H (2.00H), J = 8.0 Hz, 1,8-protons of FL], 7.45 [t, 2H (2.02H), J = 7.4 Hz, 3,6-protons of FL], 7.38 [t, 2H (2.03H), J = 7.5 Hz, 2,7-protons of FL], 7.12 [m, 8H (8.06H), 3,3′,5,5′- + 2,2′,6,6′-protons of the central phenoxy (PhO) group]. Elemental analysis: Calcd. (%) for $C_{43}H_{22}O_{10}$ (698.64 g/mol): C, 73.93; H, 3.17. Found: C, 73.71; H, 3.31.

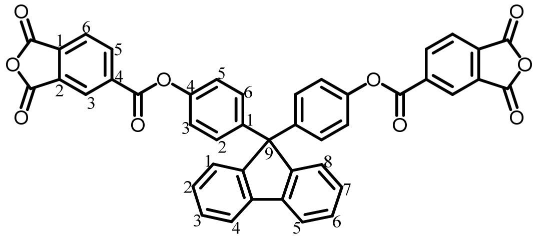

Scheme S1. Molecular structure of *cardo*-TA-BPFL with numbering.

Document S2. A *cardo*-type ester-linked diamine (*cardo*-AB-BPFL) was synthesized according to the reaction scheme (Figure S1) through the following intermediates.

cardo-NB-BPFL. BPFL (10.3 mmol) was dissolved in anhydrous *N*,*N*-dimethylformamide (DMF, 21.8 mL) in the presence of pyridine (5.0 mL, 62.1 mmol) in a septum cap-sealed flask. In another septum cap-sealed flask, 4-nitrobenzoyl chloride (4NBC, 31.1 mmol) was dissolved in anhydrous DMF (8.5 mL). The 4NBC solution was gradually added to the BPFL solution kept at 0 °C with a syringe with continuous magnetic stirring. The reaction mixture was stirred for several hours at 0 °C and additionally at room temperature for 12 h. The yellow precipitate yielded was collected by filtration and washed with DMF and water. The crude product was recrystallized from 1,4-dioxane. The pale yellowish fine needle-like crystal formed was collected by filtration and dried at 100 °C under vacuum for 12 h (yield: 81%).

The molecular structure of this product (Scheme S2) was confirmed by the following data. T_m = 287 °C (DSC). FT-IR (KBr plate method, cm^{-1}): 3115/3058 (C_{arom}–H), 1742 (ester, C=O), 1526/1349 (NO_2), and the absence of the absorption bands at 3400–3500 cm^{-1} (O–H) from the unreacted BPFL. ^{1}H-NMR [400 MHz, DMSO-d_6, δ, ppm]: 8.41 [d, 4H (4.01H), J = 8.7 Hz, 2,2′,6,6′-protons of the nitrobenzene (NB) unit], 8.33 [d, 4H (4.04H), J = 8.8 Hz, 3,3′,5,5′-protons of NB], 7.99 [d, 2H (2.00H), J = 7.5 Hz, 4,5-protons of FL], 7.55 [d, 2H (2.07H), J = 7.6 Hz, 1,8-protons of FL], 7.46 [t, 2H (2.03H), J = 7.2 Hz, 3,6-protons of FL], 7.38 [t, 2H (2.01H), J = 7.5 Hz, 2,7-protons of FL], 7.29–7.24 [m, 8H (8.07H), 3,3′,5,5′- + 2,2′,6,6′-protons of PhO].

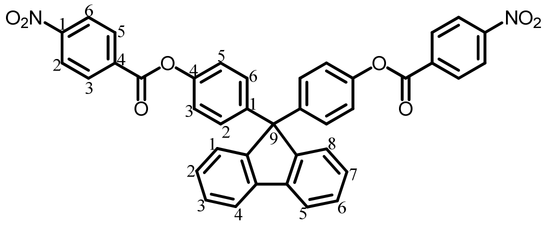

Scheme S2. Molecular structure of *cardo*-NB-BPFL with numbering.

cardo-AB-BPFL. The nitro groups of *cardo*-NB-BPFL were reduced as follows. In a three-neck flask, *cardo*-NB-BPFL (7.08 mmol) was dissolved in DMF (50 mL) in the presence of Pd/C (0.46 g) as a catalyst. The reaction mixture was refluxed at 80 °C for 12 h in a hydrogen atmosphere. After the catalyst residue was removed by hot filtration, the filtrate was concentrated using an evaporator and poured into a large quantity of *n*-hexane. The white precipitate formed was collected by filtration and dried at 100 °C for 12 h under vacuum (yield: 84%). The crude product was purified by recrystallization from a mixed solvent (1,4-dioxane/toluene, 1/6, v/v).

The molecular structure of this product (Scheme S3) was confirmed by the following data. T_m = 281 °C (DSC). FT-IR (KBr plate method, cm^{-1}): 3477/3373/3221 (NH_2, N–H stretching vibration), 3038 (C_{arom}–H), 1709 (ester, C=O), 1620 (NH_2, deformation + FL, group in FL),

1517/1501 (1,4-phenylene group). ^{1}H-NMR [400 MHz, DMSO-d_6, δ, ppm]: 7.97 [d, 2H (2.00H), J = 7.2 Hz, 4,5-protons of FL], 7.75 [d, 4H (4.09H), J = 8.8 Hz, 3,3′,5,5′-protons of the aniline (AN) unit], 7.51 [d, 2H (1.93H), J = 7.6 Hz, 1,8-protons of FL], 7.44 [t, 2H (2.06H), J = 7.0 Hz, 3,6-protons of FL], 7.36 [t, 2H (2.03H), J = 7.4 Hz, 2,7-protons of FL], 7.18 [d, 4H (3.91H), J = 8.8 Hz, 3,3′,5,5′-protons of PhO], 7.11 [d, 4H (3.91H), J = 8.8 Hz, 2,2′,6,6′-protons of PhO], 6.61 [d, 4H (3.94H), J = 8.8 Hz, 2,2′,6,6′-protons of AN], 6.17 [s, 4H (3.98H), NH_2]. Elemental analysis: Calcd. (%) for $C_{39}H_{28}O_4N_2$ (588.66 g/mol): C, 79.58; H, 4.79; N, 4.76, Found: C, 79.38; H, 5.00; N, 4.67.

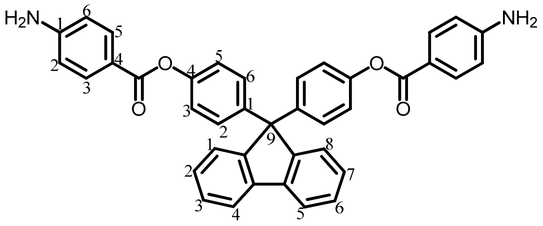

Scheme S3. Molecular structure of *cardo*-AB-BPFL with numbering.

Dimethyl substituted *cardo*-AB-BPFL, *cardo*-AB-BCFL, was synthesized by replacing BPFL with 9,9-bis(4-hydroxy-3-methylphenyl)fluorene (BCFL) in a similar manner.

Document S3. A *cardo*-type ether-linked diamine (*cardo*-AP-BPFL) was synthesized according to the reaction scheme (Figure S1) through the following intermediates.

cardo-NP-BPFL. In a three-neck flask, 4-fluoronitrobenzene (4FNB, 31.19 mmol) was dissolved in anhydrous DMF (25.5 mL) in the presence of K_2CO_3 (2.488 g, and the reaction mixture was maintained at 120 °C. To obtain *cardo*-NP-BPFL (Scheme 4), to this solution, BPFL (15.09 mmol) dissolved in DMF was added over 20 min with a separating funnel, and the reaction mixture was refluxed at 120 °C for 4 h in a nitrogen atmosphere. The pale-yellowish precipitate formed was collected by filtration, washed with DMF and water, and dried at 100 °C for 12 h under vacuum (yield: 78%).

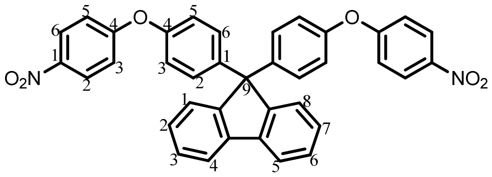

Scheme S4. Molecular structure of *cardo*-NP-BPFL with numbering.

cardo-AP-BPFL. The nitro groups of *cardo*-NP-BPFL were reduced as follows. In a three-neck flask, *cardo*-NP-BPFL (9.95 mmol) was dissolved in 1,4-dioxane (70 mL) in the presence of Pd/C (0.587 g). The reaction mixture was refluxed at 80 °C for 12 h in a hydrogen atmosphere. After the residual Pd/C was removed by hot filtration, the filtrate was concentrated using an evaporator and gradually poured into a large quantity of water. The white precipitate was collected by filtration, washed with methanol, and dried at 100 °C for 12 h under vacuum, followed by recrystallization from toluene (yield: 73%).

The molecular structure of this product (Scheme S5) was confirmed by the following data. T_m = 175 °C (DSC). FT-IR (KBr plate method, cm^{-1}): 3446/3369/3217 (NH_2, N–H stretching vibration), 3036 (C_{arom}–H), 1619 (NH_2, deformation + biphenyl group in FL), 1497 (1,4-phenylene group), 1233/1170 (C_{arom}–O–C_{arom}). ^{1}H-NMR [400 MHz, DMSO-d_6, δ, ppm]: 7.90 [d, 2H (2.00H), J = 7.6 Hz, 4,5-protons of FL], 7.41–7.36 [m, 4H (3.99H), 3,6- + 1,8-protons of FL], 7.30 [t, 2H (1.90H), J = 7.5 Hz, 2,7-protons of FL], 7.02 [d, 4H (4.00H), J = 8.9 Hz, 3,3′,5,5′-protons of AN], 6.73–6.71 [m, 8H (7.86H), 2,2′,6,6′- + 3,3′,5,5′-protons of PhO], 6.55 [d, 4H (4.01H), J = 8.8 Hz, 2,2′,6,6′-protons of AN], 4.96 [s, 4H (3.98H), NH_2]. Elemental analysis: Calcd. (%) for $C_{37}H_{28}O_2N_2$ (532.64 g/mol): C, 83.43; H, 5.30; N, 5.26, Found: C, 83.30; H, 5.21; N, 5.10.

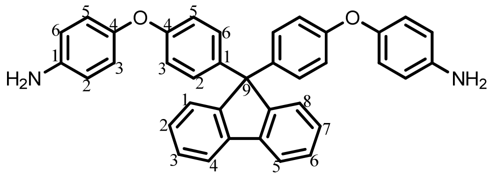

Scheme S5. Molecular structure of *cardo*-AP-BPFL with numbering.

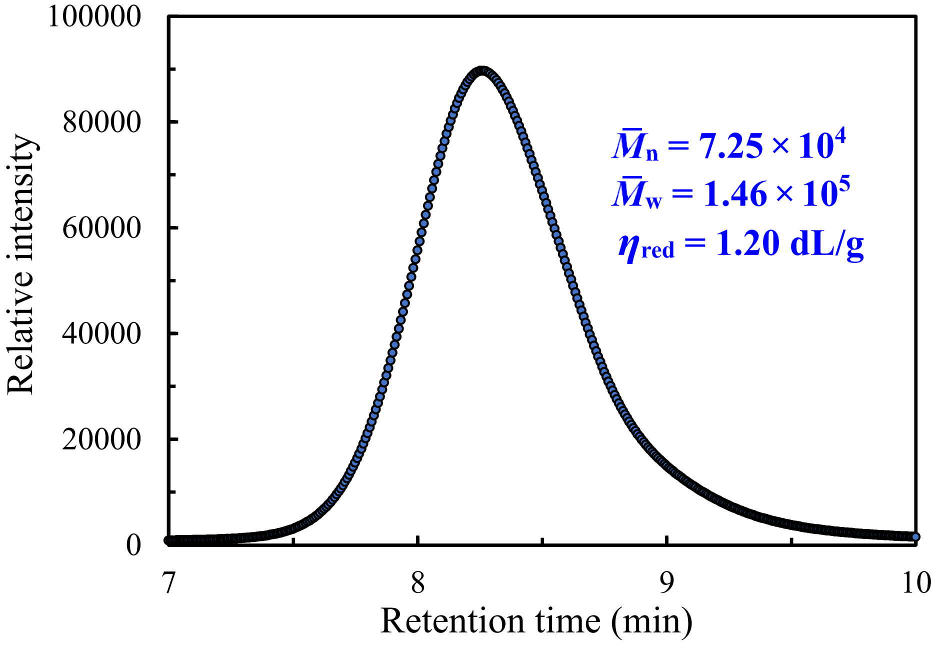

Figure S2. GPC curve for CBDA(50);*spiro*-TA-FLX(50);TFMB copolymer.

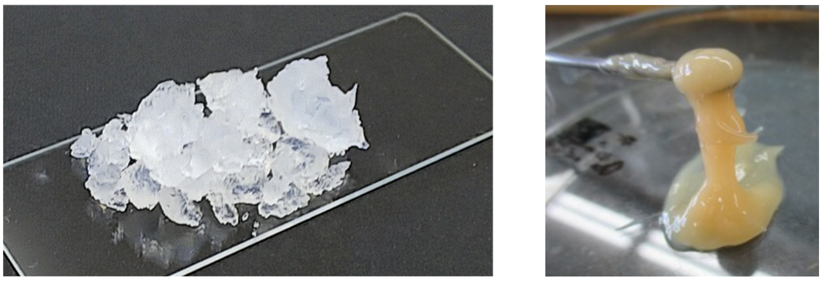

(a) (b)

Figure S3. Incompatibility with Route-C and Route-R for the CBDA/TFMB system: (a) appearance of gelation during chemical imidization in DMAc and (b) a viscous and inhomogeneous reaction mixture just after one-pot polymerization in NMP.

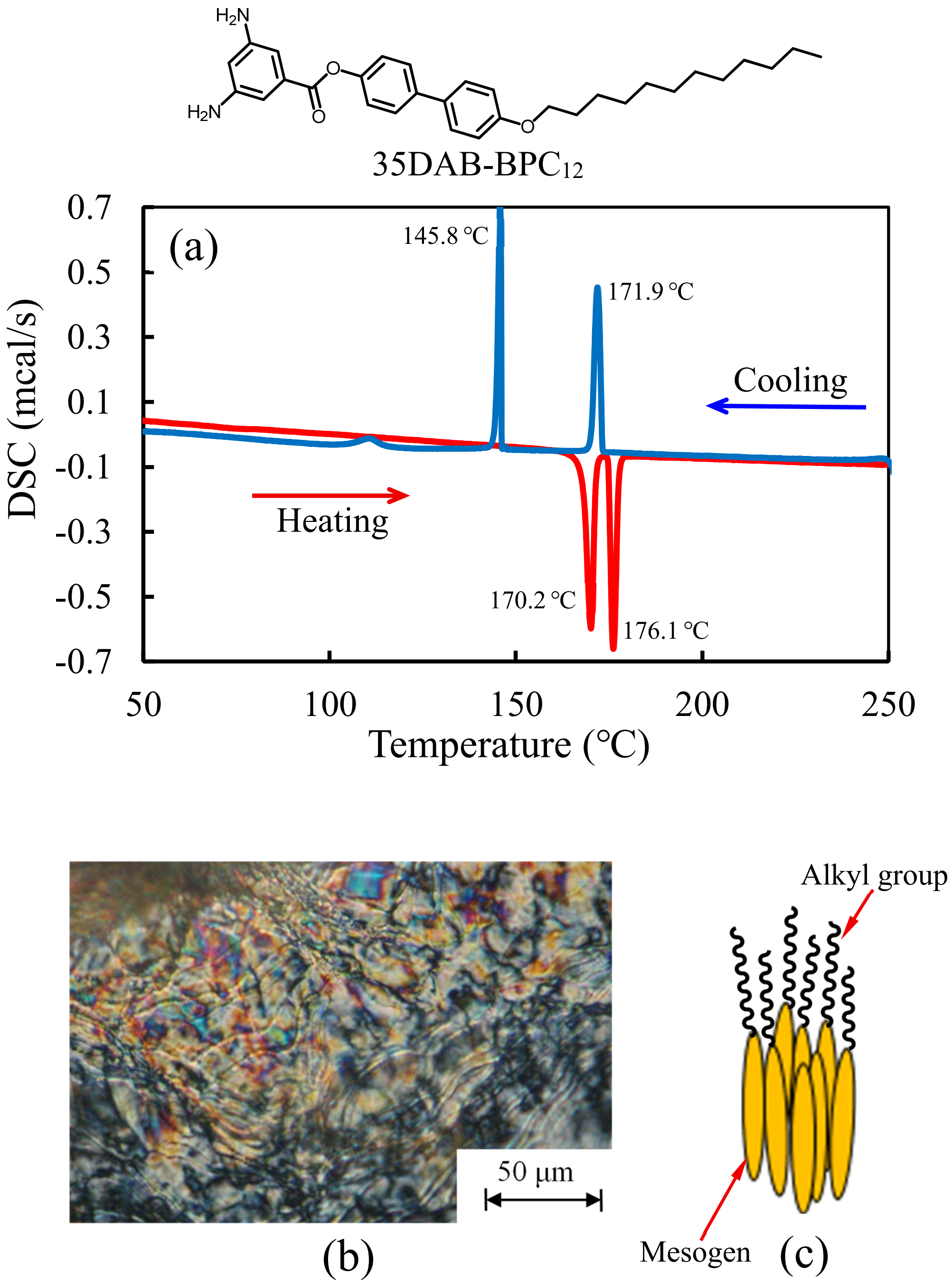

Figure S4. DSC thermogram of 35DAB-BPC_{12} (a) and polarizing optical microscope (POM) photograph taken at 172.8 °C during the heating process (LC ranges: 172.8–178.4 °C during the heating process, 176.4–165.5 °C during the cooling process) (b) and a typical stacked structure between the mesogenic units (c).

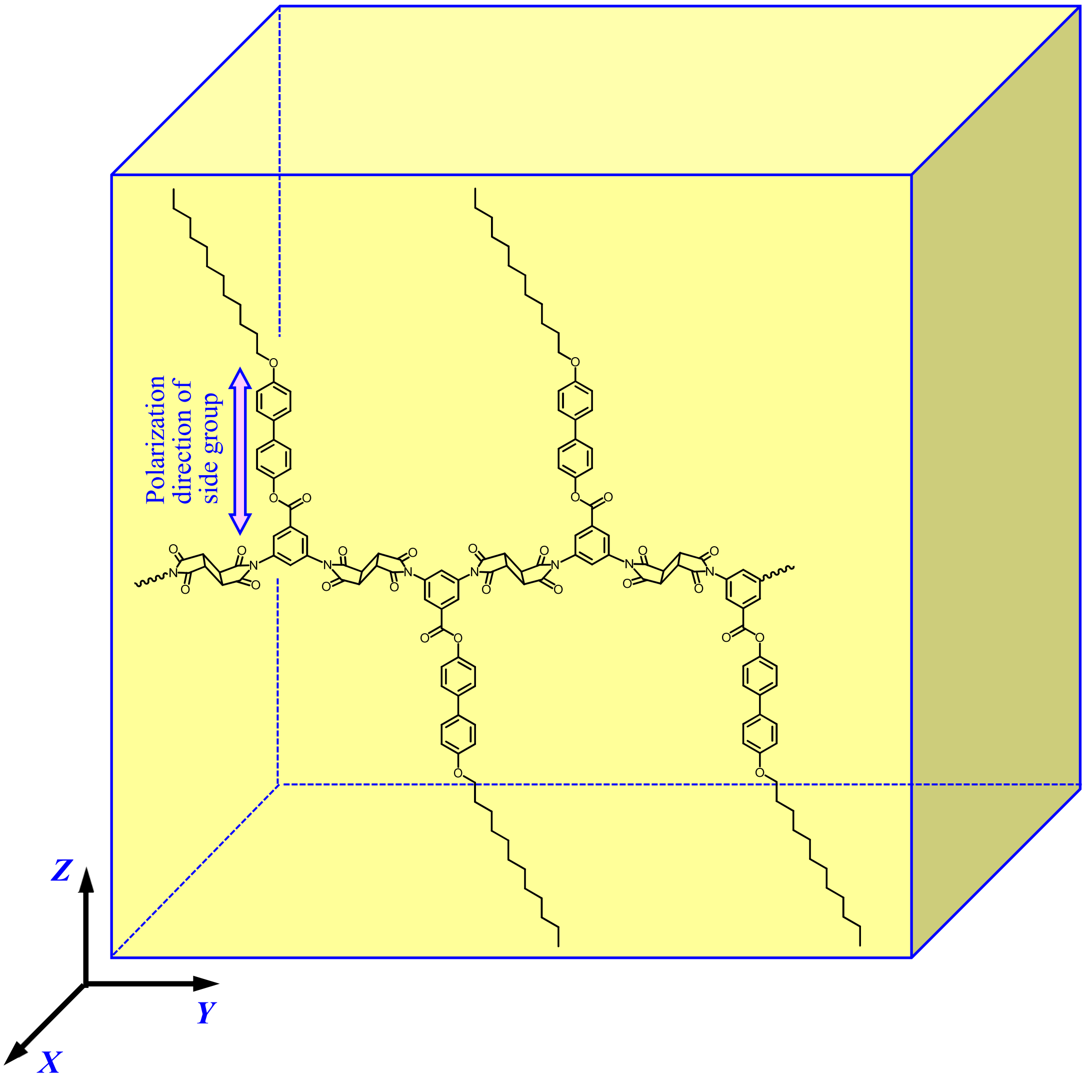

Figure S5. A schematic drawing of expected vertical alignment for the 35DAB-BPC12 unit connected to the CBDA-based PI chains.

Table S2. Results of qualitative solubility test at about 1 wt% using PI powder samples obtained via Route-C or Route-R.

No.	Tetracarboxylic dianhydride	Diamine	NMP	DMAc	DMF	DMSO	GBL	CPN	*m*-cresol	THF	TriGL	EtAc
			Heating temperature (°C) at the 2nd step									
			150	150	140	150	150	130	150	60	150	80
3C	H′-PMDA	TFMB	++	++	++	++	++	++	++	++	++	–
4C	H′-PMDA	TFMB (80)<br>35DAB-BPC$_{12}$ (20)	++	++	++	++	++	++	++	++		+
5C	H′-PMDA	TFMB (70)<br>35DAB-BPC$_{12}$ (30)	++	++	++	++	++	++	++	++		±
6C	CBDA (50)<br>H′-PMDA (50)	TFMB (50)<br>35DAB-BPC$_{12}$ (50)	++	++	+	+	++	+	–	–	–	–
9C	CBDA (70)<br>*cardo*-TA-BPFL (30)	TFMB	++	+	+	+	–	–	–	–	–	–
10C	CBDA (50)<br>*cardo*-TA-BPFL (50)	TFMB	++	++	++	++	++	++	++	++	++	–
11C	*cardo*-TA-BPFL	TFMB	++	++	++	++	++	++	++	++	++	–
13C	CBDA	TFMB (50)<br>*cardo*-AB-BPFL (50)	++	++	++	++	++	–	–	–	–	–
16C	CBDA	TFMB (50)<br>*cardo*-AB-BCFL (50)	++	++	++	++	++	++	±	–	±	–
17C	CBDA	*cardo*-AB-BCFL	++	++	++	++	++	++	±	+	±	–
20C	CBDA	TFMB (50)<br>*spiro*-AP-FLX (50)	+	+	+	±	+	–	–	–	–	–
21C	CBDA	*spiro*-AP-FLX	++	++	++	++	–	–	–	–	–	–
23C	CBDA	TFMB (50)<br>*spiro*-*m*AP-FLX (50)	++	++	++	++	++	++	–	–	–	–
25R	CBDA	TFMB (80)<br>*spiro*-TFAP-FLX (20)	++	++	++	++	++	++	++	++	++	–
26C	CBDA	TFMB (50)<br>*spiro*-TFAP-FLX (50)	++	++	++	++	++	++	+	++	–	–
27C	CBDA	*spiro*-TFAP-FLX	++	++	++	++	++	++	++	++	++	–
30C	CBDA (70)<br>*spiro*-TA-FLX (30)	TFMB	+	+	+	+	±	–	–	–	–	–
31C	CBDA (50)<br>*spiro*-TA-FLX (50)	TFMB	++	++	++	++	++	++	++	++	–	–
32C	*spiro*-TA-FLX	TFMB	++	++	++	++	++	++	++	++	++	–
34C	CBDA (70)<br>*spiro*-TA-FLX (30)	AB-TFMB	++	++	+	++	–	–	–	–	–	–
35C	CBDA (50)<br>*spiro*-TA-FLX (50)	AB-TFMB	++	++	++	++	–	–	–	–	–	–
36C	*spiro*-TA-FLX	AB-TFMB	++	++	++	++	++	++	+	++	++	–
38C	CBDA	TFMB (50)<br>*spiro*-AB-FLX (50)	++	++	++	++	+	++	+	+	–	–
39C	CBDA	*spiro*-AB-FLX	++	++	++	++	++	++	+	–	–	–
41C	CBDA	AB-TFMB (50)<br>*spiro*-AB-FLX (50)	++	++	+	++	+	–	–	–	–	–
42C	CBDA	*t*-CHDA (50)<br>*spiro*-AB-FLX (50)	++	++	++	++	++	–	++	–	–	–
43C	CBDA	TFMB (50)<br>*a*-*spiro*-AB-FLX (50)	++	++	++	++	+	+	+	–	–	–

(++) Soluble at room temperature, (+) soluble when heated at established temperatures and homogeneous after cooling to room temperature, (±) swelling or partially soluble upon heating, and (–) insoluble even after heating. TriGL: triglyme, EtAc: ethyl acetate.

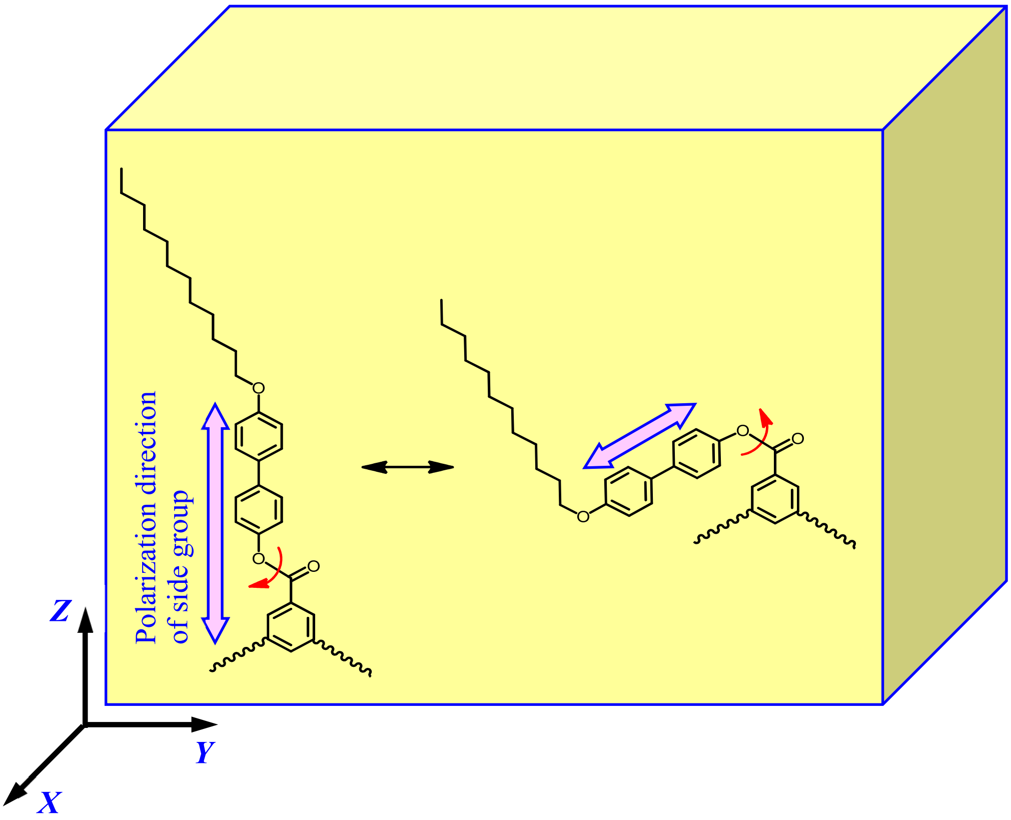

Figure S6. A schematic drawing for disturbed vertical alignment due to internal rotation at the ester group in the 35DAB-BPC12 unit incorporated into the PI main chains.

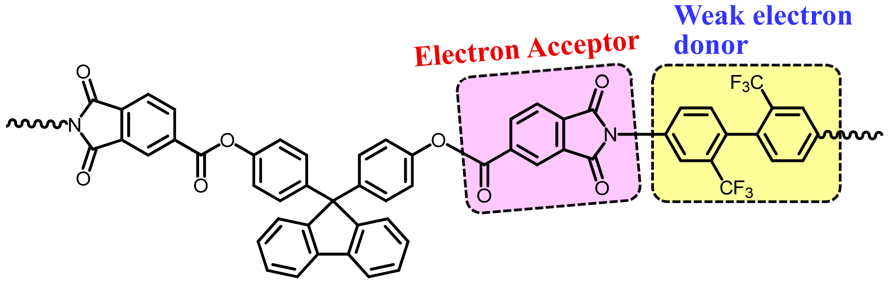

(a)

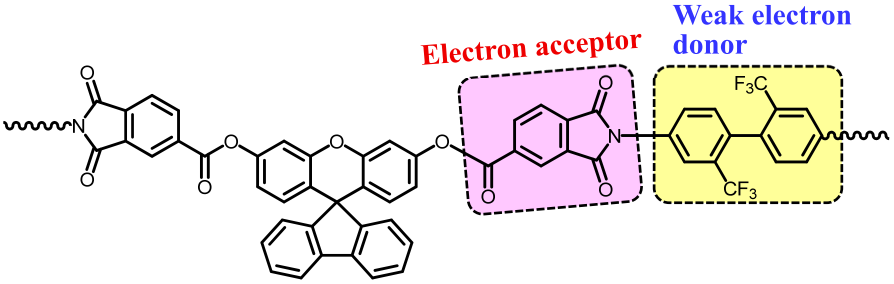

(b)

Figure S7. Possible intramolecular (through-bond) electron donor–acceptor interactions generated in the CBDA/TFMB-based PI systems using TCDA modifiers: (a) *cardo*-TA-BPFL and (b) *spiro*-TA-FLX.

Document S4. An amide-type fluorinated diamine (AB-TFMB) was synthesized according to the procedures described in our previous studies [32,37,41,47]. The analytical data are as follows. T_m = 317 °C (DSC). FT-IR (KBr plate method, cm^{-1}): 3418 (NH_2, N–H stretching), 3303 (amine + amide N–H), 3096/3039 (C_{arom}–H), 1655 (amide, C=O), 1509 (1,4-phenylene group), 1311 (C–F). ^{1}H-NMR (400 MHz, DMSO-d_6, δ, ppm): 10.16 [s, 2H (2.00H), NHCO], 8.33 [d, 2H (2.02H), J = 1.7 Hz, 3,3′-protons of the central biphenylene (BP) unit], 8.07 [dd, 2H (1.97H), J = 8.4, 1.6 Hz, 5,5′-protons of BP], 7.77 [d, 4H (4.05H), J = 8.6 Hz, 3,3′,5,5′-protons of AN], 7.32 [d, 2H (2.03H), J = 8.4 Hz, 6,6′-protons of BP], 6.64 [d, 4H (4.02H), J = 8.5 Hz, 2,2′,6,6′-protons of AN], 5.86 [s, 4H (3.99H), NH_2]. Elemental analysis, Anal. Calcd. (%) for $C_{28}H_{20}O_2N_4F_6$ (558.47): C, 60.22; H, 3.61; N, 10.03. Found: C, 60.00; H, 3.87; N, 9.98. The results confirm that the product is the desired diamine (AB-TFMB, Scheme 6).

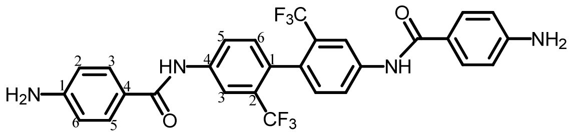

Scheme S6. Structure of AB-TFMB with numbering.

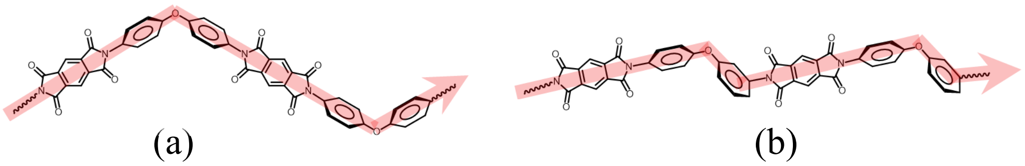

(a) (b)

Figure S8. Schematically depicted main-chain linearity of the extended forms (red-arrows) for isomeric PIs: (a) PMDA/4,4′-ODA and (b) PMDA/3,4′-ODA.

Table S3. Results of qualitative solubility test at about 1 wt% using PI powder samples obtained via Route-C or Route-R.

**No.**	**Tetracarboxylic dianhydride**	**Diamine**	**NMP**	**DMAc**	**DMF**	**DMSO**	**GBL**	**CPN**	***m*-cresol**	**THF**	**TriGL**	**EtAc**
			**Heating temperature (°C) at the 2nd step**									
			**150**	**150**	**140**	**150**	**150**	**130**	**150**	**60**	**150**	**80**
45C	CBDA	TFMB (30)<br>*spiro*-C_6X-DAFL (70)	+	–	–	–	–	–	–	–	–	–
46C	CBDA	*spiro*-C_6X-DAFL	++	++	++	+	+	+	+	–	–	–
47C	CBDA	TFMB (50)<br>*spiro*-C_8X-DAFL (50)	+	–	–	–	–	–	–	–	–	–
48C	CBDA	TFMB (30)<br>*spiro*-C_8X-DAFL (70)	+	–	–	–	–	–	–	–	–	–
50C	CBDA	TFMB (50)<br>*spiro*-TFBzX-DAFL (50)	++	++	++	+	+	+				
51C	CBDA	TFMB (30)<br>*spiro*-TFBzX-DAFL (70)	++	++	++	+	+	+	+ [a]			
52C	CBDA	*spiro*-TFBzX-DAFL	++	++	++	+	+	+	–	–	–	–
53R	CpODA	TFMB	++	++	++	+	++	+	+	+	++	–
54R	CpODA	TFMB (70)<br>*spiro*-TFBzX-DAFL (30)	++	++	++	++	++	+	+	+	±	–
55R	CpODA	TFMB (50)<br>*spiro*-TFBzX-DAFL (50)	++	++	++	++	++	+	+	+	±	–
56R	CpODA	TFMB (30)<br>*spiro*-TFBzX-DAFL (70)	++	++	++	++	++	+	+	+	±	–
57R	CBDA (20)<br>CpODA (80)	TFMB (50)<br>*spiro*-TFBzX-DAFL (50)	++	++	++	++	++	+	+	–	–	–
58R	CBDA (20)<br>CpODA (80)	TFMB (30)<br>*spiro*-TFBzX-DAFL (70)	++	++	++	+	+	+	+	±	–	–
60R	CBDA (20)<br>CpODA (80)	TFMB (50)<br>*spiro*-TFBzX-DAFL (50)	++	++	++	++	++	+	+	–	–	–
61R	CBDA (20)<br>CpODA (80)	TFMB (30)<br>*spiro*-TFBzX-DAFL (70)	++	++	++	+	+	+	+	±	–	–
62R	CpODA	TFMB (70)<br>*spiro*-BzX-DAFL (30)	++	++		++	++	+				
64R′	CpODA (70)<br>*spiro*-2367XADA (30)	TFMB	++	++	++	+	+	+	++	–	–	–
65R	CpODA (50)<br>*spiro*-2367XADA (50)	TFMB	++	++		+	+	–				
66R	*spiro*-2367XADA	TFMB	–	–	–	–	–	–	+	–	–	–
69C	*cardo*-BPFLDA	TFMB	++	++		++	++	++				

(++) Soluble at room temperature, (+) soluble when heated at established temperatures and homogeneous after cooling to room temperature, (±) swelling or partially soluble upon heating, and (–) insoluble even after heating. TriGL: triglyme, EtAc: ethyl acetate.
